# Low‐Symmetry Face‐Capped Fe(II) Tetrahedra Through Anisotropic Ligand Extension

**DOI:** 10.1002/anie.202503473

**Published:** 2025-03-16

**Authors:** Jacob A. Gome, Zack T. Avery, Nina R. Lawson, Oliver G. Stansfield, Jack D. Evans, Michael G. Gardiner, Timothy U. Connell, Dan Preston

**Affiliations:** ^1^ Research School of Chemistry Australian National University Canberra ACT 2601 Australia; ^2^ Centre for Sustainable Bioproducts Faculty of Science Engineering and Built Environment Deakin University Geelong Victoria 3220 Australia; ^3^ School of Physics Chemistry and Earth Sciences The University of Adelaide Adelaide SA 5005 Australia

**Keywords:** Cage structures, Iron, Low‐symmetry, Metallo‐supramolecular, Self‐assembly

## Abstract

Low‐symmetry cages are attractive metallo‐supramolecular targets, as they may possess different characteristics to their higher symmetry analogs. There are no current generalizable routes for the formation of low‐symmetry face‐capped tetrahedra. We report here a strategy using tritopic *tris*‐bidentate ligands with arms of different lengths to access novel tetrahedra. The use of “isosceles” ligands (two arms the same, one different) gives tetragonal disphenoid structures, while a “scalene” ligand (all three arms different) gives a rhombic disphenoid structure. In this last case, diastereoselectivity is also achieved. Distortion away from a perfect tetrahedron resulted in alteration of the character of the respective cage. More distorted cages were more prone to loss of structural integrity upon introduction of highly coordinating dimethyl sulfoxide solvent into the cage in acetonitrile solution. As well, increasing distortion was shown to increase the ease of oxidation from Fe(II) to Fe(III) within the cages.

## Graphical abstract

A strategy for the formation of low‐symmetry face‐capped Fe(II) tetrahedra using anisotropic ligand extension is reported. The degree of distortion in the resulting tetrahedra correlates to the stability of the assemblies and the oxidation potentials of their metal ions.

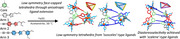

## Introduction

Metallo‐supramolecular assembly has allowed the formation of varied structures of sizes rivaling those of naturally occurring large molecules such as proteins,^[^ ^1^, ^2^, ^3^, ^4^, ^5^, ^6^ ^]^ and often targeting similar functions.^[^ ^7^, ^8^, ^9^, ^10^, ^11^, ^12^, ^13^ ^]^ However, a key difference between natural and synthetic molecules in this area has traditionally been the high symmetry observed in synthetic systems as opposed to their natural analogs, which are instead often characterized by low symmetry and structural diversity. This highly varied structural composition is the pathway by which these naturally occurring molecules achieve their astounding specificity and selectivity of function. For example, proteins can bind metal ions in carefully constructed environments that result in enforced coordination geometries deviating from those considered to be “ideal” and thereby influence their properties and behavior.^[^ ^14^ ^]^

In recent times, there has been a concerted effort by chemists to develop methodologies that likewise give synthetic molecules higher complexity.^[^ ^15^, ^16^, ^17^, ^18^, ^19^, ^20^, ^21^ ^]^ This can be accomplished through three main approaches: 1) the formation of heterometallic structures,^[^ ^22^, ^23^, ^24^, ^25^, ^26^, ^27^, ^28^, ^29^ ^]^ 2) the formation of heteroleptic structures from multiple different ligand types,^[^ ^30^, ^31^, ^32^, ^33^, ^34^, ^35^, ^36^ ^]^ and 3) the use of a single ligand type coming together in a controllable fashion to form homometallic, homoleptic assemblies that are nonetheless low symmetry.^[^ ^37^, ^38^, ^39^, ^40^, ^41^, ^42^ ^]^

For octahedral metal ions, there has been considerable success for the first two of these three approaches. In the formation of heterometallic systems, generally, a metallo‐ligand approach has been adopted, where an inert metal is combined with an organic ligand to form a metal complex with unoccupied sites that can then be combined with a more labile metal ion to form the assembly. Examples of this sort have been reported by Lu et al.,^[^ ^43^ ^]^ Wang et al.,^[^ ^44^ ^]^ Wang et al.,^[^ ^45^ ^]^ Jiang et al.,^[^ ^46^ ^]^ and Metherell and Ward.^[^ ^47^ ^]^ Other approaches to introduce different octahedral metal ions have also been developed, such as the recent report by Shi et al., which exploited intramolecular cation–π interactions to controllably locate labile Zn(II) and Cu(II) ions in segregated locations within the architecture.^[^ ^48^ ^]^

In the formation of heteroleptic assemblies with octahedral metal ions, different ligands with complementary “edge length” distances between metal chelating sites have been used in recent work from Davies and coworkers, where the matching of these edge lengths allowed the assembly of heteroleptic Zn(II) triangular and tetragonal prisms.^[^ ^49^ ^]^ A different approach was taken by Riddell and co‐workers in the formation of an Fe(II) tetrahedron.^[^ ^50^ ^]^ First, subcomponent self‐assembly was used to synthesize a [Fe(L)]^2+^ metalloligand with three vacant bidentate arms, which could be combined with three *bis*‐bidentate ligands and three additional Fe(II) metal ions to form the [Fe_4_(L)(L’)_3_]^8+^ heteroleptic structure.

For the third class, with octahedral metal ions and a single type of ligand for the generation of lower‐symmetry architectures, Nitschke and co‐workers were able to use perfluorinated spacers in high‐symmetry ditopic *bis*‐bidentate ligands to form structures, in which the Fe(II) coordinative environments were all in *tris*‐bidentate *mer* environments rather than the customary *fac* arrangement found in most polynuclear supramolecular assemblies.^[^ ^51^ ^]^ This resulted in the formation of tetragonal, pentagonal, or hexagonal prisms rather than the regular tetrahedra obtained for the nonfluorinated analogs.

The use of a single ligand type for the formation of lower‐symmetry tetrahedra has proven more difficult. In part, this probably results from a difference in the required approach between square planar metal ions (where most work with low symmetry ligands has been carried out) and their octahedral counterparts. With square planar metals, coordination sphere engineering and complementary geometries regarding donor atom directionality have meant that synthetic methods have now been well established, in contrast to methodologies for octahedral metal ions. Nitschke and co‐workers reported the generation of equilibrium mixtures of T, S_4_, and C_3_ symmetry tetrahedra, and were able to perturb the position of the equilibrium through methyl‐substitution of the ligands,^[^ ^52^ ^]^ but were unable to cleanly form a single isomer. Work from Clegg and coworkers has shown that the introduction of substituents into the phenyl core of *bis*‐bidentate ligands results in complex isomeric mixtures of tetrahedra.^[^ ^53^, ^54^ ^]^ Hooley and coworkers were able to use a hydroxyl‐substituted prochiral ligand to access a supramolecular “sorting‐hat” tetrahedron with one *fac* Fe(II) center and three *mer* centers,^[^ ^55^ ^]^ driven by hydrogen bonding to an encapsulated perchlorate anion. Generalizable methods for the quantitative synthesis of low‐symmetry face‐capped tetrahedra from octahedral metal ions^[^ ^34^, ^56^, ^57^, ^58^] have not been developed, in fact, to the best of our knowledge, no examples have been reported.

**Figure 1:**
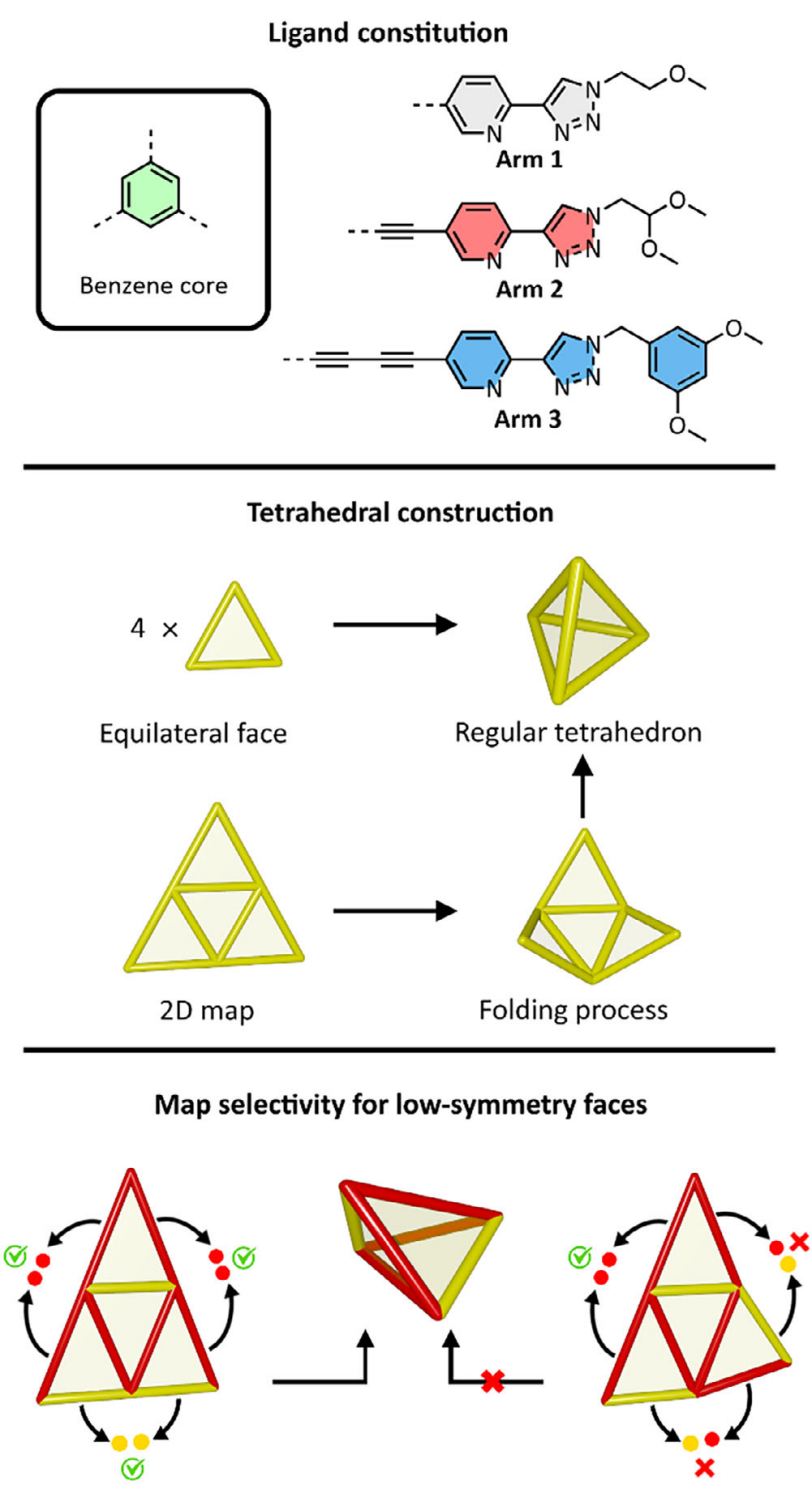
Top: core and the three different arms employed in this study. Middle: representation of the 2D map of a tetrahedron and how it can be theoretically folded into the 3D tetrahedral shape, shown for equilateral faces. Bottom: map selectivity for low‐symmetry faces, showing that the requirement for matched edges entails that only one 2D map can fold into a “tetrahedral” polyhedron.

Tetrahedra,^[^ ^59^ ^]^ either edge‐capped [M_4_(L)_6_]^n+^ or face‐capped [M_4_(L)_4_]^n+^ species, are an incredibly important class of metallosupramolecular architecture,^[^ ^60^, ^61^, ^62^, ^63^, ^64^, ^65^ ^]^ and routes to the synthesis of lower symmetry analogs are a key step in the development of molecular complexity. We report here a strategy for the anisotropic distortion of tritopic *tris*‐bidentate ligands by altering the length of their arms (Figure 1). By combining different arm lengths, we were able to generate “triangular” ligands that were either equilateral, isosceles, or scalene. These ligands could be combined with Fe(II) to form face‐capped “tetrahedra,” which for isosceles and scalene ligands were lower symmetry, namely tetragonal or rhombic disphenoids, respectively. Interestingly, the rhombic disphenoid from the scalene ligand showed differentiation and quantitative selectivity between the two potential diastereomers. Our approach demonstrates an easily accessible and generalizable route to the formation of these lower‐symmetry polyhedra. Furthermore, we demonstrate that divergence from regular polyhedral geometry impacts the character of the resultant cages, both in terms of their stability toward highly coordinating solvents and the oxidation potential of their metal ions.

## Results and Discussion

### Ligand Design, Synthesis, and Nomenclature

All ligands were *tris*‐bidentate tritopic, with a 1,3,5‐substituted benzene core. The bidentate site in all cases was a 2‐pyridyl‐1,2,3‐triazole unit. The triazole unit was substituted with either a monomethyl‐ethylene glycol unit (**Arm 1**), a dimethyl acetal unit (**Arm 2**), or a 3,5‐dimethoxy benzyl unit (**Arm 3**; Figure 1). These different substitutions served to first provide ligands and their complexes with enhanced solubility and second to provide distinct ^1^H NMR (nuclear magnetic resonance) “handles” for 2D NMR analysis. The Arms 1–3 further differed from one another in the length of their linkage to the benzene core. **Arm 1** was directly connected to the core through a carbon–carbon bond, **Arm 2** through an alkyne linker, and **Arm 3** through a butadiyne linker. Hence, all three arms were of different lengths to one another.

**Figure 2:**
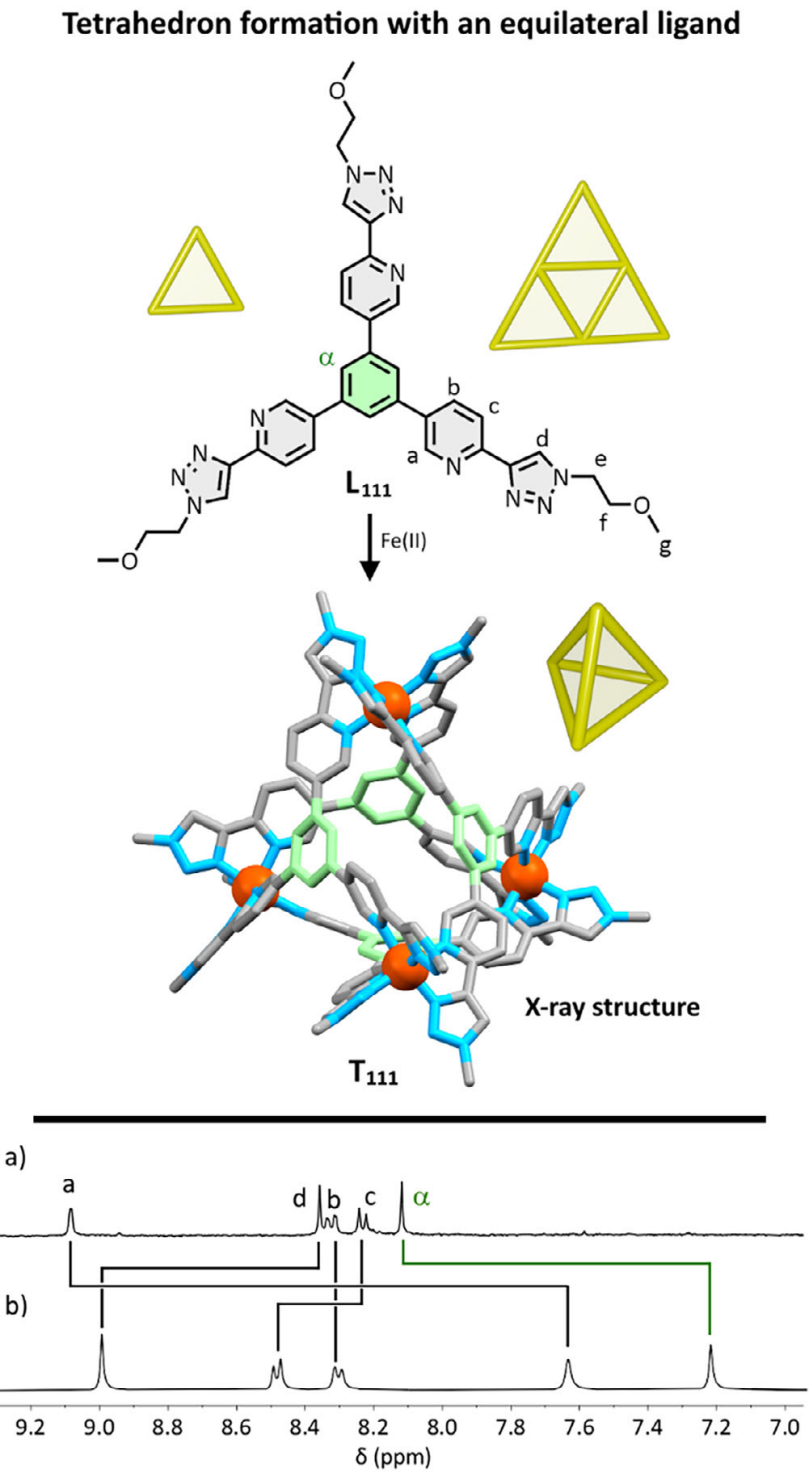
Top: chemical structure of equilateral ligand **L_111_**, which upon treatment with [Fe(H_2_O)_6_](BF_4_)_2_ gives regular tetrahedron **T_111_** ([Fe_4_(**L_111_**)_4_](BF_4_)_8_) The representation of **T_111_** is from the X‐ray crystal structure, with solubilizing chains (where they could be modeled) curtailed to a single carbon atom. Any counterions and all hydrogen atoms are omitted for clarity. Colors: carbon grey for **Arm 1**, green for core, nitrogen light blue, iron orange. Bottom: Partial ^1^H NMR spectra (298 K, 400 MHz, [D_3_]acetonitrile) for a) **L_111_** and b) **T_111_**.

**Figure 3:**
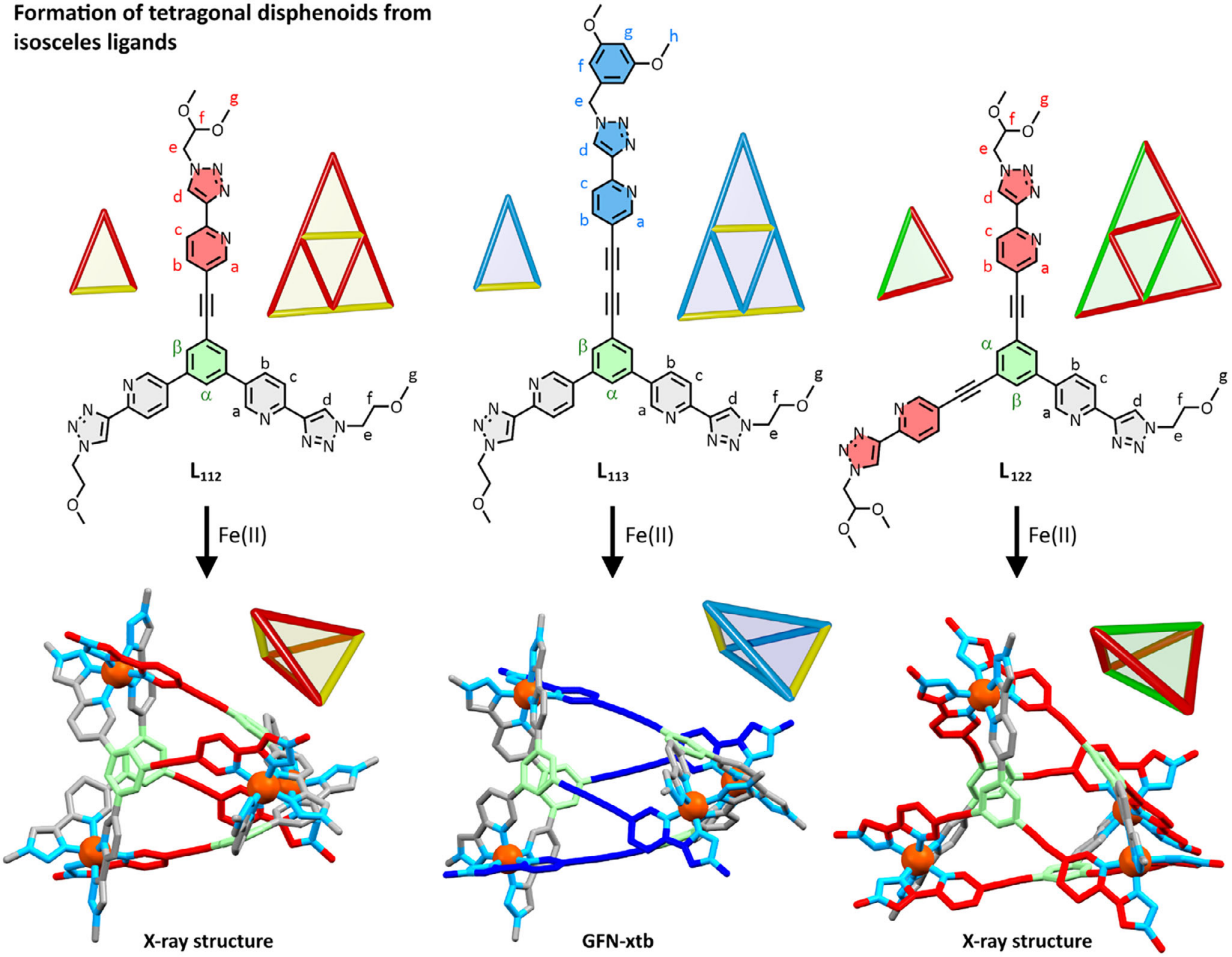
Scheme caption. Depictions of the three isosceles ligands used in this study (L_112_, L_113_, and L_122_), which when combined with [Fe(H_2_O)_6_](BF_4_)_2_ in [D_3_]acetonitrile gave [Fe_4_(L)_4_](BF_4_)_8_ tetragonal disphenoid structures T_112_, T_113_, and T_122_, respectively. Depictions of the complexes are from X‐ray crystal structures (T_112_, T_122_) or calculations using GFN‐xtb (T_113_). Solubilizing chains (where they could be modeled) are curtailed to a single carbon atom, and counterions and hydrogen atoms are omitted, for clarity. Colors: carbon grey for Arm 1, red for Arm 2, blue for Arm 3, green for core, nitrogen light blue, and iron orange.

The ligands were named for the identity of their arms. Hence, **L_111_** had three **Arm 1** units (Figure 2), **L_112_** had two **Arm 1** units (Figure 3), and one **Arm 2** unit, and **L_123_** had one of each type of arm length (Figure 5), and so forth. This design not only gave ligands arms of different lengths but also, when conceiving of a particular ligand as a triangle, sides of different lengths. For example, **L_112_** has a (1,1) side that spans two **Arm 1** units and two (1,2) sides that span between an **Arm 1** and an **Arm 2** unit. Through combining different arms, ligands could be synthesized that were equilateral (all arms lengths the same), isosceles (two arms of one length and one other am), or scalene (all three arm lengths different).

The bidentate sites were all synthesized using CuAAC (copper‐catalyzed azide‐alkyne cycloaddition) “click” chemistry between an azide with the 1H NMR diagnostic handle and 2‐ethynylpyridine (deprotected in situ). After borylation for Arm 1 or the installation of the appropriate alkyne (Arm 2) or butadiyne (Arm 3), Suzuki or Sonogashira couplings were used to build up the individual ligands in modest yields. The ligands were characterized through NMR and IR spectroscopies, and mass spectrometry (Supporting Information). Five ligands were synthesized: “equilateral” L_111_, “isosceles” L_112_, L_122_, and L_113_, and “scalene” L_123_.

### Face‐Capped Tetrahedron From an “Equilateral”‐Type Ligand

Our first goal was to establish that face‐capped tetrahedra could be synthesized using our general ligand design. We accordingly used the simple “equilateral” ligand **L_111_** with three equal arms. The combination of this ligand with [Fe(H_2_O)_6_](BF_4_)_2_ in a 4:4 ratio in [D_3_]acetonitrile led to the formation of the complex in less time than that required to run the ^1^H NMR spectrum. There was a single set of peaks per ligand environment observed, generally, downfield shifted in comparison to the “free” ligand (Figure 2), indicating the formation of a single, high‐symmetry product. The exception from the coordinative environment was the H(a) resonance *ortho* to the coordinating pyridyl nitrogen, which orientates toward the adjacent pyridyl aromatic ring.^[^ ^66^ ^]^ All peaks were diffusing at the same rate from ^1^H DOSY NMR, 3.8 × 10^−10^ m^2^ s^−1^, corroborating this (Supporting Information). The assemblies in this study diffused at rates from 3.5–6.2 × 10^−10^ m^2^ s^−1^, corresponding to hydrodynamic radii of 11–19 Å, consistent with cage formation within the uncertainties imposed by shape, charge, solvent shells, and anions. Mass spectrometry on the sample revealed a series of peaks corresponding to [Fe_4_(**L_111_**)_4_](BF_4_)_n_^(8–n)+^ species (**T_111_**). For example, the peak corresponding to *n* = 3 ([Fe_4_(**L_111_**)_4_](BF_4_)_3_^5+^) was observed at *m/z* = 644.5586, which matches the calculated peak (Supporting Information). In combination, the data suggested a [Fe_4_(**L_111_**)_4_]^8+^ tetrahedron where all four metal ions within a single cage had the same optical isomerism and additionally were all *fac* in their ligand arrangements, i.e., either ΔΔΔΔ or ΛΛΛΛ. The diffusion of diethyl ether into an acetonitrile solution of **T_111_** gave red block crystals from which data was solved and refined that confirmed the formation of the racemic mixture of the tetrahedron (Figure 2, *P*2_1_/*n*, *R*_1_ = 12.5%).^[^ ^67^ ^]^

As with other X‐ray crystal structures detailed in this report, solubilizing chains were often disordered or unable to be modeled; however, the core cationic structure was clear. Without overstating the precision of the data, the Fe(II)–Fe(II) distances within the individual cages ranged from 11.1 to 11.3 Å, showing in a qualitative sense the high symmetry of the structure obtained with the “equilateral” ligand. ^19^F NMR spectroscopy showed that the BF_4_^−^ anion was not “trapped” within the cavity on the NMR timescale: in all crystallography on cages in this report, no encapsulated BF_4_^−^ could be identified, although solvent mask was employed due to diffuse electron density.

### Tetrahedra From “Isosceles”‐Type Ligands

With the capacity to make a high‐symmetry face‐capped tetrahedron with our system confirmed, we now turned to ligands with anisotropy in a single arm, and synthesized three “isosceles”‐type ligands. Ligands **L_112_** and **L_113_** had two **Arm 1** units and a longer arm, **Arm 2** and, **Arm 3**, respectively. The third ligand, **L_122_**, was designed to have only a single short **Arm 1** and then two long **Arm 2** units. For all three ligands, only one feasible 2D map and therefore only one polyhedron was feasible to maintain an equal length of adjacent sides (Figure 3).

**Figure 4:**
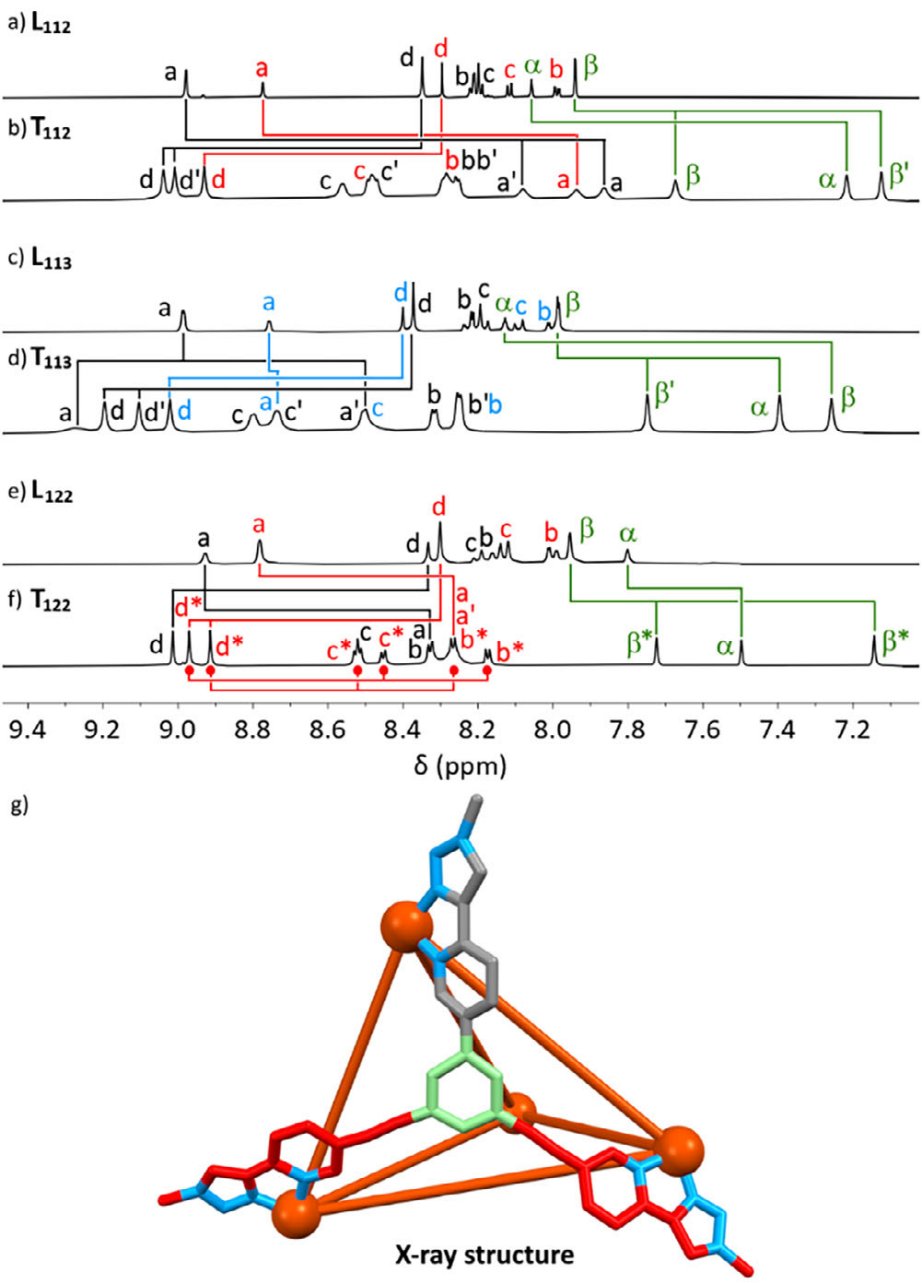
Partial ^1^H NMR spectra (298 K, 400 MHz, [D_3_]acetonitrile) for a) **L_112_** and b) **T_112_**, c) **L_113_** and d) **T_113_**, and e) **L_122_** and f) **T_122_**. Labels are color coded: **Arm 1** black, **Arm 2** red, **Arm 3** blue, core green. Proton environment labels are shown in Figure 3. TOCSY‐derived spin systems aided by NOE correlations for the two distinct **Arm 2** environments are shown under the spectrum for (f) for **T_122_**. Below is shown g) a view of from the X‐ray structure of **T_122_** with only one ligand shown, demonstrating the desymmetrization of the two **Arm 2** environments with respect to **Arm 1** (**Arm 1** shown in grey, **Arm 2** shown in red).

Complexations proceeded as before, with the 4:4 combination of the appropriate ligand with Fe(II) in [D_3_]acetonitrile. With ligands **L_112_** and **L_122_**, equilibrium was swift, for the less soluble **L_113_** ligand, the solution was heated overnight at 50 °C. Mass spectrometry of the samples revealed the formation of their respective [Fe_4_(**L**)_4_]^8+^ tetrahedra, for example for **T_113_**, *m/z* = 616.1826 ([**T_123_ **+ 2BF_4_]^2+^, Supporting Information). The ^1^H NMR spectra (Figure 4) of all spectra shared certain characteristics. First, all peaks in each individual spectrum were diffusing at the same rate (*D*(**T_112_**) = 5.1 × 10^−10^ m^2^ s^−1^, *D*(**T_113_**) = 4.2 × 10^−10^ m^2^ s^−1^, *D*(**T_122_**) = 6.2 × 10^−10^ m^2^ s^−1^), suggesting the formation of a single or multiple species all the same hydrodynamic radii in each solution. Second, each spectrum had a distinct set of peaks for all three arms of the ligand, in other words, there was desymmetrization of the arm that was present twice. This is consistent with the formation of a racemic mixture of cages where each cage has the same chirality at each metal of the four metal centers, i.e., ΔΔΔΔ or ΛΛΛΛ, and the two arms, which are equivalent in the ligand become nonequivalent in the complex dependent on their spatial relationship to the third arm (Figure 4). Third, the peaks of the central benzene core integrated into three protons and could be identified as being part of the same molecule through TOCSY correlation (Supporting Information), indicating that while each arm of each ligand was in a distinct environment, all ligands present were chemical equivalent and therefore only one structural type of assembly had formed.

Two of the complexes from isosceles‐type ligands (**T_112_** and **T_122_**) were crystallized, both through vapor diffusion of diethyl ether into acetonitrile solutions of the cages. While the structures were not high quality and the resolution of solubilizing chains was poor, the core cationic structure in each case was nonetheless clear. The refined solutions of these revealed the expected racemic mixture of homochiral cages, organized into lower symmetry polyhedra, namely, tetragonal disphenoids, where one of each of the three different arms came together at each Fe(II) corner (Figure 3). The Fe(II)–Fe(II) distances in each of the structures fell into one pair of longer distances, and a quartet of shorter distances (Supporting Information). For example, (again acknowledging the poor quality of the structure) in **T_122_**, there were two distances of 15.3 to 15.4 Å associated with the two (2,2) sides and from 12.5 to 13.7 Å associated with the four (1,2) sides. We compared the crystallized **T_112_** and **T_122_** structures with modeled structures from GFN‐xtb computations^[^ ^68^ ^]^ and found good structural agreement (Supporting Information). Superimposition of the four Fe(II) centers through root mean square minimization gave distances between calculated and X‐ray Fe(II) centers of 0.144 to 0.193 Å for **T_112_** and 0.260 to 0.280 Å for **T_122_** (Supporting Information). This gave us confidence to use the same modeling approach to obtain a calculated structure for **T_113_** (Figure 3).

### Tetrahedron From a “Scalene”‐Type Ligand

**Figure 5:**
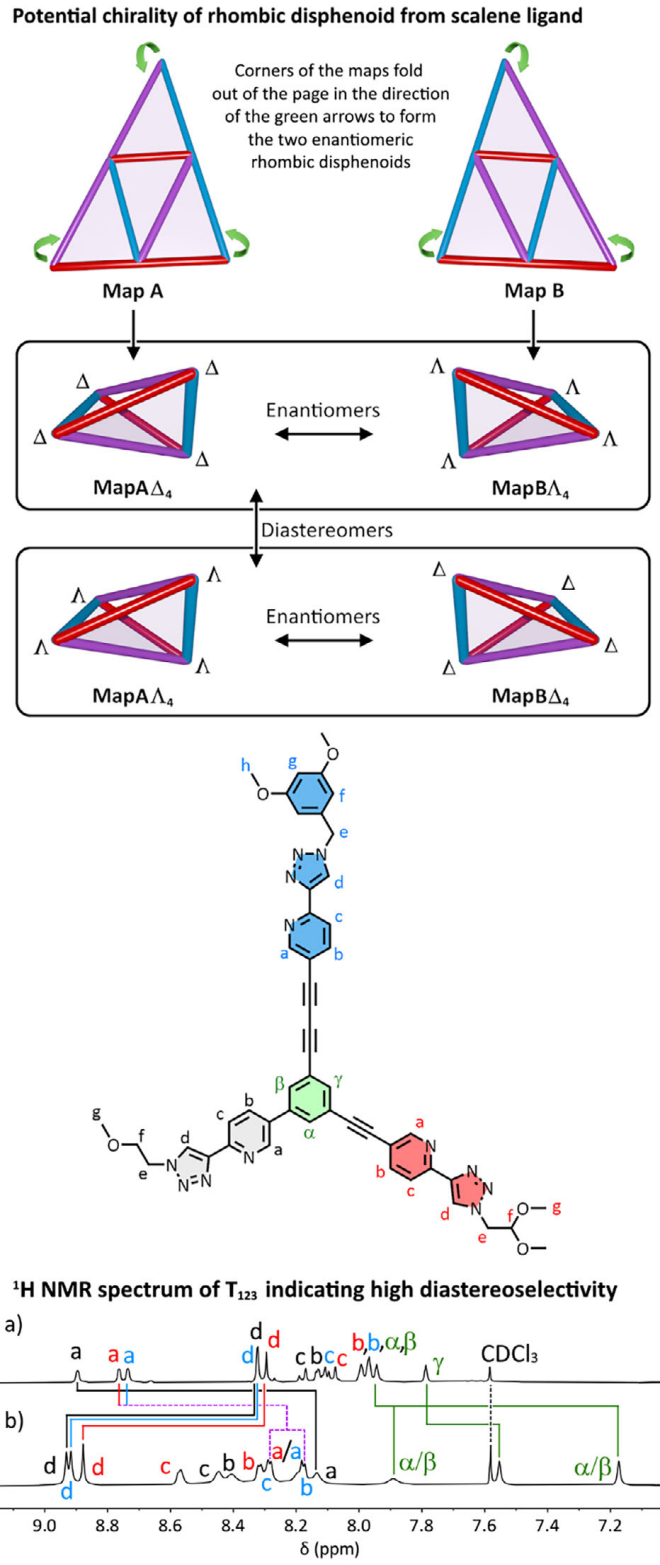
Top: The two chiral 2D maps for **T_123_** (**Map A** and **Map B**) that, when folded with points coming *out* of the page, give two chiral rhombic disphenoids. The combination of these rhombic disphenoids with the ΔΔΔΔ or ΛΛΛΛ homochirality at metal centers gives two enantiomeric pairs that are diastereoisomers, **MapA**Δ_4_ paired with **MapB**Λ_4_, and **MapA**Λ_4_ paired with **MapB**Δ_4_. Middle: The chemical structure of **L_123_**. *Bottom*: Partial ^1^H NMR spectra (298 K, 400 MHz, [D_3_]acetonitrile) of a) **L_123_** and b) **T_123_** showing only a single set of peaks per ligand environment and hence, the diastereoselective formation of a single enantiomeric pair. Labels are color coded: **Arm 1** black, **Arm 2** red, **Arm 3** blue, core green. 2D NMR techniques were unable to distinguish between *α* and *β* environments on the core, or between **Arm 2** Ha) and **Arm 3** H(a) in the cage.

With the capacity to reliably form low‐symmetry [Fe_4_(**L**)_4_]^8+^ polyhedra from isosceles ligands established, the logical next step was the synthesis and complexation of a scalene ligand, **L_123_**, which had one of each different arm type present. An interesting consideration not present with the isosceles ligands now arose: the capability for polyhedral (as opposed to coordinative) chirality. In other words, for ligand **L_123_**, there are two nonsuperimposable 2D maps (**Map A** and **Map B**, see Figure 5) that will, respectively, fold into two mirror‐image rhombic disphenoids with D_2_ symmetry (shown in the figure with point folding *out* of the page). When combined with the capacity for each [Fe_4_(**L**)_4_]^8+^ structure to be either ΔΔΔΔ or ΛΛΛΛ, this results in the possibility of two sets of enantiomers being formed, one pair being **MapA**Δ_4_ and **MapB**Λ_4_, related through diastereoisomerism to the other pair, **MapA**Λ_4_ and **MapB**Δ_4_ (Figure 5). Diastereoisomerism would manifest in the ^1^H NMR spectrum of a mixture as two sets of resonances per unique ligand environment.

The 4:4 combination of **L_123_** with Fe(II) in [D_3_]acetonitrile required heating at 50 °C overnight to equilibrate. The ligand could alternatively be presolubilized in minimal CDCl_3_ before the reaction to help with reaction speed. Mass spectrometry on the sample showed the formation of the [Fe_4_(**L_123_**)_4_]^8+^ tetrahedron, **T_123_**. In the ^1^H NMR spectrum (Figure 5), there was a *single* peak per ligand environment, all diffusing at the same rate via DOSY NMR spectroscopy (Supporting Information), indicating the formation of a single species. This strongly suggested that, serendipitously, one enantiomeric pair formed in energetic preference to the other. We were, unfortunately, unable to crystallize the complex despite multiple attempts, and so turned to GFN‐xtb to try to identify the predominant pair of enantiomers.

**Figure 6:**
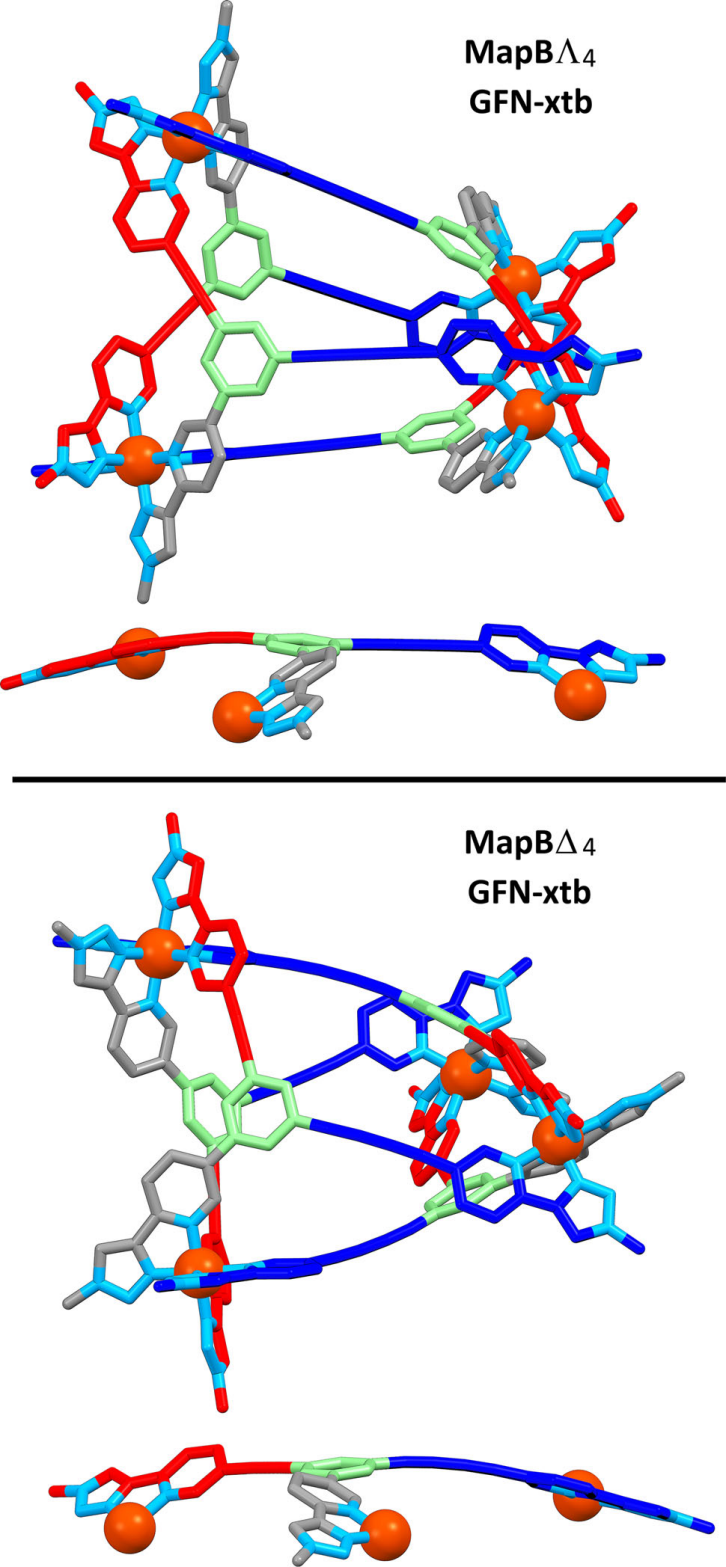
GFN‐xtb optimized structures of **MapB**Λ_4_, (top) and **MapB**Δ_4_ (bottom). Note that **MapB**Λ_4_ is the same in energy as its enantiomer **MapA**Δ_4_, as is **MapB**Δ_4_ with its enantiomer **MapA**Λ_4_ (see Figure 5). Colors: carbon grey for **Arm 1**, red for **Arm 2**, blue for **Arm 3**, green for core, nitrogen light blue, and iron orange. Comparison of the energies between the two structures found that **Map** **B**Λ_4_ was lower in energy by 10 kJ mol^−1^.

Accordingly, we compared the two diastereomers from the rhombic disphenoids obtained from **Map B**: **MapB**Λ_4_, and **MapB**Δ_4_ (Figure 6, Supporting Information). We found that **MapB**Λ_4_ was lower in energy by 10 kJ mol^−1^.

This would equate to an equilibrium constant of *K* **_B_**_D4→_ **_B_**_L4_ = 60, or a diastereomeric ratio of approximately 98:2. Given the fact that only a single species was observed in the ^1^H NMR spectrum, this energy difference seems reasonable. The key difference between the structures of **MapB**Λ_4_ and **MapB**Δ_4_ was that in **MapB**Λ_4_ strain appeared predominantly propagated through the alkyne of **Arm2**, while in **MapB**Δ_4_ there was more distortion in the butadiyne of **Arm3** (Figure 6). There was also a greater degree of distortion away from that of a “perfect” tetrahedron for the less favored **MapB**Δ_4_ structure (see below). We also carried out density functional theory (DFT) calculations. Using the r2SCAN‐3c functional and def2‐SVP (C, H) and def2‐TZVP (N, Fe) basis sets,^[^ ^69^ ^]^ we compared the two diastereomers from the rhombic disphenoids obtained from **Map B**: **MapB**Λ_4_ and **MapB**Δ_4_ (Supporting Information). These gave a similar energetic favorability for **MapB**Λ_4_ of 7 kJ mol^−1^ although the optimized structures did not display any of the significant bending of the arms of the ligands that might be expected. We note that energetic comparison through calculations of large structures with complex thermodynamic landscapes always contain uncertainties and so our assignment of the predominant isomeric pair being **MapA**Δ_4_ and **MapB**Λ_4_ is at best tentative.

### Structure–Activity Trends

We proposed that the different assemblies might have different properties dependent on their relative degrees of distortion away from a “perfect” regular tetrahedron, and considered how this distortion might be measured so that it could be ranked. A polyhedron formed from four triangular faces will have 12 distinct triangular angles (*θ*) (for example, in these cages, Fe–Fe–Fe angles), three per each of the four triangular faces. The average of these angles should be in each case 60°. However, the standard deviation of the distribution of these angles will differ as a polyhedron is perturbed away from regularity. Thus, a regular tetrahedron (**T_111_**) should have a standard deviation of *θ* (SD_θ_) of approximately 0°, while with disphenoids with a greater disparity between edge lengths, higher SD_θ_ values should result. Hence, **T_112_** and **T_122_** with (1,1) or (2,2) and (1,2) side lengths should have higher SD_θ_ values than **T_111_** but lower values than **T_123_** with (1,2), (1,3), and (2,3) side lengths, which should in turn have a lower SD_θ_ than **T_113_** with (1,1) and (1,3) side lengths.

We calculated the SD_θ_ values for the five tetrahedra/disphenoids. These were calculated from the GFN‐xtb optimized structures (Table 1). We found that the ranking of distortion of the [Fe_4_(**L**)_4_]^8+^ assemblies, from least to most, was **T_111_** (SD_θ_ = 0.13°), **T_112_** (SD_θ_ = 7.81°), **T_122_** (SD_θ_ = 8.15°), **T_123_** (SD_θ_ = 12.21°), and **T_113_** (SD_θ_ = 14.25°). These values match the predicted order of distortion discussed above. To corroborate these values, the same calculations were carried out for X‐ray structures (**T_111_**, **T_112_**, and **T_122_**) and the DFT calculated structure of **T_123_** (Supporting Information).

**Table 1:** Properties of assemblies in this study. SD_θ_ is the standard deviation of all Fe‐Fe‐Fe angles in the cage, from GFN‐xtb calculations, cross‐referenced to XRD and DFT structures (Supporting Information).

Assembly	SD_θ_ (°)	Stability (in % [D_6_]DMSO)	Σ (°)	*E*°′_ox_ (Δ*E*) (V)
**T_111_**	0.13	> 25.0	38	1.30 (0.191)
**T_112_**	7.81	12.5	42	1.28 (0.131)
**T_122_**	8.15	10.0	42	1.27 (0.121)
**T_123_**	12.21	7.5	42	1.28 (0.141)
**T_113_**	14.25	5.0	44	1.21 (0.131)

Stability is to which point the intact cage was observed via ^1^H NMR spectroscopy adding [D_6_]DMSO to a solution containing equimolar amounts of each cage. Σ is the summed deviation from 90° for each *cis*‐donor–Fe‐donor angle, from GFN‐xtb, obtained using Octadist.^[^ ^70^ ^]^ *E*°′_ox_ values obtained versus SCE.

The SD_θ_ values were similar regardless of the origin of the structure, giving us confidence that the relative rankings of distortion thus obtained were correct and related to geometric considerations. The closest two values, for **T_112_** and **T_122,_** retained their qualitative ordering in the X‐ray crystal structures with SD_θ_ for **T_112_** of 7.58° and 6.78° (2 distinct disphenoids within the unit cell) and the SD_θ_ value of **T_122_** being 8.85°. The SD_θ_ value for **T_123_** was very similar to that obtained by DFT, 11.92°.

**Figure 7:**
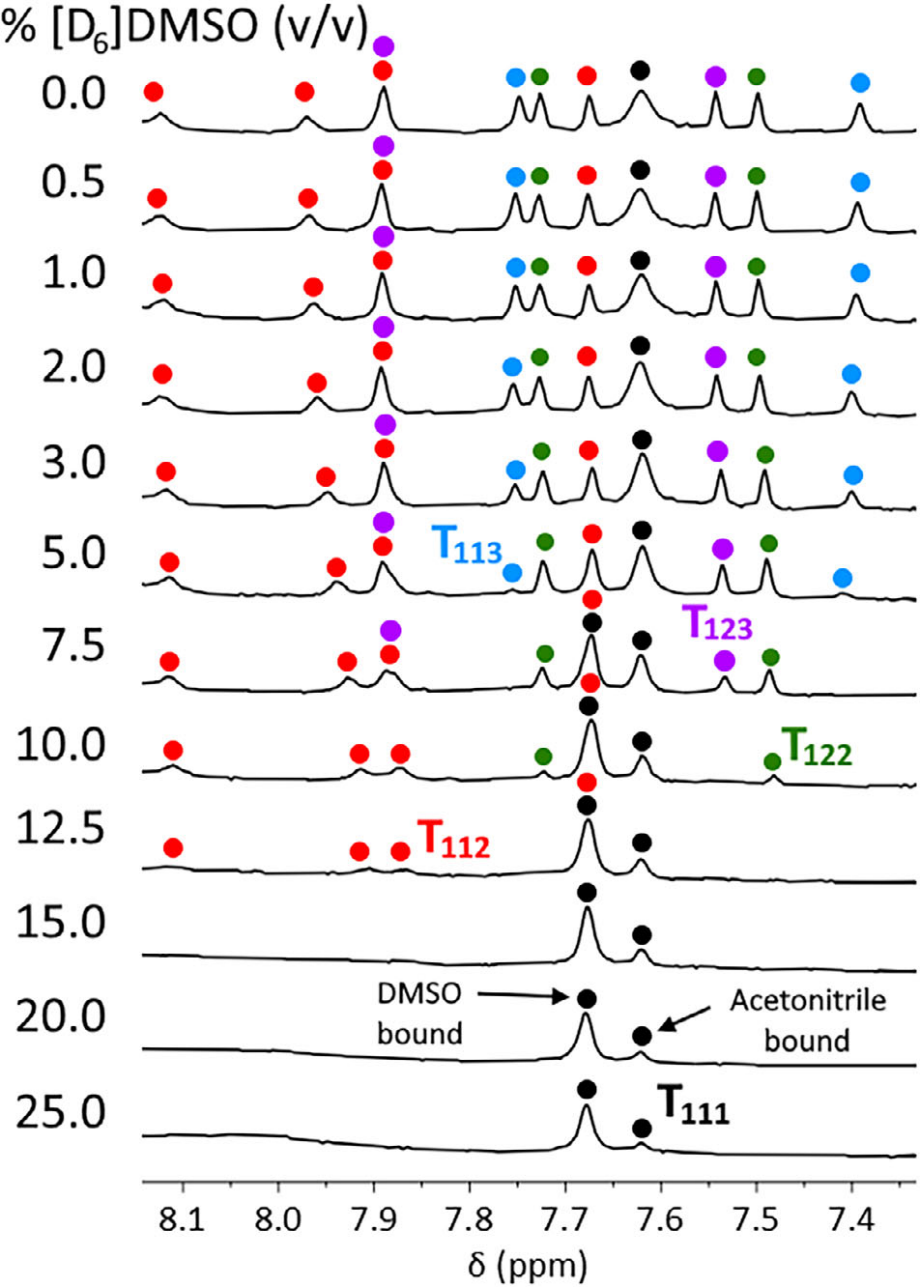
Stability studies carried on via ^1^H NMR spectroscopy (400 MHz, CD_3_CN/[D_6_]DMSO, 298 K) of **T_111_**, **T_112_**, **T_113_**, **T_122_**, and **T_123_**. Cage peak identity is indicated with colored dots: **T_111_** (black), **T_112_** (red), **T_113_** (blue), **T_122_** (green), and **T_123_** (purple). The addition of [D_6_]DMSO resulted in changes to chemical shifts of the cage species. For convenience, the spectra were arbitrarily referenced to the H(a) peak of **T_111_**.

We next investigated the stability of the assemblies. [D_6_]DMSO is known to be capable of competing with pyridyl‐triazole ligands of displacing Fe(II).^[^ ^71^ ^]^ A solution containing equimolar amounts of each cage in [D_3_]acetonitrile slowly had aliquots of [D_6_]DMSO added to it. As the percentage volume of [D_6_]DMSO increased, the resonances of various ligands broadened and disappeared, indicating that cage integrity had been in that case lost and that ligand association with Fe(II) was now highly fluctional (Figure 7, Supporting Information). The order, in which the cage resonances disappeared was the same order from most to least distorted cages, i.e., from high to low SD_θ_ values: peaks for **T_113_** were last observed at 5% [D_6_]DMSO, **T_123_** at 7.5%, **T_122_** at 10.0%, and **T_112_** at 12.5%. The peaks for the regular tetrahedron **T_111_** remained sharp until 25% [D_6_]DMSO at which point shimming the mixed solvent NMR sample with paramagnetic high‐spin Fe(II) became impossible. It, therefore, appears that cage stability is inversely proportional to the degree of distortion from a regular tetrahedron,^[^ ^72^ ^]^ and for the family we explored, the trend was predicted in each case from SD_θ_ values calculated from low‐cost GFN‐xtb structures.

It was also observed that at higher DMSO concentrations the **T_111_** H(a) resonance close to the cavity split into two peaks, with the emergent peak increasing in intensity with increasing DMSO percentage. The other resonances on the cage peripheries remained unsplit. We propose that this splitting probably arises from the exchange of bound acetonitrile for DMSO in the relatively small **T_111_** cavity, although this could not be confirmed through mass spectrometry.

Having established the inverse trend between stability and distortion, we revisited the data from the calculated structures of the diastereomers, **MapB**Λ_4_ and **MapB**Δ_4_. In both cases, we found that the energetically favored structure had a slightly smaller SD_θ_ value than the other (using GFN‐xtb, 12.21° vs. 13.71°, using DFT, 11.41° vs. 12.24°). We note that a comparable difference between the SD_θ_ values of **T**_**112**_ and **T**_**122**_ translated into measurable differences in the stability toward DMSO between these two cages. It, therefore, seems likely to us that ligand strain between the two **MapB**Λ_4_ and **MapB**Δ_4_ isomers with slightly different levels of distortion accounts for the diastereoselectivity observed and discussed above.

In the introduction, we mentioned the use of the coordinative scaffold in metalloproteins to influence the character of the metal ion. We were interested to see if the degree of distortion in these cages might have a similar effect on their Fe(II) metal ions here. We accordingly investigated the oxidation potential of the five assemblies in acetonitrile. Cyclic voltammetry revealed a quasi‐reversible wave assigned to the Fe(III)/Fe(II) potential between 1.20–1.30 V versus SCE for each complex. Measured potentials were ∼0.5 V greater than similar reported structures,^[^ ^73^ ^]^ which we attribute to the relatively electron‐poor pyridyl‐triazole chelates in these systems relative to polypyridyl ligands. A second irreversible oxidative process was observed before reaching the solvent window in the **T**_**113**_ complex (which also had the lowest E°′ value, see below). In terms of the trend for oxidation potentials (Table 1), **T**_**113**_ was the easiest to oxidize (1.21 V), **T**_**111**_ was the most difficult (1.30 V), and the other three cages were tightly banded in between (**T**_**112**_, **T**_**123**_ = 1.28 V, **T**_**122**_ = 1.27 V).

We now sought to analyze this ordering with respect to cage distortion. Our measure of global distortion, SD_θ_, reasonably closely matches the trend of oxidation potentials given above. Analysis of the GFN‐xtb‐obtained structures using Octadist^[^ ^70^ ^]^ allowed ranking of the respective distortion of the coordinative environments (Σ) of their Fe(II) metal ions (Table 1). Unsurprisingly there was reasonable agreement between distortion of the entire structure and the individual metal ions within it. The ranking of the Σ values was, from least to most distorted, **T**_**111**_ < **T**_**112**_ ≈ **T**_**122**_ ≈ **T**_**123**_ < **T**_**113**_. This trend replicates that of the oxidation potentials of the five cages.

Therefore, for this series of compounds, Octadist provided a more accurate predictive ranking of oxidation potentials for the assemblies than SD_θ_, with both being generated from easily calculated GFN‐xtb structures. We attribute this correlation between coordinative distortion and ease of oxidation to the lower level of crystal field stabilization in distorted octahedral geometries, making electron loss easier to accomplish. Being able to controllably tune oxidation potentials of metal ions in supramolecular structures using our strategy here may prove beneficial for other chemists seeking to exploit redox activity in their systems.

## Conclusion

We have successfully developed a generalizable method for the formation of low‐symmetry face‐capped tetrahedra, through the use of tritopic ligands with arms of different lengths. We were able to successfully synthesize three tetragonal disphenoids using ligands with two arms of the same length and one of different lengths, and also an example of a rhombic disphenoid from a ligand with three distinct arms. In this last case, we also achieved diastereoselective control between two different polyhedral isomers. Distorting the assemblies away from the geometry of a “perfect” regular tetrahedron influenced their properties. We established the degree of distortion in the cages to be: **T_111_** (least), **T_112_**, **T_122_**, **T_123_**, and **T_113_** (most). This distortive lowering of symmetry impacts on cage character, both at a “global” level (i.e., cage stability) and also serving to allow tuning of the oxidation behavior of the metal ions within the cage. Importantly, within the family of complexes we made, we were able to correlate both the order of their stability and the order of their oxidation potential using two simple parameters (SD_θ_ for stability and Σ for oxidation potential) derived from low‐cost computations with excellent accuracy, suggesting that these properties could be qualitatively assessed in prospective structures using modeling. We believe that similar approaches for structural design could also be employed toward other polyhedral types, with similar influence upon their character.

## Supporting Information

The authors have cited additional references within the Supporting Information.^[^ ^74^, ^75^, ^76^, ^77^, ^78^, ^79^, ^80^, ^81^, ^82^, ^83^ ^]^

## Conflict of Interests

The authors declare no conflict of interest.

## Supporting information



Supporting information

## Data Availability

The data that support the findings of this study are available from the corresponding author upon reasonable request.

## References

[1] B. E. Barber , E. M. G. Jamieson , L. E. M. White , C. T. McTernan , Chem 2024, 10, 2792–2806.

[2] D. Fujita , Y. Ueda , S. Sato , H. Yokoyama , N. Mizuno , T. Kumasaka , M. Fujita , Chem 2016, 1, 91–101.

[3] D. Fujita , Y. Ueda , S. Sato , N. Mizuno , T. Kumasaka , M. Fujita , Nature 2016, 540, 563–566. 30905932 10.1038/nature20771

[4] Y. Domoto , M. Abe , M. Fujita , J. Am. Chem. Soc. 2021, 143, 8578–8582. 34100600 10.1021/jacs.1c03208

[5] S. M. Jansze , M. D. Wise , A. V. Vologzhanina , R. Scopelliti , K. Severin , Chem. Sci. 2017, 8, 1901–1908. 28567267 10.1039/c6sc04732g PMC5444114

[6] J. A. Davies , T. K. Ronson , J. R. Nitschke , Chem 2022, 8, 1099–1106.

[7] J. I. Virtue , S. Tsoukatos , M. R. Johnston , W. M. Bloch , Chem. Sci. 2024, 15, 19119–19125. 39494370 10.1039/d4sc04913f PMC11525710

[8] R. G. DiNardi , S. Rasheed , S. S. Capomolla , M. H. Chak , I. A. Middleton , L. K. Macreadie , J. P. Violi , W. A. Donald , P. J. Lusby , J. E. Beves , J. Am. Chem. Soc. 2024, 146, 21196–21202. 39051845 10.1021/jacs.4c04846 PMC11311219

[9] V. Marti‐Centelles , A. L. Lawrence , P. J. Lusby , J. Am. Chem. Soc. 2018, 140, 2862–2868. 29406705 10.1021/jacs.7b12146

[10] P. Mal , B. Breiner , K. Rissanen , J. R. Nitschke , Science 2009, 324, 1697–1699. 19556504 10.1126/science.1175313

[11] I. A. Riddell , M. M. Smulders , J. K. Clegg , J. R. Nitschke , Chem. Commun. 2011, 47, 457–459. 10.1039/c0cc02573a 20871932

[12] M. Yamashina , Y. Sei , M. Akita , M. Yoshizawa , Nat. Commun. 2014, 5, 4662. 25130933 10.1038/ncomms5662

[13] P. Das , A. Kumar , P. Howlader , P. S. Mukherjee , Chem. ‐Eur. J. 2017, 23, 12565–12574. 28644555 10.1002/chem.201702263

[14] D. P. Barondeau , E. D. Getzoff , Curr. Opin. Struct. Biol. 2004, 14, 765–774. 15582401 10.1016/j.sbi.2004.10.012

[15] Z. T. Avery , J. L. Algar , D. Preston , Trends Chem 2024, 6, 352–364.

[16] J. E. M. Lewis , J. D. Crowley , ChemPlusChem 2020, 85, 815–827. 32364332 10.1002/cplu.202000153

[17] S. Pullen , J. Tessarolo , G. H. Clever , Chem. Sci. 2021, 12, 7269–7293. 34163819 10.1039/d1sc01226f PMC8171321

[18] S. Zarra , D. M. Wood , D. A. Roberts , J. R. Nitschke , Chem. Soc. Rev. 2015, 44, 419–432. 25029235 10.1039/c4cs00165f

[19] M. Hardy , A. Lützen , Chem. ‐Eur. J. 2020, 26, 13332–13346. 32297380 10.1002/chem.202001602 PMC7693062

[20] L. K. Frensch , K. Pröpper , M. John , S. Demeshko , C. Brückner , F. Meyer , Angew. Chem. Int. Ed. 2011, 50, 1420–1424. 10.1002/anie.201005780 21290526

[21] J. P. Carpenter , C. T. McTernan , T. K. Ronson , J. R. Nitschke , J. Am. Chem. Soc. 2019, 141, 11409–11413. 31282153 10.1021/jacs.9b05432 PMC6756586

[22] D. Preston , J. J. Sutton , K. C. Gordon , J. D. Crowley , Angew. Chem. Int. Ed. 2018, 57, 8659–8663. 10.1002/anie.201804745 29774643

[23] J. Guo , Y. Z. Fan , Y. L. Lu , S. P. Zheng , C. Y. Su , Angew. Chem. Int. Ed. Engl. 2020, 59, 8661–8669. 32011801 10.1002/anie.201916722

[24] H. M. O'Connor , S. Sanz , A. J. Scott , M. B. Pitak , W. T. Klooster , S. J. Coles , N. F. Chilton , E. J. L. McInnes , P. J. Lusby , H. Weihe , S. Piligkos , E. K. Brechin , Molecules 2021, 26, 757. 33540541 10.3390/molecules26030757 PMC7867156

[25] M. D. Wise , J. J. Holstein , P. Pattison , C. Besnard , E. Solari , R. Scopelliti , G. Bricogne , K. Severin , Chem. Sci. 2015, 6, 1004–1010. 29560187 10.1039/c4sc03046j PMC5811078

[26] R.‐J. Li , A. Tarzia , V. Posligua , K. E. Jelfs , N. Sanchez , A. Marcus , A. Baksi , G. H. Clever , F. Fadaei‐Tirani , K. Severin , Chem. Sci. 2022, 13, 11912–11917. 36320919 10.1039/d2sc03856k PMC9580501

[27] S. S. Mishra , S. V. K. Kompella , S. Krishnaswamy , S. Balasubramanian , D. K. Chand , Inorg. Chem. 2020, 59, 12884–12894. 32816462 10.1021/acs.inorgchem.0c01964

[28] S. Sanz , H. M. O'Connor , P. Comar , A. Baldansuren , M. B. Pitak , S. J. Coles , H. Weihe , N. F. Chilton , E. J. L. McInnes , P. J. Lusby , S. Piligkos , E. K. Brechin , Inorg. Chem. 2018, 57, 3500–3506. 29323893 10.1021/acs.inorgchem.7b02674

[29] M. Hardy , J. Tessarolo , J. J. Holstein , N. Struch , N. Wagner , R. Weisbarth , M. Engeser , J. Beck , S. Horiuchi , G. H. Clever , A. Lützen , Angew. Chem. Int. Ed. 2021, 60, 22562–22569. 10.1002/anie.202108792 PMC8519129 34382295

[30] K. Wu , E. Benchimol , A. Baksi , G. H. Clever , Nat. Chem. 2024, 16, 584–591. 38243023 10.1038/s41557-023-01415-7

[31] S. Samantray , S. Krishnaswamy , D. K. Chand , Nat. Commun. 2020, 11, 880. 32060328 10.1038/s41467-020-14703-4 PMC7021905

[32] D. Preston , J. E. Barnsley , K. C. Gordon , J. D. Crowley , J. Am. Chem. Soc. 2016, 138, 10578–10585. 27463413 10.1021/jacs.6b05629

[33] C. García‐Simón , M. Garcia‐Borràs , L. Gómez , T. Parella , S. Osuna , J. Juanhuix , I. Imaz , D. Maspoch , M. Costas , X. Ribas , Nat. Commun. 2014, 5, 5557. 25424201 10.1038/ncomms6557

[34] J. A. Findlay , K. M. Patil , M. G. Gardiner , H. I. MacDermott‐Opeskin , M. L. O'Mara , P. E. Kruger , D. Preston , Chem. Asian J. 2022, 17, e202200093. 35139260 10.1002/asia.202200093

[35] D. Preston , J. D. Evans , Angew. Chem. Int. Ed. 2023, 62, e202314378. 10.1002/anie.202314378 37816684

[36] T. Abe , N. Sanada , K. Takeuchi , A. Okazawa , S. Hiraoka , J. Am. Chem. Soc. 2023, 145, 28061–28074. 38096127 10.1021/jacs.3c09359 PMC10755705

[37] J. E. M. Lewis , A. Tarzia , A. J. P. White , K. E. Jelfs , Chem. Sci. 2020, 11, 677–683. 10.1039/c9sc05534g PMC8146399 34123040

[38] S. Tashiro , Y. Yamada , L. A. Kringe , Y. Okajima , M. Shionoya , J. Am. Chem. Soc. 2024. 10.1021/jacs.4c11583 PMC11664504 39616534

[39] R. A. S. Vasdev , D. Preston , C. A. Casey‐Stevens , V. Martí‐Centelles , P. J. Lusby , A. L. Garden , J. D. Crowley , Inorg. Chem. 2023, 62, 1833–1844. 35604785 10.1021/acs.inorgchem.2c00937

[40] T. Sawada , W. Iwasaki , M. Yamagami , M. Fujita , Nat. Sci. 2021, 1, e10008.

[41] D. F. Brightwell , K. Samanta , J. Muldoon , P. C. Fleming , Y. Ortin , L. Mardiana , P. G. Waddell , M. J. Hall , E. R. Clark , F. Fantuzzi , A. Palma , ChemistryEurope 2025, 3, e202400050.

[42] P. Molinska , A. Tarzia , L. Male , K. E. Jelfs , J. E. M. Lewis , Angew. Chem. Int. Ed. 2023, 62, e202315451. 10.1002/anie.202315451 PMC10952360 37888946

[43] X. Lu , X. Li , K. Guo , T. Z. Xie , C. N. Moorefield , C. Wesdemiotis , G. R. Newkome , J. Am. Chem. Soc. 2014, 136, 18149–18155. 25470035 10.1021/ja511341z

[44] H. Wang , X. Qian , K. Wang , M. Su , W.‐W. Haoyang , X. Jiang , R. Brzozowski , M. Wang , X. Gao , Y. Li , B. Xu , P. Eswara , X.‐Q. Hao , W. Gong , J.‐L. Hou , J. Cai , X. Li , Nat. Commun. 2018, 9, 1815. 29739936 10.1038/s41467-018-04247-z PMC5940903

[45] C. Wang , X.‐Q. Hao , M. Wang , C. Guo , B. Xu , E. N. Tan , Y.‐Y. Zhang , Y. Yu , Z.‐Y. Li , H.‐B. Yang , M.‐P. Song , X. Li , Chem. Sci. 2014, 5, 1221–1226.

[46] Z. Jiang , Y. Li , M. Wang , B. Song , K. Wang , M. Sun , D. Liu , X. Li , J. Yuan , M. Chen , Y. Guo , X. Yang , T. Zhang , C. N. Moorefield , G. R. Newkome , B. Xu , X. Li , P. Wang , Nat. Commun. 2017, 8, 15476. 28524876 10.1038/ncomms15476 PMC5454539

[47] A. J. Metherell , M. D. Ward , Chem. Commun. 2014, 50, 6330–6332. 10.1039/c4cc02627f 24799315

[48] J. Shi , K. Li , H. Yu , N. Han , T. Yang , X. Jiang , X. Q. Hao , Z. Chen , G. Wu , H. Zhang , B. Li , M. Wang , Angew. Chem. Int. Ed. Engl. 2024, 64, e202416150. 39325549 10.1002/anie.202416150

[49] J. A. Davies , T. K. Ronson , J. R. Nitschke , J. Am. Chem. Soc. 2024, 146, 5215–5223. 38349121 10.1021/jacs.3c11320 PMC10910536

[50] L. L. K. Taylor , R. Andrews , A. C. Y. Sung , I. J. Vitorica‐Yrezabal , I. A. Riddell , Chem. Commun. 2022, 58, 12301–12304. 10.1039/d2cc04624e 36254628

[51] M. Kieffer , B. S. Pilgrim , T. K. Ronson , D. A. Roberts , M. Aleksanyan , J. R. Nitschke , J. Am. Chem. Soc. 2016, 138, 6813–6821. 27145216 10.1021/jacs.6b02445

[52] W. Meng , J. K. Clegg , J. D. Thoburn , J. R. Nitschke , J. Am. Chem. Soc. 2011, 133, 13652–13660. 21790184 10.1021/ja205254s

[53] R. G. Siddique , J. J. Whittaker , H. A. Al‐Fayaad , J. C. McMurtrie , J. K. Clegg , Dalton Trans. 2023, 52, 13487–13491. 37725064 10.1039/d3dt02486e

[54] R. G. Siddique , K. S. A. Arachchige , H. A. AL‐Fayaad , J. D. Thoburn , J. C. McMurtrie , J. K. Clegg , Angew. Chem. Int. Ed. 2022, 61, e202115555. 10.1002/anie.202115555 34897921

[55] M. C. Young , L. R. Holloway , A. M. Johnson , R. J. Hooley , Angew. Chem Int. Ed. 2014, 53, 9832–9836. 10.1002/anie.201405242 25044629

[56] Edge capped Pd(II)‐based tetrahedra with two double walls are well known and naturally possess lower symmetry see; D. K. Chand , K. Biradha , M. Kawano , S. Sakamoto , K. Yamaguchi , M. Fujita , Chem. Asian J. 2006, 1, 82–90. 17441041 10.1002/asia.200600029

[57] M. R. Black , S. Bhattacharyya , S. P. Argent , B. S. Pilgrim , J. Am. Chem. Soc. 2024, 146, 28233–28241. 39236092 10.1021/jacs.4c08591 PMC11487605

[58] A. D. W. Kennedy , R. G. DiNardi , L. L. Fillbrook , W. A. Donald , J. E. Beves , Chem. ‐Eur. J. 2022, 28, e202104461. 35102616 10.1002/chem.202104461 PMC9302685

[59] Z. Wu , K. Zhou , A. V. Ivanov , M. Yusobov , F. Verpoort , Coord. Chem. Rev. 2017, 353, 180–200.

[60] A. Ferguson , M. A. Squire , D. Siretanu , D. Mitcov , C. Mathoniere , R. Clerac , P. E. Kruger , Chem. Commun. 2013, 49, 1597–1599. 10.1039/c3cc00012e 23334172

[61] R. W. Saalfrank , A. Stark , K. Peters , H. G. von Schnering , Angew. Chem. Int. Ed. 1988, 27, 851–853.

[62] C. Brückner , R. E. Powers , K. N. Raymond , Angew. Chem. Int. Ed. 1998, 37, 1837–1839.

[63] J. K. Clegg , F. Li , K. A. Jolliffe , G. V. Meehan , L. F. Lindoy , Chem. Commun. 2011, 47, 6042–6044. 10.1039/c1cc11167a 21509357

[64] R. A. Bilbeisi , S. Zarra , H. L. C. Feltham , G. N. L. Jameson , J. K. Clegg , S. Brooker , J. R. Nitschke , Chemistry 2013, 19, 8058–8062. 23653320 10.1002/chem.201300805

[65] P. W. V. Butler , P. E. Kruger , J. S. Ward , Chem. Commun. 2019, 55, 10304–10307. 10.1039/c9cc05443j 31397447

[66] R. A. Vasdev , D. Preston , S. O. Scottwell , H. J. Brooks , J. D. Crowley , M. P. Schramm , Molecules 2016, 21, 1548. 27854348 10.3390/molecules21111548 PMC6273053

[67] Deposition numbers 2414211 (for **T** _ **111** _), 2414212 (for **T** _ **112** _), and 2414213 (for **T** _ **122** _) contain the supplementary crystallographic data for this paper. These data are provided free of charge by the joint Cambridge Crystallographic Data Centre and Fachinformationszentrum Karlsruhe Access Structures service.

[68] S. Grimme , C. Bannwarth , P. Shushkov , J. Chem. Theory Comput. 2017, 13, 1989–2009. 28418654 10.1021/acs.jctc.7b00118

[69] F. Weigend , R. Ahlrichs , Phys. Chem. Chem. Phys. 2005, 7, 3297–3305. 16240044 10.1039/b508541a

[70] R. Ketkaew , Y. Tantirungrotechai , P. Harding , G. Chastanet , P. Guionneau , M. Marchivie , D. J. Harding , Dalton Trans. 2021, 50, 1086–1096. 33367357 10.1039/d0dt03988h

[71] S. K. Vellas , J. E. M. Lewis , M. Shankar , A. Sagatova , J. D. A. Tyndall , B. C. Monk , C. M. Fitchett , L. R. Hanton , J. D. Crowley , Molecules 2013, 18, 6383–6407. 23760034 10.3390/molecules18066383 PMC6290563

[72] To check that the differences in stability were not due to the different strengths of the donor sites due to different substitutions of the ligand arms, the stability of **T** _ **111** _' toward DMSO was also assessed. **T** _ **111** _' was synthesized from **L** _ **111** _' (Supporting Information) which had the same equilateral geometry and direct connection between the arms and core as **L** _ **111** _ but had the 1‐(3,5‐dimethoxybenzyl) from Arm3. Despite the 1‐(3,5‐dimethoxybenzyl) arm being present on the least stable cages in the main study, **T** _ **111** _’ showed stability toward [D_6_]DMSO up to the same 25% volume as for **T** _ **111** _, indicating that the primary driver for the stability of the complexes was their global distortion away from a regular tetrahedron rather than the identity of the solubilizing group.

[73] Y.‐L. Lu , J.‐Q. Song , Y.‐H. Qin , J. Guo , Y.‐H. Huang , X.‐D. Zhang , M. Pan , C.‐Y. Su , J. Am. Chem. Soc. 2022, 144, 8778–8788. 35507479 10.1021/jacs.2c02692

[74] J. J. Stone , R. A. Stockland , N. P. Rath , Inorg. Chim. Acta 2003, 342, 236–240.

[75] M. Juríček , M. Felici , P. Contreras‐Carballada , J. Lauko , S. R. Bou , P. H. J. Kouwer , A. M. Brouwer , A. E. Rowan , J. Mater. Chem. 2011, 21, 2104–2111.

[76] D. Preston , A. Fox‐Charles , W. K. Lo , J. D. Crowley , Chem. Commun. 2015, 51, 9042–9045. 10.1039/c5cc02226f 25940817

[77] D. Aragao , J. Aishima , H. Cherukuvada , R. Clarken , M. Clift , N. P. Cowieson , D. J. Ericsson , C. L. Gee , S. Macedo , N. Mudie , S. Panjikar , J. R. Price , A. Riboldi‐Tunnicliffe , R. Rostan , R. Williamson , T. T. Caradoc‐Davies , J. Synchrotron Radiat. 2018, 25, 885–891. 29714201 10.1107/S1600577518003120 PMC5929359

[78] W. Kabsch , J. Appl. Crystallogr. 1993, 26, 795–800.

[79] G. M. Sheldrick , Acta Crystallogr. Sect. A: Found. Crystallogr. 2008, 64, 112–122. 10.1107/S0108767307043930 18156677

[80] O. V. Dolomanov , L. J. Bourhis , R. J. Gildea , J. A. K. Howard , H. Puschmann , J. Appl. Crystallogr. 2009, 42, 339–341. 10.1107/S0021889811041161 PMC3236671 22199401

[81] F. Neese , WIREs Comp. Mol. Sci. 2022, 12, e1606.

[82] S. Grimme , A. Hansen , S. Ehlert , J.‐M. Mewes , J. Chem. Phys. 2021, 154, 064103. 33588555 10.1063/5.0040021

[83] P. Pollak , F. Weigend , J. Chem.l Theory Comput. 2017, 13, 3696–3705. 10.1021/acs.jctc.7b00593 28679044

