# Supplementary material for: Low‐Symmetry Face‐Capped Fe(II) Tetrahedra Through Anisotropic Ligand Extension
Source: Angew Chem Int Ed Engl. 2025 Mar 16;64(21):e202503473. doi: 10.1002/anie.202503473 (PMC12087859; doi:10.1002/anie.202503473)
Supplement: Supplementary file 1 — Supporting information [file ANIE-64-e202503473-s001.pdf]

Supporting Information

Jacob A. Gome,[a] Zack T. Avery,[a] Nina R. Lawson,[a] Oliver G. Stansfield,[b] Jack D. Evans,[c] Michael G. Gardiner,[a] Timothy U. Connell,[b] and Dan Preston[a],*

[a] J.A. Gome, Z.T. Avery, N.R. Lawson, Dr. M.G. Gardiner, Dr. D. Preston
Research School of Chemistry
Australian National University
Canberra 2601, ACT, Australia
E-mail: daniel.preston@anu.edu.au

[b] O.G. Stansfield, Dr. T.U. Connell
Centre for Sustainable Bioproducts, Faculty of Science, Engineering and Built Environment
Deakin University,
Geelong, Victoria 3220, Australia

[c] Dr. J.D. Evans
School of Physics, Chemistry and Earth Sciences
The University of Adelaide
Adelaide, SA 5005, Australia

Contents

1. Experimental **3**

1.1. General **3**

1.2. Precursors **4**

1.2.1. 5-Bromo-2-(1-(2-methoxyethyl)-1*H*-1,2,3-triazol-4-yl)pyridine (2) 4

1.2.2. 2-(1-(2-Methoxyethyl)-1H-1,2,3-triazol-4-yl)-5-(4,4,5,5-tetramethyl-1,3,2-dioxaborolan-2-yl)pyridine (3) 5

1.2.3. 5-Bromo-2-(1-(2,2-dimethoxyethyl)-1*H*-1,2,3-triazol-4-yl)pyridine (4) 5

1.2.4. 2-(1-(2,2-Dimethoxyethyl)-1H-1,2,3-triazol-4-yl)-5 ((trimethylsilyl)ethynyl)pyridine (5) . 6

1.2.5. 5-Bromo-2-(1-(3,5-dimethoxybenzyl)-1H-1,2,3-triazol-4-yl)pyridine (6) 7

1.2.6. 2-(1-(3,5-Dimethoxybenzyl)-1H-1,2,3-triazol-4-yl)-5-((trimethylsilyl)ethynyl)pyridine (7) 8

1.2.7. 2-(1-(3,5-dimethoxybenzyl)-1H-1,2,3-triazol-4-yl)-5-((triisopropylsilyl)buta-1,3-diyn-1-yl)pyridine (8) 9

1.2.8. 5,5'-(5-Iodo-1,3-phenylene)bis(2-(1-(2-methoxyethyl)-1H-1,2,3-triazol-4-yl)pyridine) (9) 11

1.2.9. 5-(3,5-Diiodophenyl)-2-(1-(2-methoxyethyl)-1H-1,2,3-triazol-4-yl)pyridine (10) 12

1.2.10. 5-((3,5-diiodophenyl)buta-1,3-diyn-1-yl)-2-(1-(3,5-dimethoxybenzyl)-1H-1,2,3-triazol-4-yl)pyridine (11) 13

1.2.11. 2-(1-(3,5-Dimethoxybenzyl)-1H-1,2,3-triazol-4-yl)-5-((3-((6-(1-(2,2-dimethoxyethyl)-1H-1,2,3-triazol-4-yl)pyridin-3-yl)ethynyl)-5-iodophenyl)buta-1,3-diyn-1-yl)pyridine (12) 14

1.3. Ligands **16**

1.3.1. 1,3,5-tris(6-(1-(2-methoxyethyl)-1H-1,2,3-triazol-4-yl)pyridin-3-yl)benzene (L_{111}) 16

1.3.2. 5,5'-(5-((6-(1-(2,2-dimethoxyethyl)-1H-1,2,3-triazol-4-yl)pyridin-3-yl)ethynyl)-1,3-phenylene)bis(2-(1-(2-methoxyethyl)-1H-1,2,3-triazol-4-yl)pyridine) (L_{112}) 17

1.3.3. 5,5'-((5-(6-(1-(2-methoxyethyl)-1H-1,2,3-triazol-4-yl)pyridin-3-yl)-1,3-phenylene)bis(ethyne-2,1-diyl))bis(2-(1-(2,2-dimethoxyethyl)-1H-1,2,3-triazol-4-yl)pyridine) (L_{122}) 19

1.3.4. 5,5'-(5-((6-(1-(3,5-dimethoxybenzyl)-1H-1,2,3-triazol-4-yl)pyridin-3-yl)buta-1,3-diyn-1-yl)-1,3-phenylene)bis(2-(1-(2-methoxyethyl)-1H-1,2,3-triazol-4-yl)pyridine) (L_{113}) 21

1.3.5. 2-(1-(3,5-dimethoxybenzyl)-1H-1,2,3-triazol-4-yl)-5-((3-((6-(1-(2,2-dimethoxyethyl)-1H-1,2,3-triazol-4-yl)pyridin-3-yl)ethynyl)-5-(6-(1-(2-methoxyethyl)-1H-1,2,3-triazol-4-yl)pyridin-3-yl)phenyl)buta-1,3-diyn-1-yl)pyridine (L_{123}) 23

1.3.6. 1,3,5-tris(6-(1-(3,5-dimethoxybenzyl)-1H-1,2,3-triazol-4-yl)pyridin-3-yl)benzene (L_{111}‘) 25

1.4. Complexes **26**

1.4.1. Tet_{111} 26

1.4.2. Tet_{112} 30
1.4.3. Tet_{122} 36
1.4.4. Tet_{113} 42
1.4.5. Tet_{123} 46
1.4.6. Tet_{111}' 51
2. Computations 53
2.1. GFN-xtb 53
2.1.1. Comparison between GFN-xtb model and X-ray structure of T_{111} 53
2.1.2. Comparison between GFN-xtb model and X-ray structure of T_{112} 54
2.1.3. Comparison between GFN-xtb model and X-ray structure of T_{122} 54
2.1.4. Comparison between GFN-xtb model and DFT structure of T_{123} (MapBΛ_4) 55
2.2. DFT calculations 55
3. Structure relationship studies 55
3.1. Calculating structural distortion 55
3.2. DMSO stability studies 57
3.3. Electrochemistry 58
4. References 59

1. Experimental

1.1. General

Unless otherwise stated, all reagents were purchased from commercial sources and used without further purification, except for: $Pd(PPh_3)_2Cl_2$[1], 5-bromo-2-((trimethylsilyl)ethynyl)pyridine[2] and 3-[2-(2-Methoxyethoxy)ethoxy]-5-[2-(trimethylsilyl)ethynyl]pyridine[3] which were synthesised according to literature procedures. Solvents were laboratory reagent grade. Petroleum ether (PE) refers to the fraction of petrol boiling in the range 40 – 60 °C, dichloromethane (DCM), ethylenediaminetetraacetate (EDTA), tetrahydrofuran (THF), dimethyl sulfoxide (DMSO), dimethylformamide (DMF), ethyl acetate (EtOAc). ^1H and ^{13}C NMR spectra were recorded on either a Bruker Avance 400 MHz or a Bruker Avance 800 MHz spectrometer. Chemical shifts are reported in parts per million and referenced to residual solvent peaks ($CDCl_3$: ^1H δ 7.26 ppm, ^{13}C δ 77.16 ppm; d_6- DMSO: ^1H δ 2.50 ppm; ^{13}C δ 39.52 ppm; CD_3CN: ^1H δ 1.94 ppm; ^{13}C δ 1.32 & 118.26 ppm). Coupling constants (*J*) are reported in Hertz (Hz). Standard abbreviations indicating multiplicity were used as follows: m = multiplet, q = quartet, quin = quintet, t = triplet, dt = double triplet, d = doublet, dd = double doublet, s = singlet, br = broad. Electrospray mass spectra (HR ESI-MS) and Nanospray MS were collected on an Orbitrap Elite spectrometer or a Waters Synapt G2-S1 HDMS spectrometer.

CAUTION: WHILE NO PROBLEMS WERE ENCOUNTERED DURING THIS WORK, AZIDES ARE EXPLOSIVE AND CARE SHOULD BE TAKEN WHEN DEALING WITH THEM.

1.2. Precursors

1.2.1. 5-Bromo-2-(1-(2-methoxyethyl)-1*H*-1,2,3-triazol-4-yl)pyridine (2)

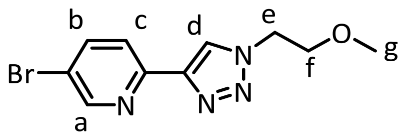

Sodium azide (235 mg, 3.61 mmol, 1.1 eq) and 1-iodo-2-methoxyethane (600 mg, 3.22 mmol, 1.0 eq) were stirred in an open flask in DMF (20 mL) at 110 °C for 2 hrs. The flask was allowed to cool before 5-bromo-2-((trimethylsilyl)ethynyl)pyridine (810 mg, 3.19 mmol, 1.0 eq) and Na_2CO_3 (525 mg, 4.95 mmol, 1.5 eq) were added with stirring. After 10 minutes, $CuSO_4{\cdot}5H_2O$ (402 mg, 1.61 mmol, 0.50 eq) and sodium ascorbate (640 mg, 3.22 mmol, 1.0 eq) were added with 2 mL of water. The solution was stirred overnight at room temperature under a protective layer of N_2. DCM (200 mL) and 0.1 M EDTA/$NH_4OH_{(aq)}$ (200 mL) were added. The organic layer was extracted and washed with water (6 × 100 mL) before being concentrated. This was purified by flash column chromatography on silica eluting with a gradient of DCM to DCM/EtOAc 1:1 (v/v). Product was obtained as an off-white powder (734 mg, 80%).

^{1}H NMR (400 MHz, $CDCl_3$, 298 K) *δ*: 8.60 (br s, 1H, H_a), 8.22 (s, 1H, H_d), 8.05 (br s, 1H, H_b), 7.86 (d, *J* = 8.3 Hz, 1H, H_c), 4.56 (t, *J* = 5.1 Hz, 2H, H_e), 3.76 (t, *J* = 5.1 Hz, 2H, H_f), 3.33 (s, 3H, H_g);
^{13}C NMR (100 MHz, $CDCl_3$, 298 K) *δ*: 150.3, 148.8, 147.3, 139.4, 123.3, 121.4, 119.5 70.6, 59.0, 50.5;
HR ESI-MS (DCM/MeOH) *m/z* = 305.0024 $[M+Na]^+$ (calc. for $C_{10}H_{11}{}^{81}BrN_4ONa^+$, 305.0014);
IR *ν* (cm^{-1}): 3150, 2998, 2916, 2765, 1225, 1929, 1867, 1664, 1594, 1459, 1434, 1361, 1227, 1109, 819

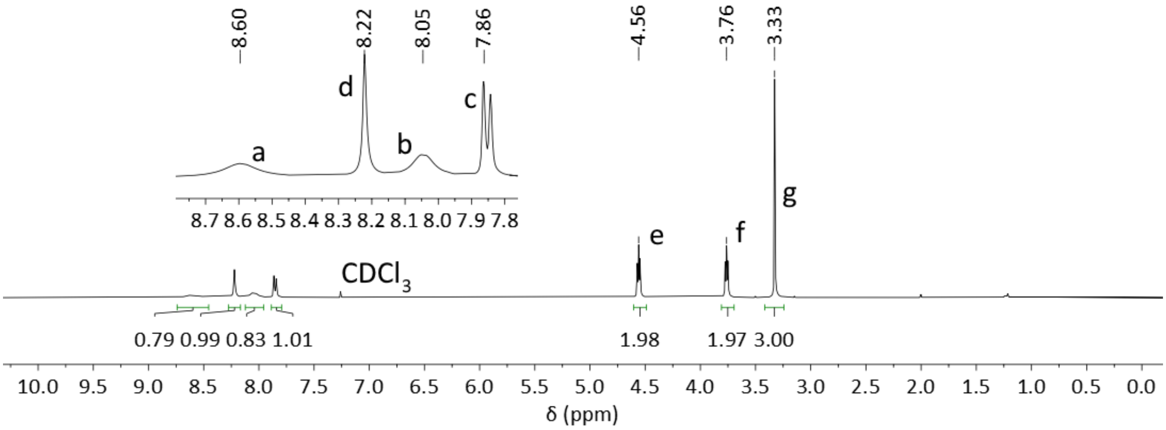

Figure S1.1 ^{1}H NMR spectrum of **2** (400 MHz, $CDCl_3$, 298 K).

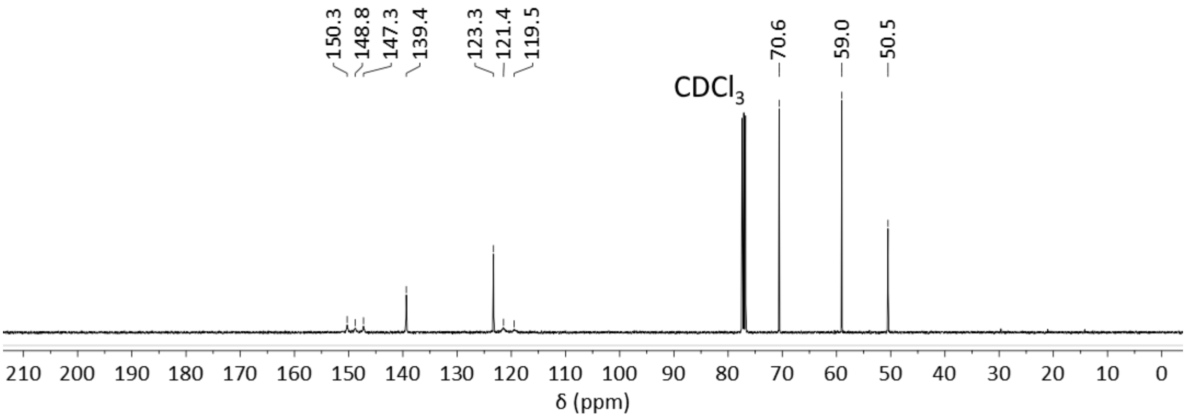

Figure S1.2 ^{13}C NMR spectrum of **2** (100 MHz, $CDCl_3$, 298 K).

1.2.2. 2-(1-(2-Methoxyethyl)-1H-1,2,3-triazol-4-yl)-5-(4,4,5,5-tetramethyl-1,3,2-dioxaborolan-2-yl)pyridine (3)

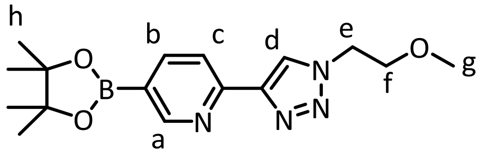

Bis(pinacolato)diboron (296 mg, 1.17 mmol, 1.1 eq), **2** (300 mg, 1.06 mmol, 1.0 eq) and KOAc (312 mg, 3.18 mmol, 3.0 eq) were stirred in dioxane (10 mL). The resulting mixture was degassed with N_2 for 1 hr. 1,1′-Bis(diphenylphosphino)ferrocene (29.4 mg, 0.0530 mmol, 0.0050 eq) and [Pd(CH_3CN)$_2$$Cl_2$] (15.6 mg, 0.0601 mmol, 0.0050 eq) were added under an N_2 atmosphere and the resulting solution was stirred at 85 °C for 48 hrs. DCM (50 mL) and water (50 mL) were added. The organic layer was extracted and washed with water (50 mL) before being concentrated. The crude product (343 mg, 80% by ^{1}H NMR) was characterised by ^{1}H NMR and HR ESI-MS and used as is in subsequent reactions.

^{1}H NMR (400 MHz, $CDCl_3$, 298 K) δ: 8.89 (br s, 1H, H_a), 8.32 (br s, 1H, H_d), 8.14 (br s, 2H, $H_{b,c}$), 4.58 (t, J = 4.5 Hz, 2H, H_e), 3.78 (t, J = 4.5 Hz, 2H, H_f), 3.35 (s, 3H, H_g), 1.35 (s, 12H, H_h);
HR ESI-MS (DCM/MeOH) m/z = 353.1773 [M+Na]$^+$ (calc. for $C_{16}H_{23}BN_4O_3Na^+$, 353.1771)

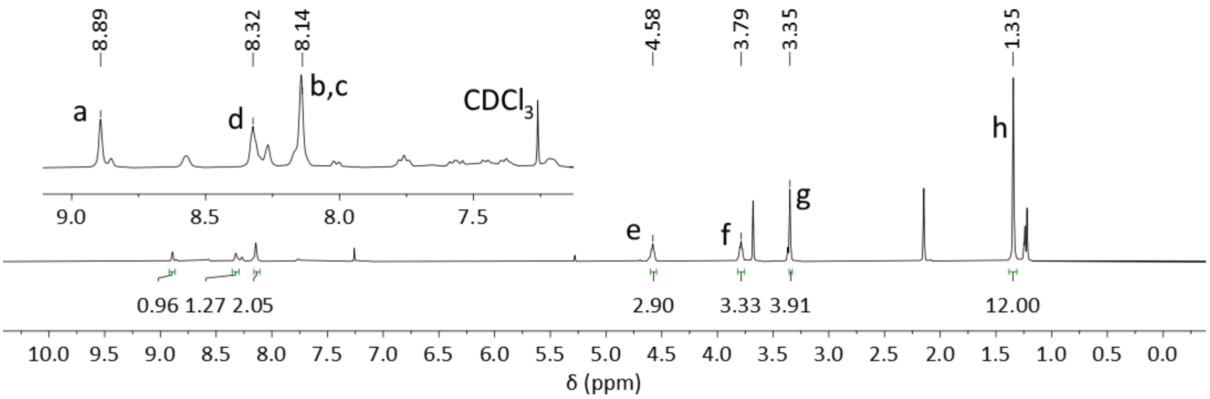

Figure S1.3: ^{1}H NMR spectrum of Crude **3** (400 MHz, $CDCl_3$, 298 K).

1.2.3. 5-Bromo-2-(1-(2,2-dimethoxyethyl)-1*H*-1,2,3-triazol-4-yl)pyridine (4)

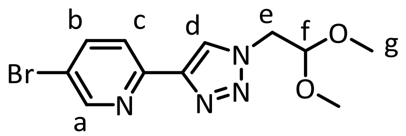

Sodium azide (64 mg, 0.98 mmol, 1.1 eq) and 1-bromo-2,2-dimethoxyethane (150 mg, 0.887 mmol, 1.0 eq) were stirred in an open flask in DMF (7 mL) at 110 °C for 2 hrs. The flask was allowed to cool before 5-bromo-2-((trimethylsilyl)ethynyl)pyridine (226 mg, 0.889 mmol, 1.0 eq) and Na_2CO_3 (145 mg, 1.37 mmol, 1.5 eq) were added with stirring. After 10 minutes, $CuSO_4 \cdot 5H_2O$ (90 mg, 0.36 mmol, 0.40 eq) and sodium ascorbate (176 mg, 0.877 mmol, 1.0 eq) were added with 1 mL of water. The solution was stirred overnight at room temperature under a protective layer of N_2. DCM (75 mL) and 0.1 M EDTA/$NH_4OH_{(aq)}$ (75 mL) were added. The organic layer was extracted and washed with water (6 × 75 mL) before being concentrated. This was purified by flash column chromatography on silica eluting with a gradient of DCM to DCM/EtOAc 1:1 (v/v). Product was obtained as off-white crystals (152 mg, 55%).

^{1}H NMR (400 MHz, d_6-DMSO, 298 K) *δ*: 8.71 (br s, 1H, H_a), 8.57 (s, 1H, H_d), 8.11 (d, *J* = 8.4 Hz 1H, H_b), 7.96 (d, *J* = 8.4 Hz, 1H, H_c), 4.81 (t, *J* = 5.0 Hz, 1H, H_f), 4.59 (d, *J* = 5.0 Hz, 2H, H_e), 3.31 (s, 6H, H_g);
^{13}C NMR (100 MHz, d_6-DMSO, 298 K) *δ*: 150.7, 149.1, 146.6, 140.3, 124.7, 121.5, 119.3, 101.9, 54.3, 51.2;
HR ESI-MS (DCM/MeOH) *m/z* = 337.0107 $[M+Na]^+$ (calc. for $C_{11}H_{13}{}^{81}BrN_4O_2Na^+$, 337.0100);
IR *v* (cm^{-1}): 3391, 2958, 2924, 2854, 2260, 2131, 1651, 1460, 991.

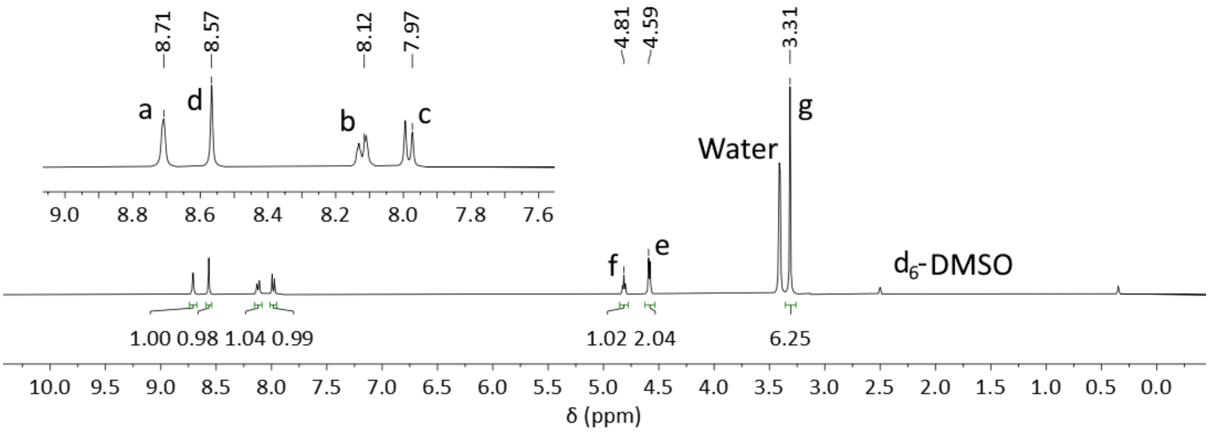

Figure S1.4 ^{1}H NMR spectrum of **4** (400 MHz, d_6-DMSO, 298 K).

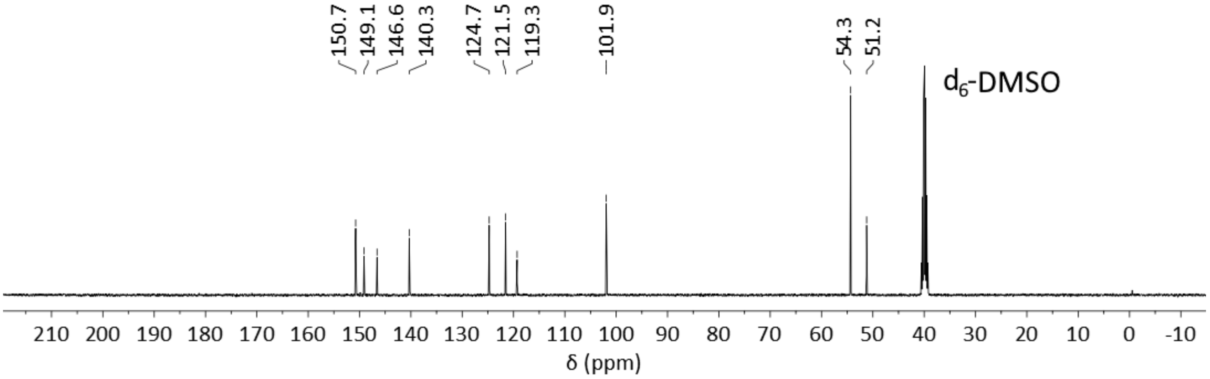

Figure S1.5: ^{13}C NMR spectrum of **4** (100 MHz, d_6-DMSO, 298 K).

1.2.4. 2-(1-(2,2-Dimethoxyethyl)-1H-1,2,3-triazol-4-yl)-5((trimethylsilyl)ethynyl)pyridine (5)

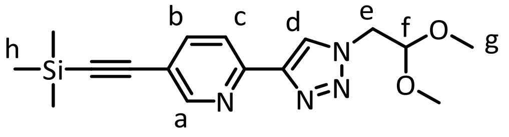

Compound **4** (500 mg, 1.60 mmol, 1.0 eq) and TMS-acetylene (250 µL, 1.76 mmol, 1.1 eq) were dissolved in a degassed mixture of THF (5 mL), TEA (5 mL). Copper (I) iodide (30 mg, 0.16 mmol, 0.10 eq) and $[Pd(PPh_3)_2Cl_2]$ (56 mg, 0.080 mmol, 0.050 eq) were added and the solution was stirred under a protective layer of N_2 overnight at 50 °C. 0.1M EDTA/$NH_4OH_{(aq)}$ (50 mL) was added and the organic layer was extracted in DCM (3 × 50 mL). The organic layers were combined and washed with brine (100 mL), and then concentrated to give a crude product. This was purified by flash column chromatography on silica eluting with a gradient of DCM to DCM/acetone 1:0.05 (v/v). Product was obtained as off-white crystals (244 mg, 46%).

^{1}H NMR (400 MHz, $CDCl_3$, 298 K) *δ*: 8.61 (d, *J* = 1.4 Hz, 1H, H_a), 8.20 (s, 1H, H_d), 8.07 (d, *J* = 8.2 Hz, 1H, H_c), 7.80 (dd, *J* = 8.1, 2.2 Hz, 1H, H_b), 4.67 (t, *J* = 5.2 Hz, 1H, H_f), 4.48 (d, *J* = 5.2 Hz, 2H H_e), 3.39 (s, 3H, H_g), 0.24 (s, 9H, H_h);
^{13}C NMR (100 MHz, $CDCl_3$, 298 K) *δ*: 152.4, 149.3, 148.0, 139.9, 123.9, 119.5, 119.3, 102.6, 101.9, 98.7, 55.1, 52.2, 0.3;
HR ESI-MS (DCM/MeOH) *m/z* = 331.1596 $[M+H]^+$ (calc. for $C_{16}H_{22}N_4O_2SiH^+$, 331.1590); *m/z* = 353.1447 $[M+Na]^+$ (calc. for $C_{16}H_{22}N_4O_2SiNa^+$, 353.1410);
IR *v* (cm^{-1}): 2996, 2959, 2900, 2836, 2158, 1595, 1461, 1364, 1250, 1125, 1075, 840

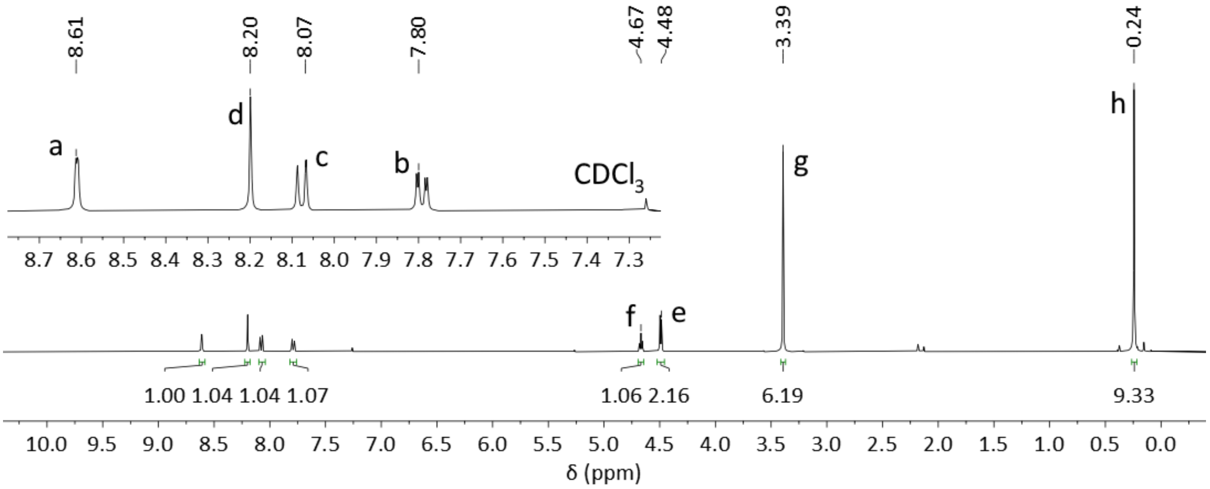

Figure S1.6: ^{1}H NMR Spectrum of **5** (400 MHz, $CDCl_3$, 298 K).

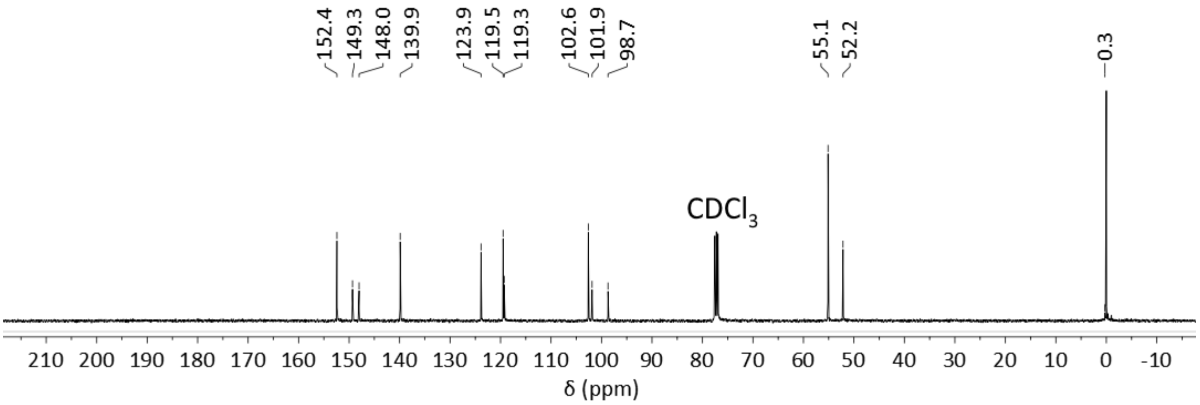

Figure S1.7: ^{13}C NMR Spectrum of **5** (100 MHz, $CDCl_3$, 298 K).

1.2.5. 5-Bromo-2-(1-(3,5-dimethoxybenzyl)-1H-1,2,3-triazol-4-yl)pyridine (6)

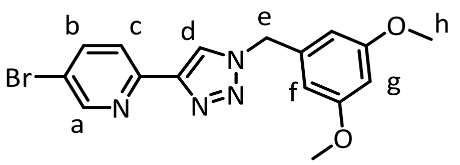

Sodium azide (77 mg, 1.2 mmol, 1.1 eq) and 3,5-dimethoxybenzylbromide (250 mg, 1.08 mmol, 1.0 eq) stirred in an open flask in DMF (7 mL) at 80 °C for 2 hrs. The flask was allowed to cool before 5-bromo-((trimethylsilyl)ethynyl)pyridine (275 mg, 1.08 mmol, 1.0 eq) and Na_2CO_3 (172 mg, 1.62 mmol, 1.5 eq) were added with stirring. After 10 minutes, $CuSO_4{\cdot}5H_2O$ (135 mg, 0.541 mmol, 0.50 eq) and sodium ascorbate (214 mg, 1.08 mmol, 1.0 eq) were added with 1 mL of water. The solution was stirred overnight at room temperature under a protective layer of N_2. DCM (75 mL) and

0.1 M EDTA/$NH_4OH_{(aq)}$ (75 mL) were added. The organic layer was extracted and washed with water (6 × 75 mL) before being concentrated. This was purified by flash column chromatography on silica eluting with a gradient of DCM to DCM/acetone 1:0.05 (v/v). Product was obtained as off-white crystals (232 mg, 58%).

^{1}H NMR (400 MHz, $CDCl_3$, 298 K) δ: 8.52 (d, J = 2.2 Hz, 1H, H_a), 8.02 (s, 1H, H_d), 7.99 (d, J = 8.5 Hz, 1H, H_c), 8.11 (dd, J = 8.5, 2.2 Hz, 1H, H_b), 6.40 (d, J = 2.2 Hz, 2H, H_f), 6.38 (t, J = 2.2 Hz, 1H, H_g), 5.44 (s, 2H, H_e), 3.70 (s, 6H, H_h);
^{13}C NMR (100 MHz, $CDCl_3$, 298 K) δ: 161.3, 150.3, 148.7, 147.7, 139.4, 136.3, 122.3, 121.3, 119.4, 106.3, 100.5, 55.4, 54.4;
HR ESI-MS (DCM/MeOH) m/z = 397.0285 $[M+Na]^+$ (calc. for $C_{16}H_{15}{}^{79}BrN_4O_2Na^+$, 397.0276);
IR ν (cm^{-1}): 3149, 3006, 2968, 2938, 2834, 1596, 1459, 1361, 1206, 1160, 1067

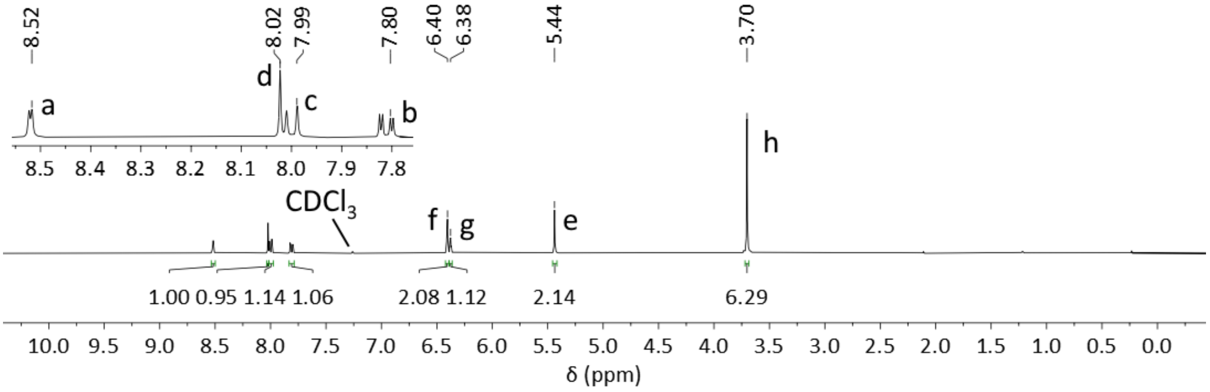

Figure S1.8: ^{1}H NMR spectrum of **6** (400 MHz, $CDCl_3$, 298 K).

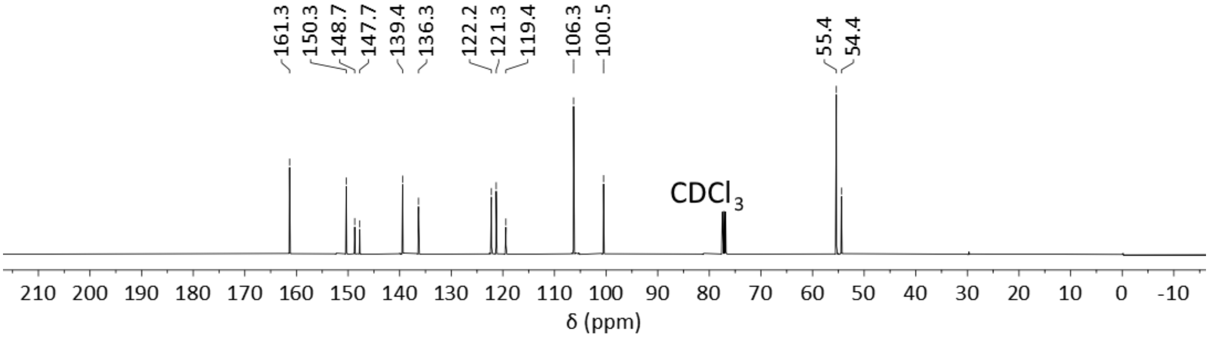

Figure S1.9: ^{13}C NMR spectrum of **6** (100 MHz, $CDCl_3$, 298 K).

1.2.6. 2-(1-(3,5-Dimethoxybenzyl)-1H-1,2,3-triazol-4-yl)-5-((trimethylsilyl)ethynyl)pyridine (7)

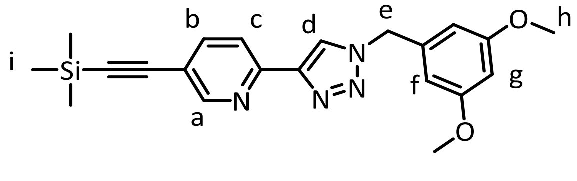

Compound **6** (500 mg, 1.33 mmol, 1.0 eq) and TMS-acetylene (380 µL, 2.67 mmol, 2 eq) were dissolved in a degassed mixture of dioxane (1.5 mL) and TEA (1.5 mL). Copper (I) iodide (25 mg, 0.13 mmol, 0.10 eq) and $[Pd(PPh_3)_2Cl_2]$ (70 mg, 0.10 mmol, 0.075 eq) were added and the solution was heated in a sealed tube at 80 °C overnight. 0.1M EDTA/$NH_4OH_{(aq)}$ (15 mL) was added and the organic layer was extracted in DCM (3 × 20 mL). The organic layers were combined and washed with brine (60 mL), and

then concentrated to give a crude product. This was purified by flash column chromatography on silica eluting with a gradient of DCM to DCM/acetone 1:0.05 (v/v). Product was obtained as off-white crystals (422 mg, 81%).

^{1}H NMR (400 MHz, $CDCl_3$, 298 K) δ: 9.40 (d, J = 1.2 Hz, 1H, H_a), 8.93 (d, J = 8.0 Hz, 1H, H_c), 8.89, (s, 1H, H_d), 8.63 (dd, J = 8.2, 1.9 Hz, 1H, H_b), 7.25 (d, J = 2.1 Hz, 2H, H_f), 7.23 (t, J = 2.1 Hz, 1H, H_g), 6.29 (s, 2H, H_e), 4.56 (s, 6H, H_h), 1.07 (s, 9H, H_i);
^{13}C NMR (100 MHz, $CDCl_3$, 298 K) δ: 161.5, 152.2, 149.2, 148.2, 140.0, 136.4, 122.7, 119.6, 106.5, 101.8, 100.8, 98.9, 55.6, 54.7, 0.0;
HR ESI-MS (DCM/MeOH) m/z = 393.1743 $[M+H]^+$ (calc. for $C_{21}H_{25}N_4O_2Si$, 393.1741); m/z = 415.1564 $[M+Na]^+$ (calc. for $C_{21}H_{24}N_4O_2SiNa$, 415.1561);
IR ν (cm^{-1}) 3160, 3005, 2960, 2840, 2156, 1593, 1472, 1430, 1349, 1206, 1153, 1067, 838

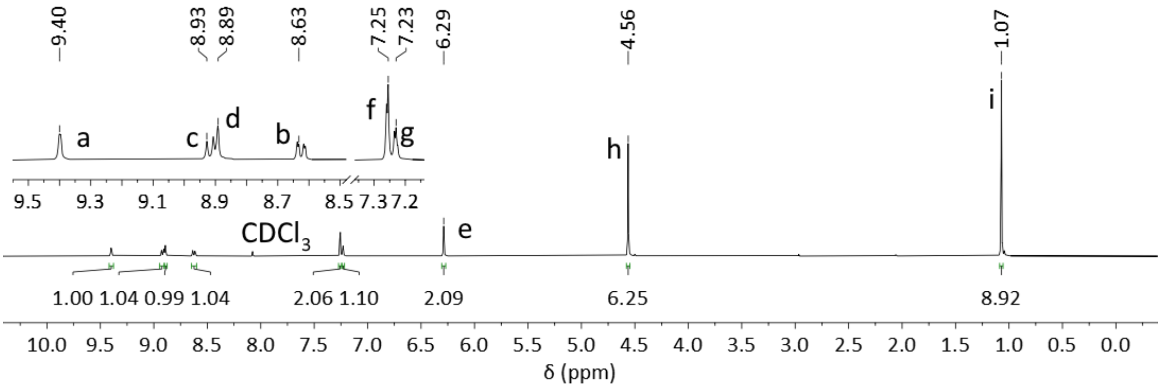

Figure S1.10: ^{1}H NMR spectrum of **7** (400 MHz, $CDCl_3$, 298 K).

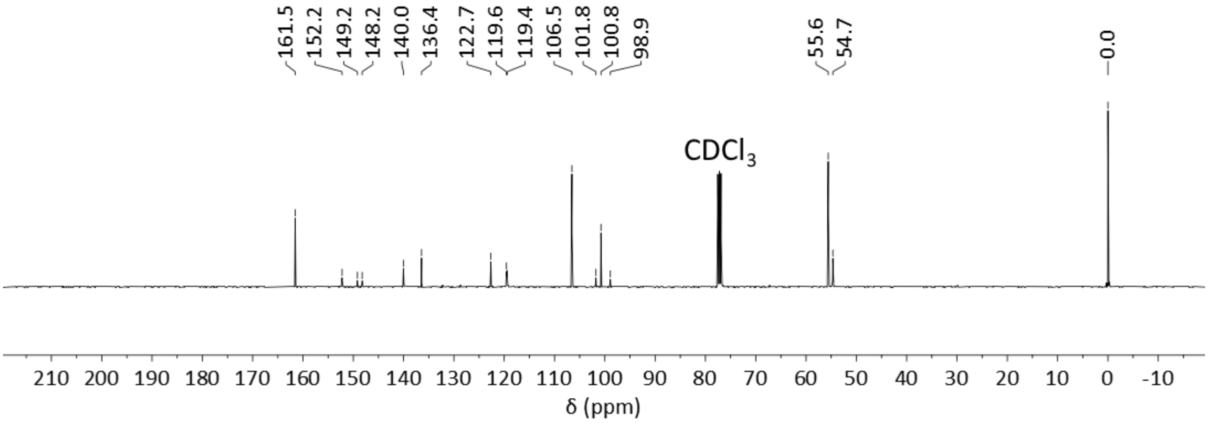

Figure S1.11: ^{13}C NMR spectrum of **7** (100 MHz, $CDCl_3$, 298 K).

1.2.7. 2-(1-(3,5-dimethoxybenzyl)-1H-1,2,3-triazol-4-yl)-5-((triisopropylsilyl)buta-1,3-diyn-1-yl)pyridine (8)

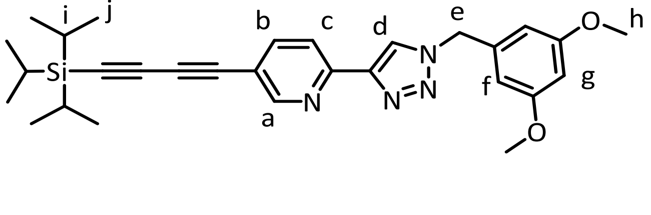

Compound **7** (1.20 g, 3.06 mmol, 1.0 eq) and Na_2CO_3 (654 mg, 6.17 mmol, 2.0 eq) were added to MeOH (30 mL) and stirred for 2 hours. The solution was filtered to remove solid and concentrated. The concentrate was dissolved in 25 mL EtOH and the solution was degassed for 1.5 hrs. Copper (I) bromide (43 mg, 0.30 mmol, 0.10 eq), sodium ascorbate (606 mg, 3.06 mmol, 1.0 eq), *n*-butylamine (380 µL, 3.84 mmol, 1.3 eq) and bromo-triisopropylethylene (1.63 mL, 6.17 mmol, 2.0 eq) were added. The reaction mixture was heated at 50 °C overnight and then concentrated. This was purified by flash column chromatography on silica eluting with a gradient of DCM to DCM/acetone 1:0.05 (v/v). Product was obtained as off-white crystals (922 mg, 72%).

^{1}H NMR (400 MHz, $CDCl_3$, 298 K) δ: 8.63 (br s, 1H, H_a), 8.13 (d, J = 8.1 Hz, 1H, H_c), 8.07 (s, 1H, H_d), 7.83 (dd, J = 8.2, 2.0 Hz, 1H, H_b), 6.44 (d, J = 2.2 Hz, 2H, H_f), 6.42 (t, J = 2.2 Hz, 1H, H_g), 3.75 (s, 6H, H_h), 1.11 (s, 18H, H_i), 1.10 (s, 3H, H_j);
^{13}C NMR (100 MHz, $CDCl_3$, 298 K) δ: 161.4, 152.4, 149.2, 147.5, 140.8, 136.15, 122.94, 119.66, 117.96, 106.4, 100.7, 90.3, 78.5, 72.0, 55.4, 54.6, 18.6, 11.3;
HR ESI-MS (DCM/MeOH) *m/z* = 501.2677 $[M+H]^+$ (calc. for $C_{29}H_{37}N_4O_2Si$, 501.2680); *m/z* = 523.2500 $[M+Na]^+$ (calc. for $C_{29}H_{36}N_4O_2SiNa$, 523.2500);
IR v (cm^{-1}) 2942, 2880, 2865, 2203, 1202, 1594, 1461, 1430, 1354, 1206, 1157, 1067, 1041, 882

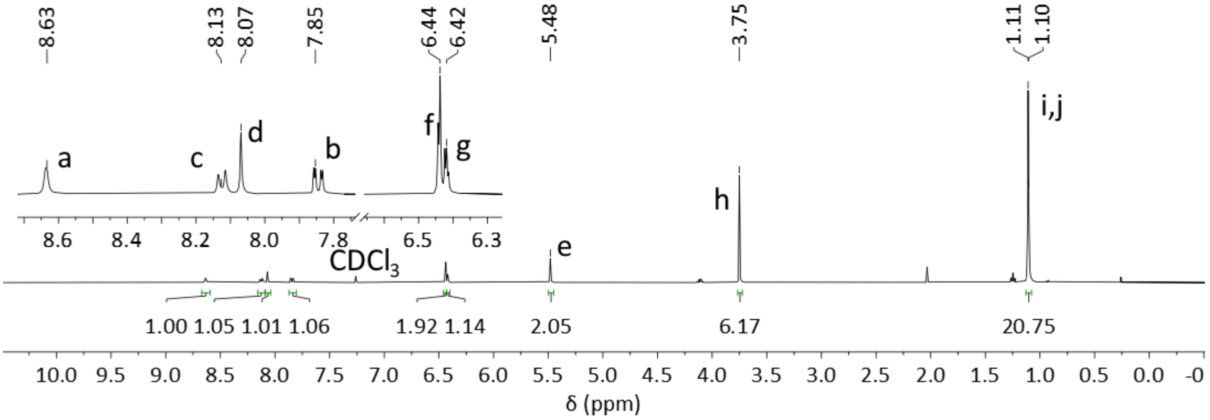

Figure S1.12: ^{1}H NMR spectrum of **8** (400 MHz, $CDCl_3$, 298 K).

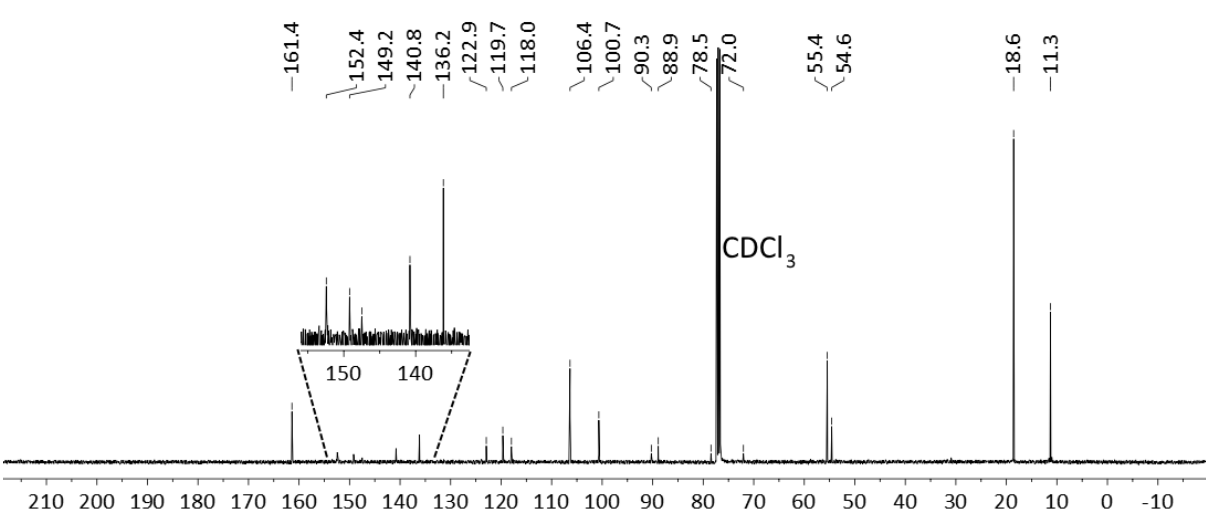

Figure S1.13: ^{13}C NMR spectrum of **8** (100 MHz, $CDCl_3$, 298 K).

1.2.8. 5,5'-(5-Iodo-1,3-phenylene)bis(2-(1-(2-methoxyethyl)-1H-1,2,3-triazol-4-yl)pyridine) (9)

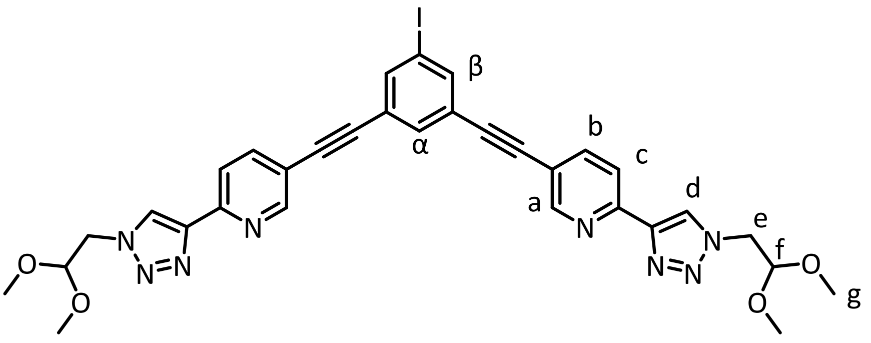

Compound **5** (196 mg, 0.593 mmol, 1.5 eq) and Na_2CO_3 (128 mg, 1.21 mmol, 3.0 eq) were stirred in MeOH (5 mL) in a flask open to the air for 2 hours. The solution was decanted and concentrated. The resulting solid was added to a solution of 1,3,5-triiodobenzene (184 mg, 0.403 mmol, 1.0 eq) in THF (3 mL) and TEA (3 mL). The solution was degassed for 2 hr before copper (I) iodide (7.7 mg, 0.040 mmol, 0.10 eq), [Pd_2(dibenzylideneacetone)$_3$] (9.2 mg, 0.010 mmol, 0.025 eq) and triphenylphosphine (10.6 mg, 0.0403 mmol, 0.10 eq) were added. The resulting solution was stirred under a protective layer of N_2 overnight at 50 °C. 0.1M EDTA/$NH_4OH_{(aq)}$ (25 mL) was added and the organic layer was extracted in DCM (3 × 25 mL). The organic layers were combined and washed with brine (75 mL), and then concentrated to give a crude product. This was purified by flash column chromatography on silica eluting with a gradient of DCM to DCM/acetone 1:0.05 (v/v). Product was obtained as yellow crystals (95 mg, 33%).

^{1}H NMR (400 MHz, $CDCl_3$, 298 K) δ: 8.71 (d, J = 1.2 Hz, 2H, H_a), 8.25 (s, 2H, H_d), 8.17 (d, J = 8.3 Hz, 2H, H_c), 7.89 (d, J = 1.2 Hz, 2H, H_β) 7.86 (dd, J = 8.2, 2.1 Hz, 2H, H_b), 7.70 (t, J = 1.2 Hz, 1H, H_α), 4.70 (t, J = 5.3 Hz, 2H, H_f), 4.54 (d, J = 5.3 Hz, 2H, H_e), 3.43 (s, 12 H, H_g);
^{13}C NMR (100 MHz, $CDCl_3$, 298 K) δ: 152.0, 149.5, 147.8, 140.1, 139.4, 133.8, 124.8, 123.8, 119.6, 118.5, 102.5, 93.3, 90.3, 88.3, 55.0, 52.1;
HR ESI-MS (DCM/MeOH) m/z = 717.1426 [M+H]$^+$ (calc. for $C_{32}H_{29}N_8O_4IH^+$, 717.1435); m/z = 739.1246 [M+Na]$^+$ (calc. for $C_{32}H_{29}N_8O_4INa^+$, 739.1254);
IR v (cm^{-1}): 3136, 3058, 2956, 2936, 2836, 2214, 1584, 1461, 1365, 1236, 1124, 1074

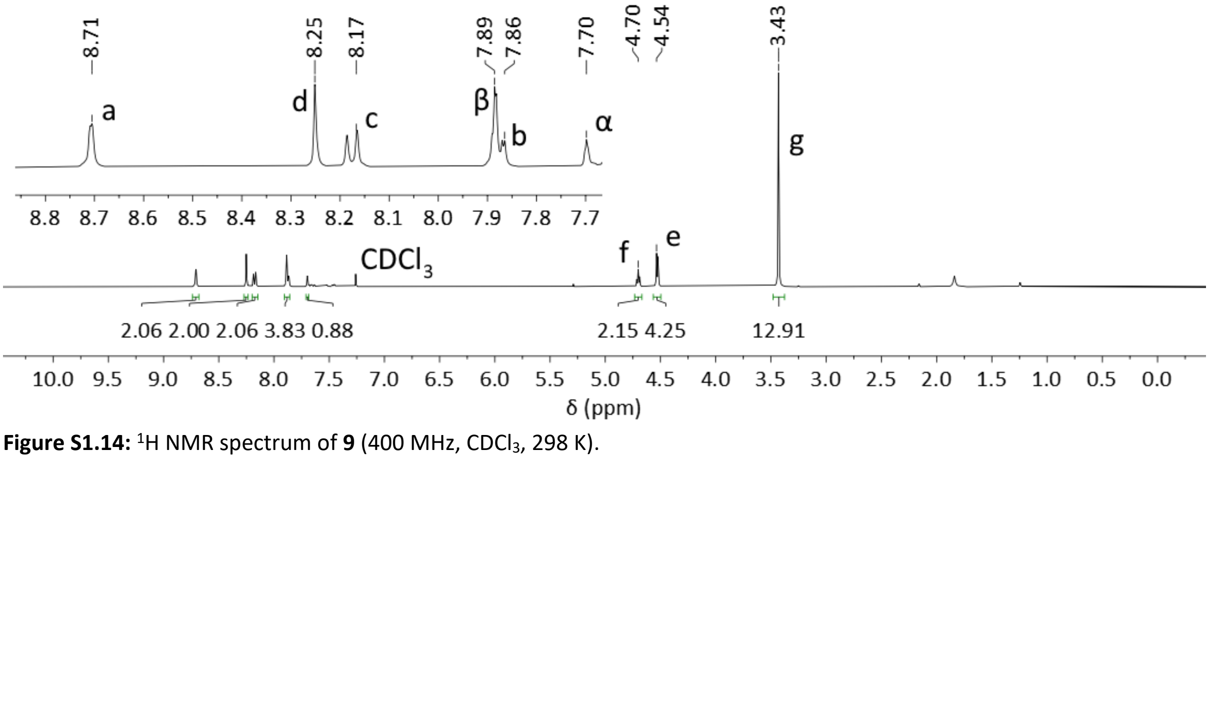

Figure S1.14: ^{1}H NMR spectrum of **9** (400 MHz, $CDCl_3$, 298 K).

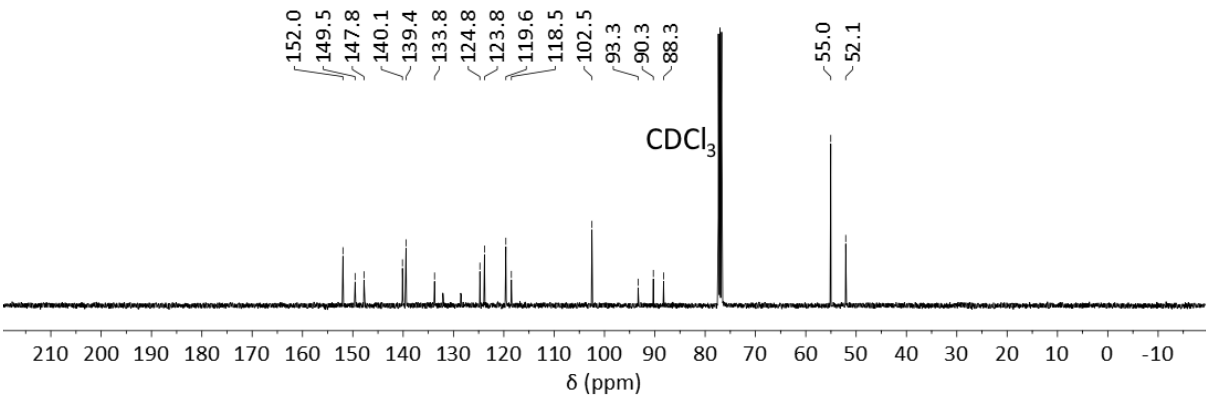

Figure S1.15: ^{13}C NMR spectrum of **9** (100 MHz, $CDCl_3$, 298 K).

1.2.9. 5-(3,5-Diiodophenyl)-2-(1-(2-methoxyethyl)-1H-1,2,3-triazol-4-yl)pyridine (10)

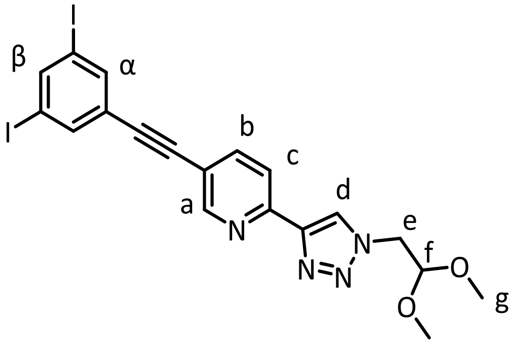

Compound **5** (180 mg, 0.545 mmol, 1.0 eq) and Na_2CO_3 (115 mg, 1.09 mmol, 2.0 eq) were stirred in MeOH (5 mL) in a flask open to the air for 2 hours. The solution was decanted and concentrated. The resulting solid was added to a solution of 1,3,5-triiodobenzene (1.24 g, 2.72 mmol, 5.0 eq) in THF (3 mL) and TEA (3 mL). The solution was degassed for 2 hr before copper (I) iodide (10.4 mg, 0.0.0545 mmol, 0.10 eq), [Pd_2(dibenzylideneacetone)$_3$] (12.5 mg, 0.0136 mmol, 0.025 eq) and triphenylphosphine (14.3 mg, 0.0545 mmol, 0.10 eq) were added. The resulting solution was stirred under a protective layer of N_2 overnight at 50 °C. 0.1M EDTA/$NH_4OH_{(aq)}$ (30 mL) was added and the organic layer was extracted in DCM (3 × 30 mL). The organic layers were combined and washed with brine (80 mL), and then concentrated to give a crude product. This was purified by flash column chromatography on silica eluting with a gradient of DCM to DCM/acetone 1:0.10 (v/v). Product was obtained as orange-red crystals (147 mg, 46%).

^{1}H NMR (400 MHz, $CDCl_3$, 298 K) δ: 8.69 (s, 1H, H_a), 8.26 (s, 1H, H_d), 8.16 (d, J = 8.2 Hz, 1H, H_c), 8.04 (t, J = 1.5 Hz, 1H, H_β), 7.87 (dd, J = 8.2, 2.0 Hz, 1H, H_b), 7.85 (d, J = 1.5 Hz, 2H, H_α). 4.71 (t, J = 5.2 Hz, 1H, H_f), 4.52 (d, J = 5.2 Hz, 2H, H_e), 3.43 (s, 6H, H_g);
^{13}C NMR (100 MHz, $CDCl_3$, 298 K) δ: 151.7, 149.4, 147.5, 145.3, 139.6, 139.4, 126.1, 124.0, 119.7, 118.4, 102.5, 94.2, 89.6, 88.6, 55.0, 52.1;
HR ESI-MS (DCM/MeOH) m/z = 586.9449 [M+H]$^+$ (calc. for $C_{19}H_{16}N_4O_2I_2H^+$, 586.9441); m/z = 608.9266 [M+Na]$^+$ (calc. for $C_{19}H_{16}N_4O_2I_2Na^+$, 608.9260);
IR ν (cm^{-1}): 3135, 3059, 2995, 2936, 2835, 2219, 1595, 1567, 1526, 1365, 1237, 1225, 1075, 853

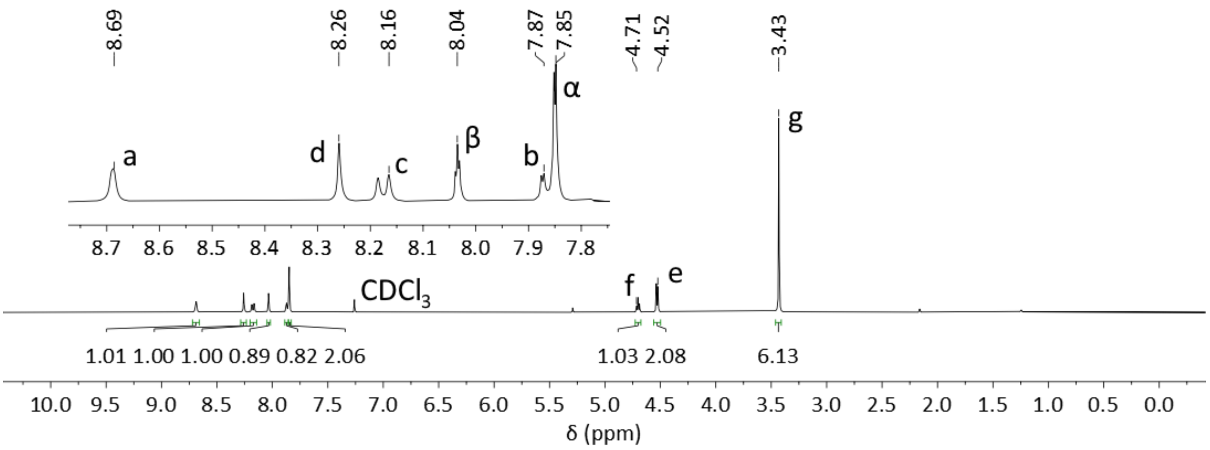

Figure S1.16: ^{1}H NMR spectrum of **10** (400 MHz, $CDCl_3$, 298 K).

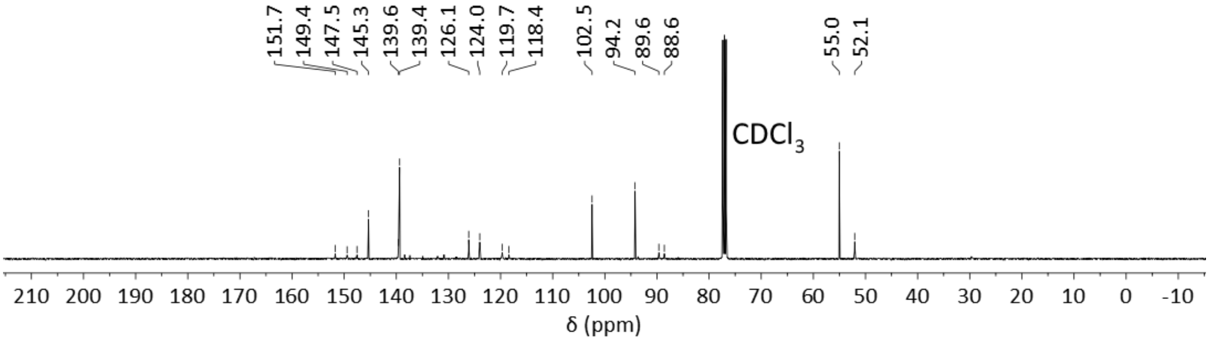

Figure S1.17: ^{13}C NMR spectrum of **10** (100 MHz, $CDCl_3$, 298 K).

1.2.10. 5-((3,5-diiodophenyl)buta-1,3-diyn-1-yl)-2-(1-(3,5-dimethoxybenzyl)-1H-1,2,3-triazol-4-yl)pyridine (11)

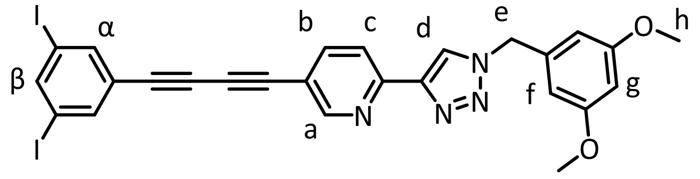

Compound **8** (329 mg, 0.790 mmol, 1.0 eq) was stirred in a solution of tetrabutylammonium fluoride (TBAF) (126 mg, 1.18 mmol, 1.5 eq) in THF (5 mL) in a vial open to air for 1 hr. Water (10 mL) and DCM (10 mL) were added, the organic fraction was collected and concentrated. This crude product was combined with 1,3,5-triiodobenzene (1.80 g, 3.95 mmol, 5.0 eq), THF (4 mL) and triethylamine (4 mL). The resulting solution was degassed for 1 hr before copper(I) iodide (15.0 mg, 0.0790 mmol, 0.10 eq), [Pd_2(dibenzlyideneacetone)$_3$] (18.1 mg, 0.0197 mmol, 0.0025 eq) and PPh_3 (20.8 mg, 0.0790 mmol, 0.010 eq) were added and the reaction mixture was stirred overnight at 50 °C. 0.1M EDTA/$NH_4OH_{(aq)}$ (25 mL) was added and the organic layer was extracted in DCM (3 × 25 mL). The organic layers were combined and washed with brine (75 mL), and then concentrated to give a crude product. This was purified by flash column chromatography on silica eluting with a gradient of DCM to DCM/acetone 1:0.05 (v/v). Product was obtained as a white powder (235 mg, 44%).

^{1}H NMR (400 MHz, $CDCl_3$, 298 K) *δ*: 8.12 (br s, 1H, H_a), 7.60 (d, *J* = 8.2 Hz, 1H, H_c), 7.53 (s, 1H, H_d), 7.51 (t, *J* = 1.4 Hz, 1H, $H_β$), 7.31 (dd, *J* = 8.3, 2.0 Hz, 1H, H_b), 7.26 (d, *J* = 1.4 Hz, 2H, $H_α$), 5.91 (d, *J* = 2.2 Hz, 2H, H_f), 5.89 (t, *J* = 2.2 Hz, 1H, H_g), 4.95 (s, 2H, H_e), 3.22 (s, 6H, H_h);
^{13}C NMR (100 MHz, $CDCl_3$, 298 K) *δ*: 161.4, 152.9, 149.9, 148.0, 146.1, 140.3, 140.1, 136.2, 125.1, 122.7, 119.5, 117.5, 106.4, 100.6, 94.1, 79.9, 79.2, 76.9, 75.9, 55.5, 54.6;
HR ESI-MS (DCM/MeOH) *m/z* = 672.9601 $[M+H]^+$ (calc. for $C_{26}H_{19}I_2N_4O_2$, 672.9592);
IR *ν* (cm^{-1}) 3307, 3138, 3058, 2954, 2837, 1648, 1595, 1569, 1522, 1476, 1462, 1429, 1211, 1164, 1071

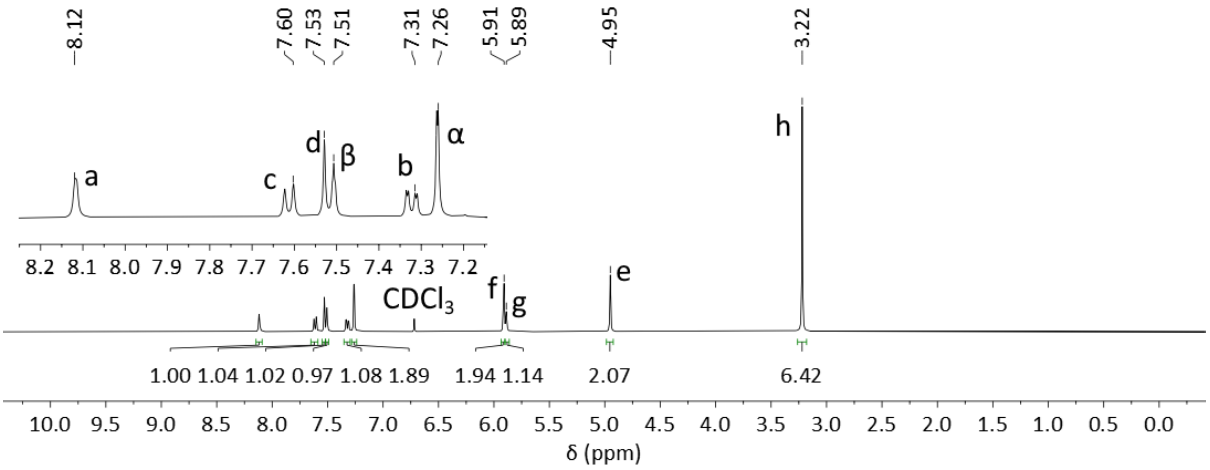

Figure S1.18: ^{1}H NMR spectrum of **11** (400 MHz, $CDCl_3$, 298 K).

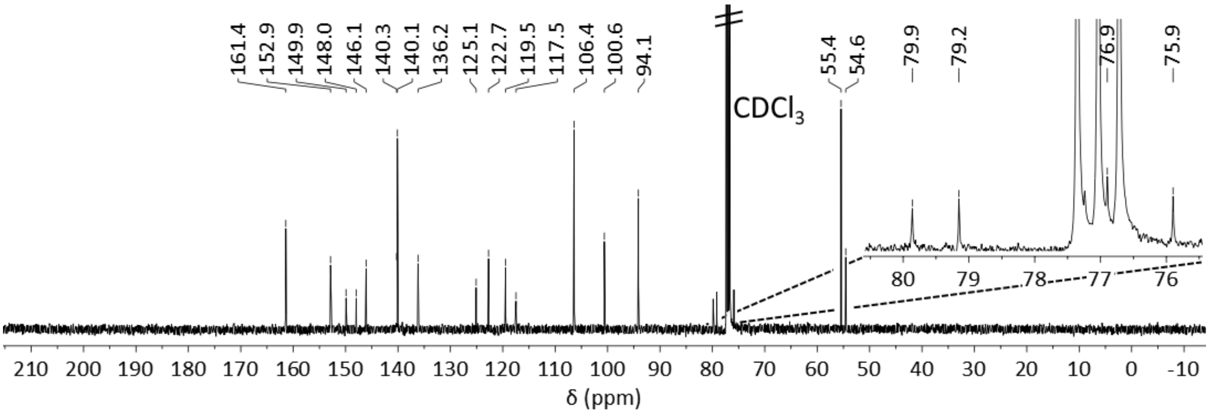

Figure S1.19: ^{13}C NMR spectrum of **11** (100 MHz, $CDCl_3$, 298 K).

1.2.11. 2-(1-(3,5-Dimethoxybenzyl)-1H-1,2,3-triazol-4-yl)-5-((3-((6-(1-(2,2-dimethoxyethyl)-1H-1,2,3-triazol-4-yl)pyridin-3-yl)ethynyl)-5-iodophenyl)buta-1,3-diyn-1-yl)pyridine (12)

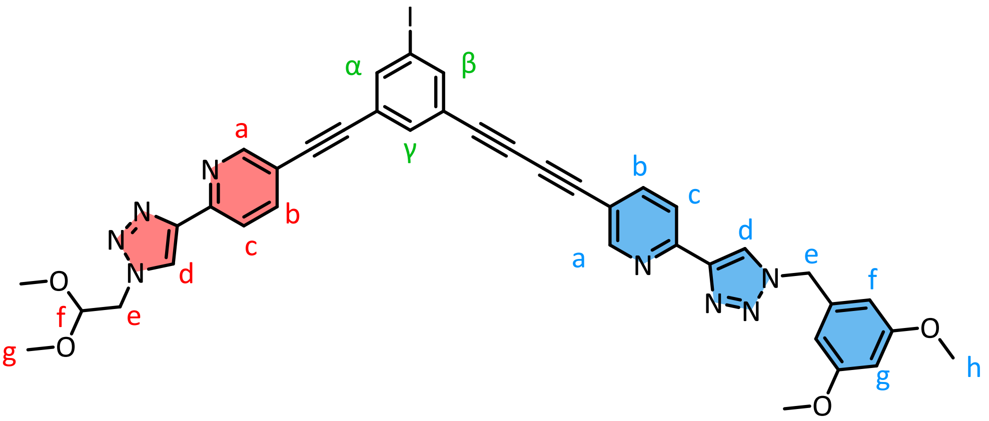

Compound **5** (98 mg, 0.30 mmol, 1.0 eq) and Na_2CO_3 (63 mg, 0.60 mmol, 2.0 eq) were stirred in MeOH (5 mL) in a flask open to the air for 2 hours. The solution was filtered to remove solid and concentrated. The crude product was added to a solution of compound **10** (1.00 g, 1.49 mmol, 5.0 eq) in THF (9 mL) and TEA (9 mL). The solution was degassed for 2 hr before copper (I) iodide (5.67 mg, 0.0298 mmol, 0.10 eq), [Pd_2(dibenzylideneacetone)$_3$] (6.81 mg, 7.44 µmol, 0.025 eq) and triphenylphosphine (7.82 mg, 0.0298 mmol, 0.10 eq) were added. The resulting solution was stirred under a protective layer of N_2 overnight at 50 °C. 0.1M EDTA/$NH_4OH_{(aq)}$ (30 mL) was added and the organic layer was extracted in DCM (3 × 30 mL). The organic layers were combined and washed with brine (80 mL), and then concentrated to give a crude product. This was purified by flash column chromatography on silica eluting with a gradient of DCM to DCM/acetone 4:1 (v/v). Product was obtained as off-white crystals (155 mg, 65%).

^1H NMR (400 MHz, $CDCl_3$, 298 K) δ: 8.71 (d, *J* = 1.5 Hz, 1H, H_a), 8.68 (d, *J* = 1.5 Hz, 1H, H_a), 8.24 (s, 1H, H_d), 8.18 (d, *J* = 8.0 Hz, 1H, H_c), 8.16 (d, *J* = 8.0 Hz, 1H, H_c), 8.08 (s, 1H, H_d), 7.91 (t, *J* = 1.4 Hz, 1H, H_α), 7.89 (dd, *J* = 8.3, 2.0 Hz, 1H, H_b), 7.87 (dd, *J* = 8.2, 2.0 Hz, 1H, H_b), 7.85 (t, *J* = 1.5 Hz, 1H, H_β), 7.66 (t, *J* = 1.4 Hz, 1H, H_γ), 6.45 (d, *J* = 2.1 Hz, 2H, H_f), 6.44 (t, *J* = 2.1 Hz, 1H, H_g), 5.50 (s, 2H, H_e), 4.71 (t, *J* = 5.2 Hz, 1H, H_f), 4.54 (d, *J* = 5.2 Hz, 2H, H_e), 3.77 (s, 6H, H_h), 3.44 (s, 6H, H_g);
^{13}C NMR (200 MHz, $CDCl_3$, 298 K) δ: 161.5, 153.0, 152.1, 150.0, 149.8, 148.1, 147.9, 141.0, 141.0, 140.4, 139.6, 136.3, 134.6, 125.0, 124.0, 123.8, 122.8, 119.7, 119.6, 118.5, 117.7, 106.5, 102.6, 100.7, 93.4, 90.1, 88.6, 80.1, 79.7, 77.1, another peak lies here but is obscured by solvent peak, 75.6, 55.6, 55.2, 54.7, 52.2;
HR ESI-MS ($CDCl_3$/MeOH) *m/z* = 803.1576 $[MH]^+$ (calc. for $[C_{39}H_{32}IN_8O_4]^+$, 803.1586); *m/z* = 825.1399 $[MNa]^+$ (calc. for $[C_{39}H_{31}IN_8O_4Na]^+$, 825.1405);
IR *v* (cm^{-1}): 3146, 3064, 3006, 2954, 2936, 2838, 2221, 1594, 1453, 1461, 1430, 1365, 1235, 1206, 1158, 1068, 1043, 981

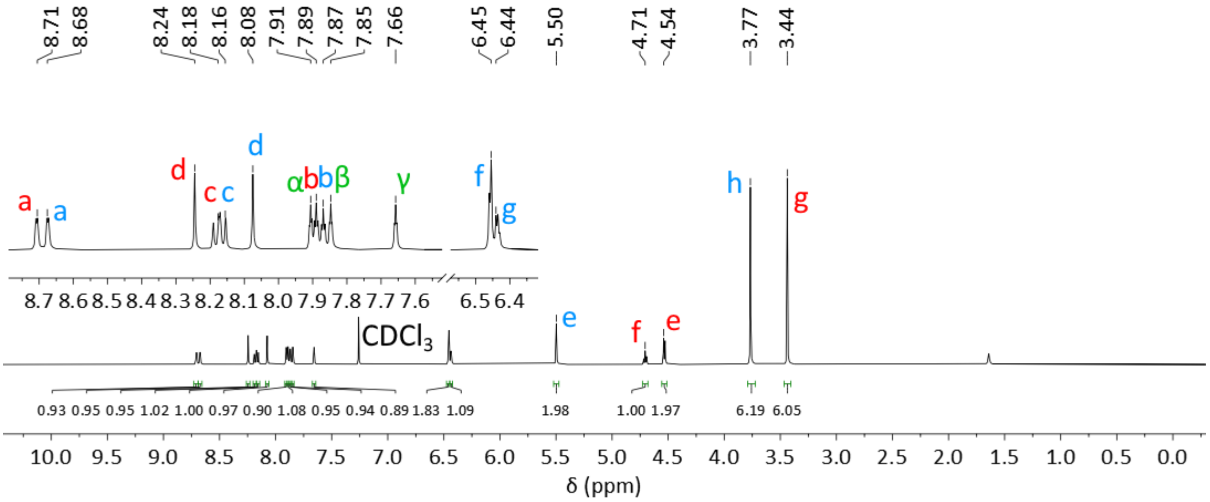

Figure S1.20: ^1H NMR spectrum of **12** (400 MHz, $CDCl_3$, 298 K).

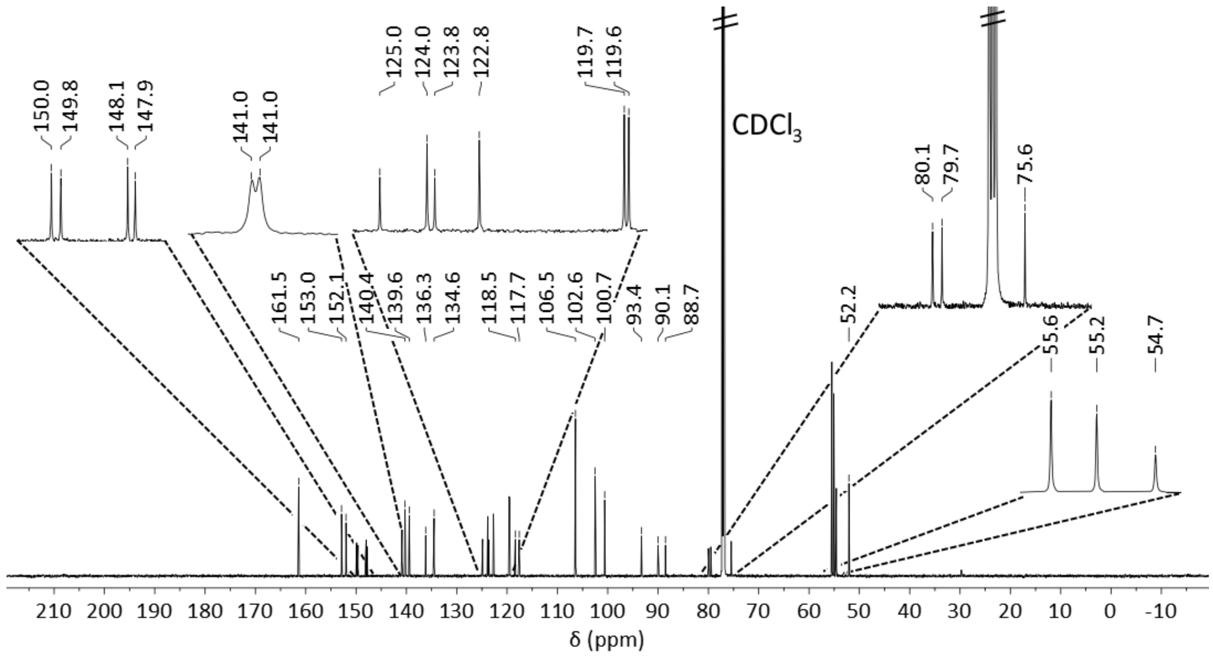

Figure S1.21: ^{13}C NMR spectrum of **12** (200 MHz, $CDCl_3$, 298 K).

1.3. Ligands

1.3.1. 1,3,5-tris(6-(1-(2-methoxyethyl)-1H-1,2,3-triazol-4-yl)pyridin-3-yl)benzene (L_{111})

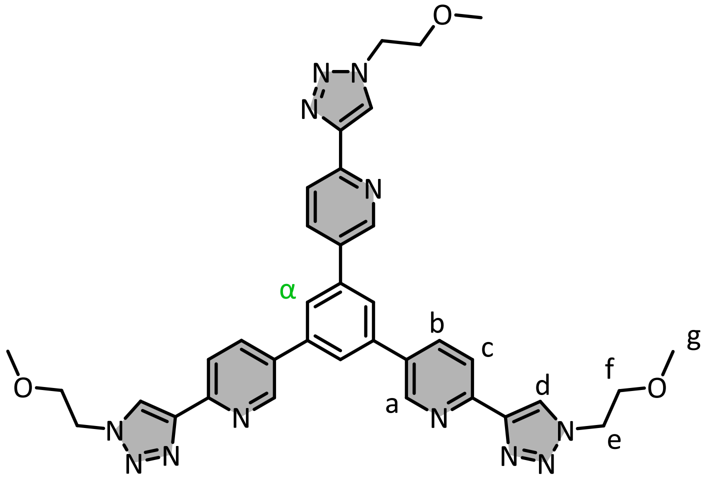

1,3,5-triiodobenzene (35 mg, 0.0757 mmol, 1.0 eq), crude **3** (100 mg, ~4 eq) and K_2CO_3 (52 mg, 0.379 mmol, 5.0 eq) were stirred in dioxane (3 mL) in a sealed tube. The resulting solution was degassed for 150 minutes under an N_2 atmosphere. [Pd_2(dibenzlyideneacetone)$_3$] (1.7 mg, 1.9 µmol, 0.0025 eq) and PPh_3 (2.0 mg, 7.6 µmol, 0.010 eq) were added and the reaction mixture was stirred overnight at 150 °C. DCM and water (both 20 mL) were added. The organic layer was extracted and then washed once with water (20 mL). This was purified by flash column chromatography on silica eluting with a gradient of DCM/Acetone 3:1 (v/v) to DCM/Acetone 1:9 (v/v). Product was obtained as a white powder (14 mg, 27%).

^{1}H NMR (400 MHz, $CDCl_3$, 298 K) δ: 8.95 (br s, 1H, H_a), 8.36 (s, 1H, H_d), 8.32 (d, J = 8.3 Hz, 1H, H_c), 8.13 (dd, J = 8.3, 2.1 Hz, 1H, H_b), 7.87 (s, 1H, H_α), 4.63 (d, J = 5.0 Hz, 2H, H_e), 3.83 (d, J = 5.0 Hz, H_f), 3.39 (s, 3H, H_g);

^{13}C NMR: A ^{13}C NMR spectrum was unable to be obtained due to solubility issues;

HR ESI-MS ($CDCl_3$/MeOH) m/z = 685.3118 $[MH]^+$ (calc. for $C_{36}H_{37}N_{12}O_3^+$, 685.3112); m/z = 707.2922 $[MNa]^+$ (calc. for $C_{36}H_{36}N_{12}O_3Na^+$, 707.2931);

IR ν (cm^{-1}) 2924, 2854, 1736, 1601, 1541, 1463, 1439, 1352, 1229, 1116, 1044, 844, 814

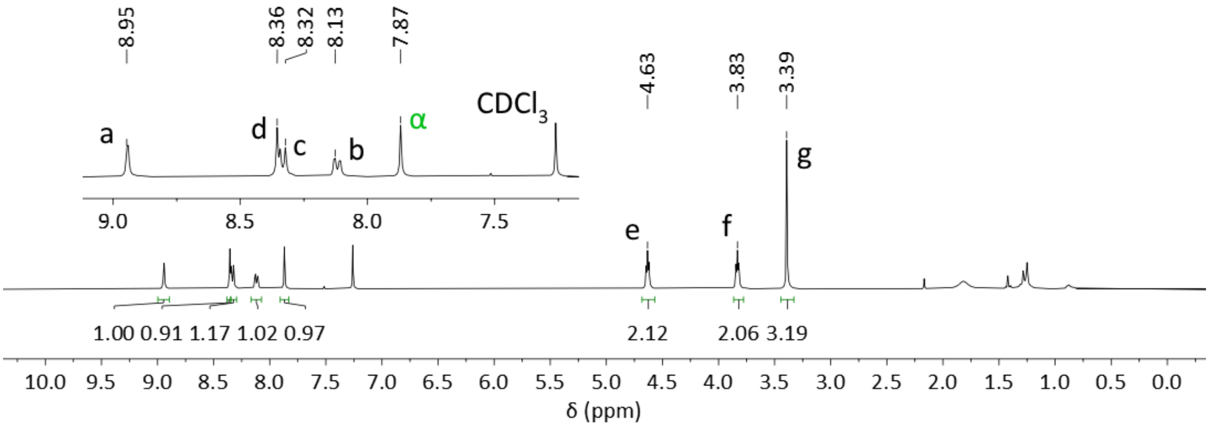

Figure S1.22: ^{1}H NMR spectrum of $\mathbf{L_{111}}$. (400 MHz, $CDCl_3$, 298 K).

1.3.2. 5,5'-(5-((6-(1-(2,2-dimethoxyethyl)-1H-1,2,3-triazol-4-yl)pyridin-3-yl)ethynyl)-1,3-phenylene)bis(2-(1-(2-methoxyethyl)-1H-1,2,3-triazol-4-yl)pyridine) (L_{112})

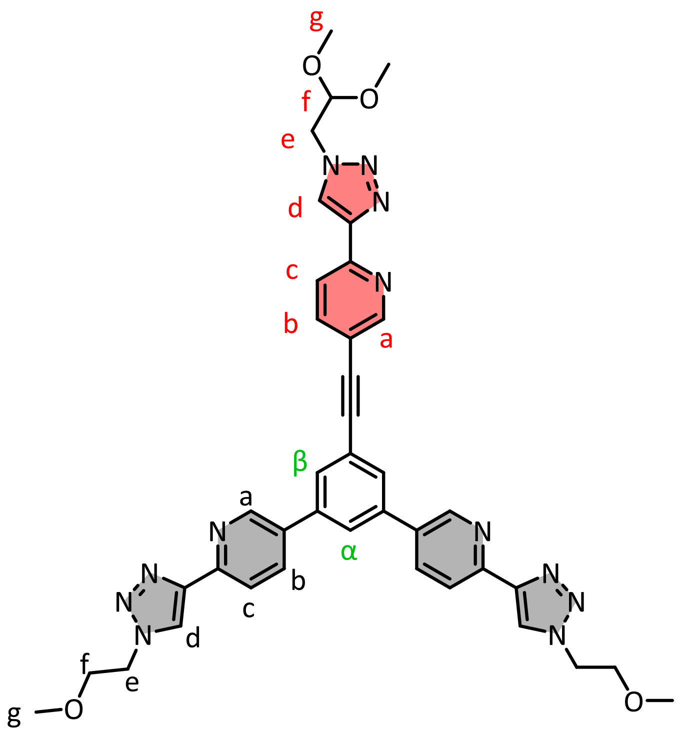

Compound **10** (120 mg, 0.205 mmol, 1.0 eq), crude **3** (203 mg, ~3 eq) and K_2CO_3 (141 mg, 1.02 mmol, 5.0 eq) were stirred in dioxane (10 mL). The resulting solution was degassed for 2 hrs under an N_2 atmosphere. $[Pd_2(dibenzlyideneacetone)_3]$ (4.7 mg, 5.1 µmol, 0.0025 eq) and PPh_3 (5.4 mg, 0.021 mmol, 0.010 eq) were added and the reaction mixture was stirred overnight at 100 °C. DCM and

water (both 30 mL) were added. The organic layer was extracted and then washed once with water (30 mL). This was purified by flash column chromatography on silica eluting with a gradient of DCM/Acetone to DCM/Acetone 1:4 (v/v). Product was obtained as a white powder (42 mg, 28%).

^{1}H NMR (400 MHz, $CDCl_3$, 298 K) δ: 8.93 (d, J = 1.9 Hz, 2H, H_a), 8.82 (d, J = 1.5 Hz, 1H, H_a), 8.42 (br s, 2H, H_d), 8.35 (d, J = 8.2 Hz, 2H, H_c), 8.30 (s, 1H, H_d), 8.23 (d, J = 8.2 Hz, 1H, H_c), 8.08 (d, J = 8.1 Hz, 2H, H_b), 7.98 (dd, J = 8.2, 2.0 Hz, 1H, H_b), 7.88 (d, J = 1.5 Hz, 2H, H_β), 7.85 (t, J = 1.6 Hz, 1H, H_α), 4.72 (t, J = 5.3 Hz, 1H, H_f), 4.63 (t, J = 5.0 Hz, 4H, H_e), 4.54 (d, J = 5.3 Hz, 2H, H_e), 3.84 (t, J = 5.0 Hz, 4H, H_f), 3.47 (s, 6H, H_g), 3.42 (s, 6H, H_g);
^{13}C NMR (200 MHz, $CDCl_3$, 298 K) δ: 152.2, 150.1, 149.6, 148.0 (3 peaks), 139.6, 139.2, 135.6, 134.5, 129.8, 126.1, 124.7, 123.9, 120.5, 119.8, 119.0, 102.7, 92.2, 87.7, 70.8, 59.2, 55.2, 52.2, 50.7;
HR ESI-MS ($CDCl_3$/MeOH) m/z = 739.3214 $[MH]^+$ (calc. for $[C_{39}H_{38}N_{12}O_4H]^+$, 739.3217); m/z = 761.3033 $[MNa]^+$ (calc. for $[C_{39}H_{38}N_{12}O_4Na]^+$, 761.3033);
IR ν (cm^{-1}) 3137, 2933, 2835, 2244, 1598, 1484, 1461, 1365, 1234, 1121, 1074, 1044, 8352

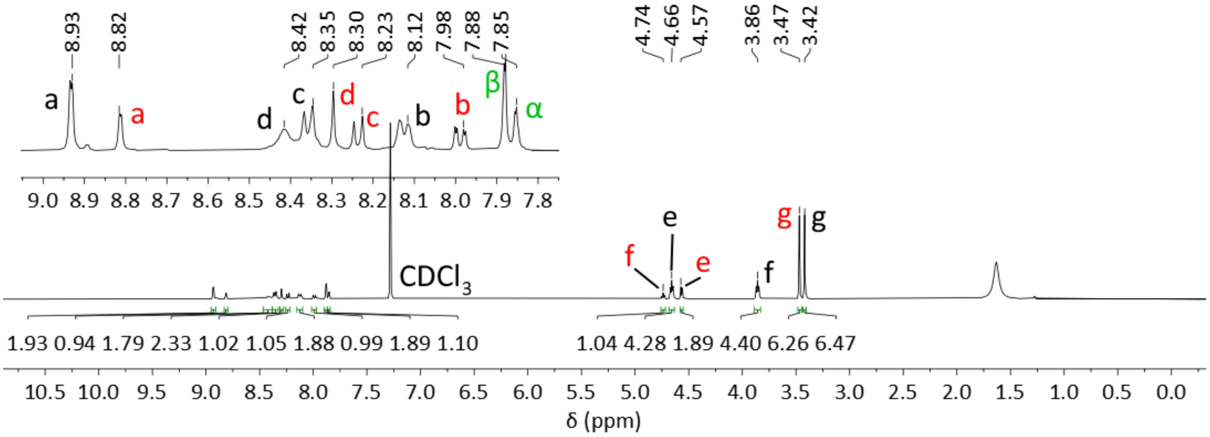

Figure S1.23: ^{1}H NMR spectrum of $\mathbf{L_{112}}$. (400 MHz, $CDCl_3$, 298 K).

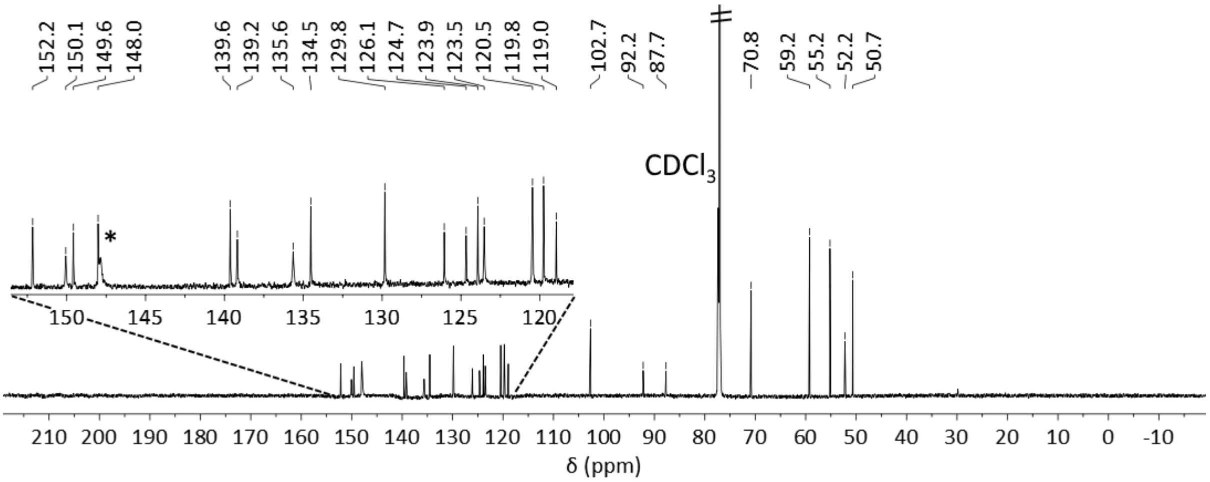

Figure S1.24: ^{13}C NMR spectrum of $\mathbf{L_{112}}$. (200 MHz, $CDCl_3$, 298 K).

1.3.3. 5,5'-((5-(6-(1-(2-methoxyethyl)-1H-1,2,3-triazol-4-yl)pyridin-3-yl)-1,3-phenylene)bis(ethyne-2,1-diyl))bis(2-(1-(2,2-dimethoxyethyl)-1H-1,2,3-triazol-4-yl)pyridine) (L_{122})

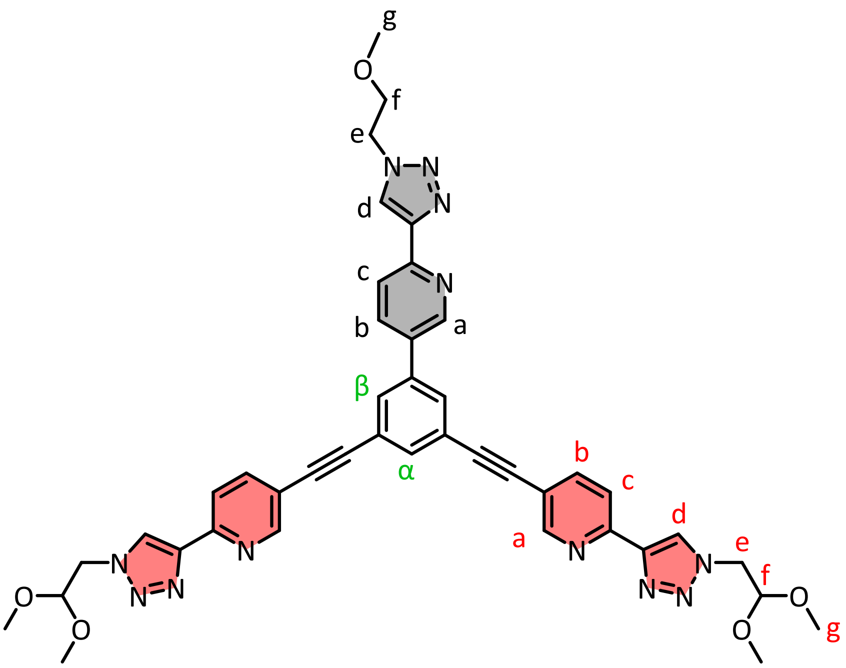

Compound **9** (75 mg, 0.11 mmol, 1.0 eq), crude **3** (70 mg, ~2 eq) and K_2CO_3 (72 mg, 0.52 mmol, 5.0 eq) were stirred in dioxane (8 mL). The resulting solution was degassed for 2 hrs under an N_2 atmosphere. [Pd_2(dibenzlyideneacetone)$_3$] (2.4 mg, 2.6 µmol, 0.0025 eq) and PPh_3 (2.8 mg, 0.011 mmol, 0.010 eq) were added and the reaction mixture was stirred overnight at 100 °C. DCM and water (both 30 mL) were added. The organic layer was extracted and then washed once with water (30 mL). This was purified by flash column chromatography on silica eluting with a gradient of DCM (v/v) to DCM/Acetone 1:1 (v/v). Product was obtained as a white powder (51 mg, 61%).

^{1}H NMR (400 MHz, $CDCl_3$, 298 K) δ: 8.87 (br s, 1H, H_a), 8.76 (br s, 2H, H_a), 8.42 (br s, 1H, H_d), 8.33 (d, J = 8.4 Hz, 1H, H_c), 8.27 (s, 2H, H_d), 8.20 (d, J = 8.4 Hz, 2H, H_c), 8.08 (dd, J = 8.4, 2.1 Hz, 1H, H_b), 7.93 (dd, J = 8.4, 2.1 Hz, 2H, H_b), 7.80 (m, 3H, $H_{\alpha,\ \beta}$), 4.72 (t, J = 4.9 Hz, 2H, H_f), 4.63 (t, J = 4.9 Hz, 2H, H_e), 4.54 (d, J = 5.6 Hz, 4H, H_e), 3.84 (t, J = 5.1 Hz, 2H H_f), 3.45 (s, 12H, H_g), 3.40 (s, 3H, H_g);
^{13}C NMR (200 MHz, $CDCl_3$, 298 K) δ: 152.2, 150.2, 149.6, 148.0 (2 peaks), 147.8, 139.6, 138.7, 135.5, 134.2, 134.0, 130.3, 124.2, 123.9, 123.5, 120.4, 119.8, 118.9, 102.7, 91.7, 87.8, 70.8, 59.2, 55.2, 52.2, 50.7;
HR ESI-MS ($CDCl_3$/MeOH) m/z = 815.3131 $[MNa]^+$ (calc. for $[C_{42}H_{40}N_{12}O_5Na]$, 815.3142);
IR v (cm^{-1}) 2957, 2925, 2857, 1589, 1462, 1488, 1239, 1124, 1077

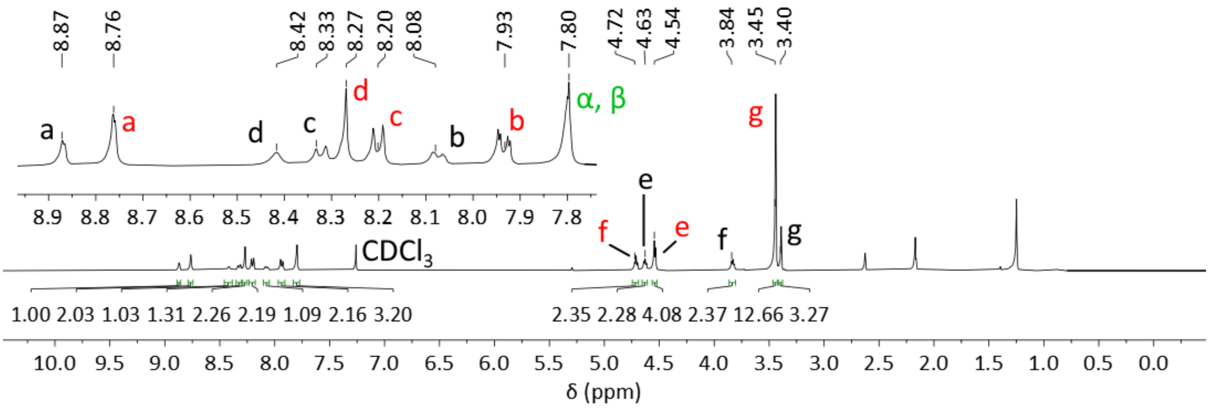

Figure S1.25: ^{1}H NMR spectrum of $\mathbf{L_{122}}$. (400 MHz, $CDCl_3$, 298 K).

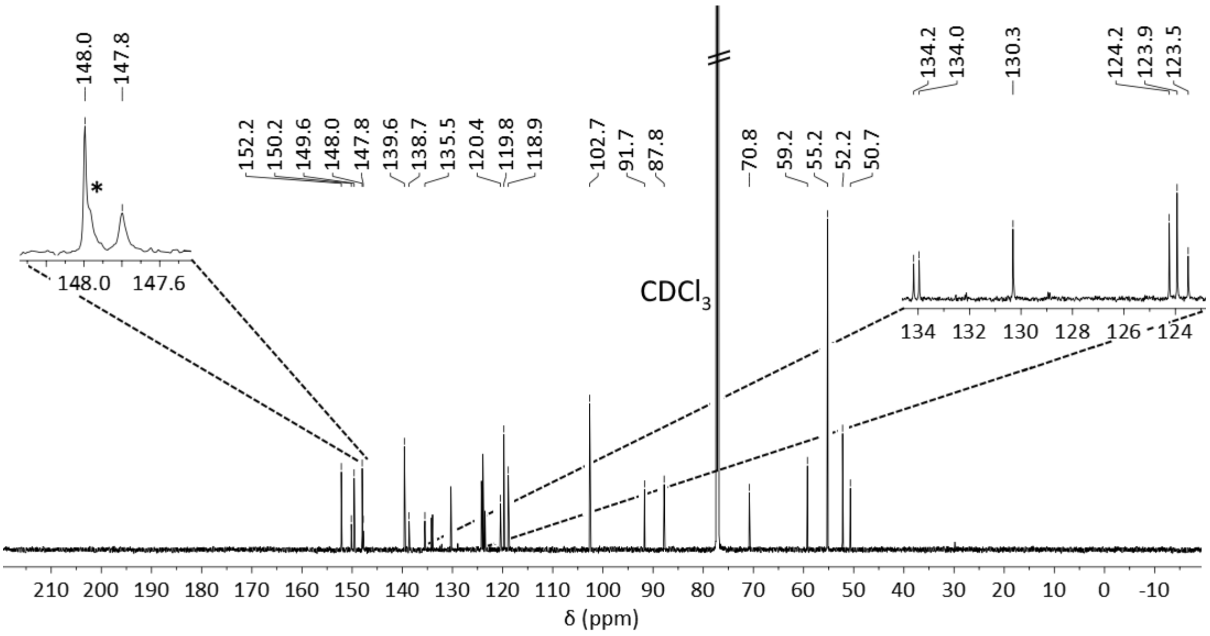

Figure S1.26: ^{13}C NMR spectrum of $\mathbf{L_{122}}$. (200 MHz, $CDCl_3$, 298 K).

1.3.4. 5,5'-(5-((6-(1-(3,5-dimethoxybenzyl)-1H-1,2,3-triazol-4-yl)pyridin-3-yl)buta-1,3-diyn-1-yl)-1,3-phenylene)bis(2-(1-(2-methoxyethyl)-1H-1,2,3-triazol-4-yl)pyridine) (L_{113})

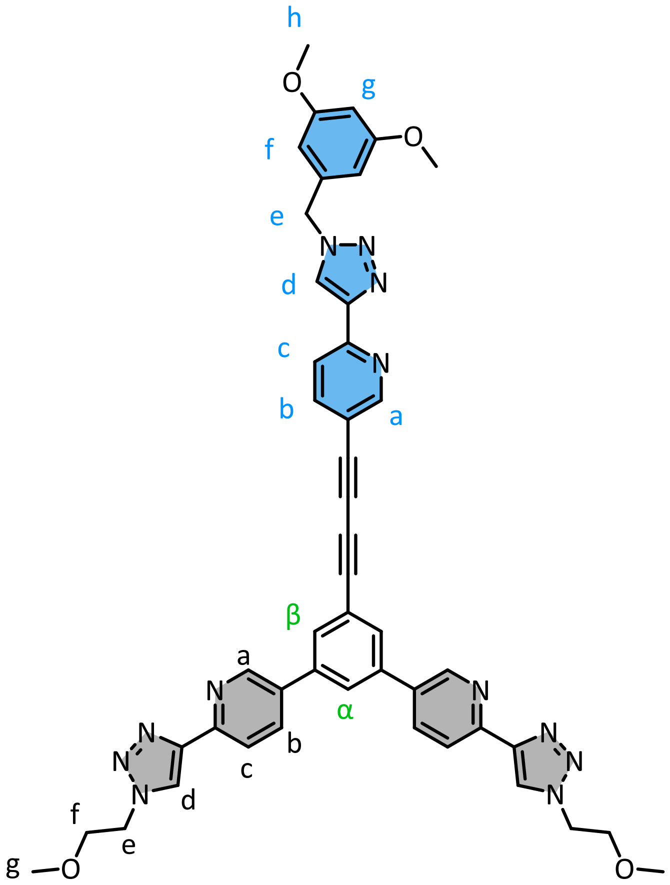

Compound **10** (130 mg, 0.193 mmol, 1.0 eq), crude **3** (192 mg, ~3 eq) and K_2CO_3 (134 mg, 0.967 mmol, 5.0 eq) were stirred in dioxane (5 mL). The resulting solution was degassed for 2 hrs under an N_2 atmosphere. [Pd_2(dibenzlyideneacetone)$_3$] (4.4 mg, 4.8 µmol, 0.0025 eq) and PPh_3 (5.1 mg, 0.019 mmol, 0.010 eq) were added and the reaction mixture was stirred overnight at 100 °C. DCM and water (both 40 mL) were added. The organic layer was extracted and then washed once with water (40 mL). This was purified by flash column chromatography on silica eluting with a gradient of DCM to DCM/Acetone 1:3 (v/v). Product was obtained as an orange powder (23 mg, 14%).

^{1}H NMR (400 MHz, $CDCl_3$, 298 K) δ: 8.86 (d, J = 1.6 Hz, 2H, H_a), 8.69 (d, J = 1.5 Hz, 1H, H_a), 8.32 (s, 2H, H_d), 8.28 (d, J = 8.2 Hz, 2H, H_c), 8.15 (d, J = 8.2 Hz, 1H, H_c), 8.08 (s, 1H, H_d), 8.02 (dd, J = 8.2, 2.2 Hz, 2H, H_b), 7.89 (dd, J = 8.2, 2.0 Hz, 1H, H_b), 7.83 (br s, 1H, H_α), 7.81 (s, 2H, H_β), 6.45 (d, J = 2.1 Hz, 2H, H_f), 6.43 (t, J = 2.1 Hz, 1H, H_g), 5.50 (s, 2H, H_e), 4.63 (t, J = 5.0 Hz, 4H, H_e), 3.82 (t, J = 5.0 Hz, 4H, H_f), 3.76 (s, 6H, H_h), 3.38 (s, 6H, H_g);
^{13}C NMR (200 MHz, $CDCl_3$, 298 K) δ: 161.5, 153.0, 150.2, 149.1, 148.1, 148.0, 147.9, 140.4, 139.3, 136.3, 135.4, 134.2, 130.5, 126.8, 123.4 (2 peaks), 122.8, 120.4, 119.6, 117.8, 106.5, 100.7, 82.1, 79.2, another peak lies here but is obscured by solvent peak, 74.8, 70.8, 59.2, 55.6, 54.7, 50.6;
HR ESI-MS (DCM/MeOH) m/z = 825.3351 $[MH]^+$ (calc. for $[C_{46}H_{41}N_{12}O_4]^+$, 825.3368);
IR ν (cm^{-1}): 3140 3064, 2927, 2995, 2927, 2908, 2840, 2246, 1711, 1594, 1461, 1430, 1359, 1234, 1205, 1158, 1120, 1067, 1043, 836

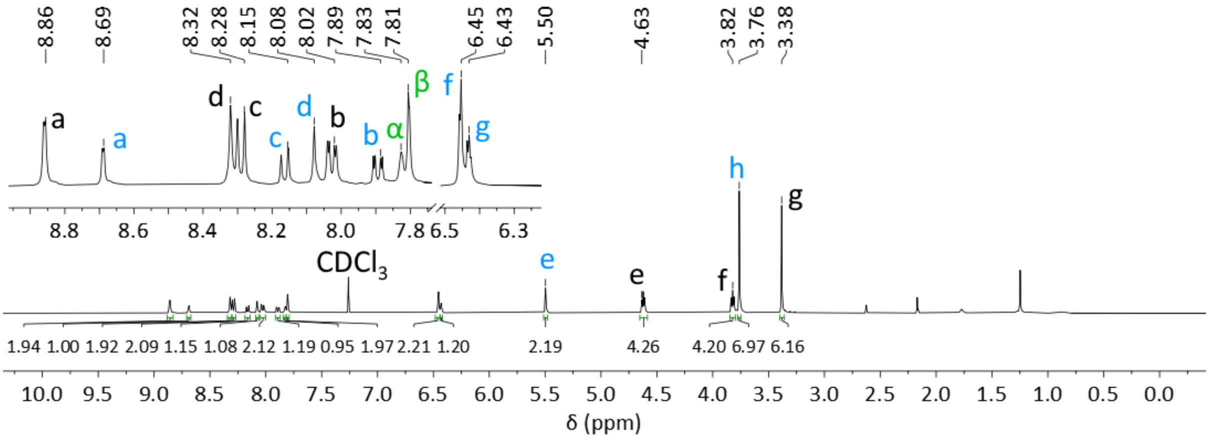

Figure S1.27: ^{1}H NMR spectrum of $\mathbf{L_{113}}$. (400 MHz, $CDCl_3$, 298 K).

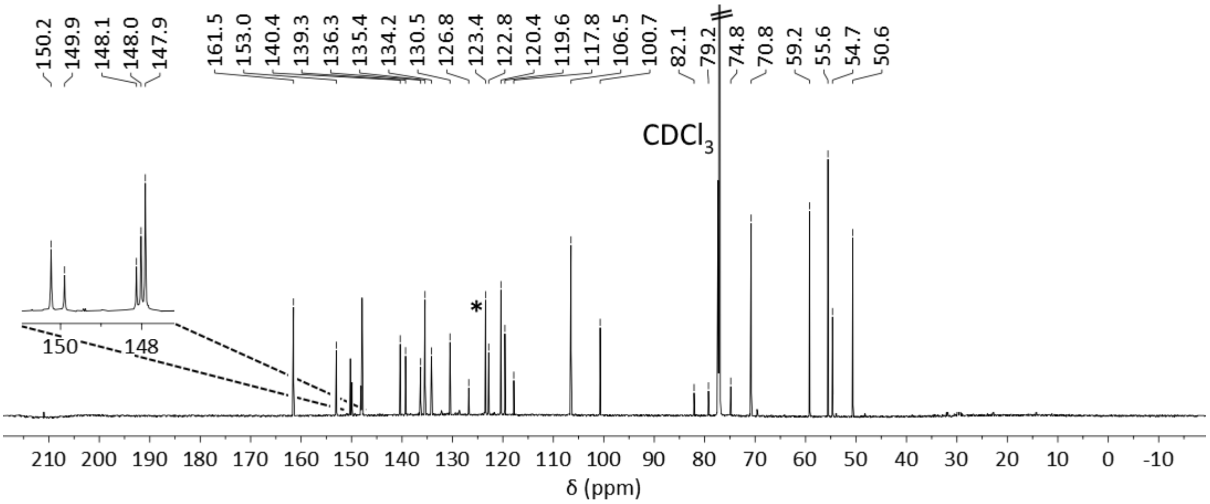

Figure S1.28: ^{13}C NMR spectrum of $\mathbf{L_{113}}$. (200 MHz, $CDCl_3$, 298 K).

1.3.5. 2-(1-(3,5-dimethoxybenzyl)-1H-1,2,3-triazol-4-yl)-5-((3-((6-(1-(2,2-dimethoxyethyl)-1H-1,2,3-triazol-4-yl)pyridin-3-yl)ethynyl)-5-(6-(1-(2-methoxyethyl)-1H-1,2,3-triazol-4-yl)pyridin-3-yl)phenyl)buta-1,3-diyn-1-yl)pyridine (L_{123})

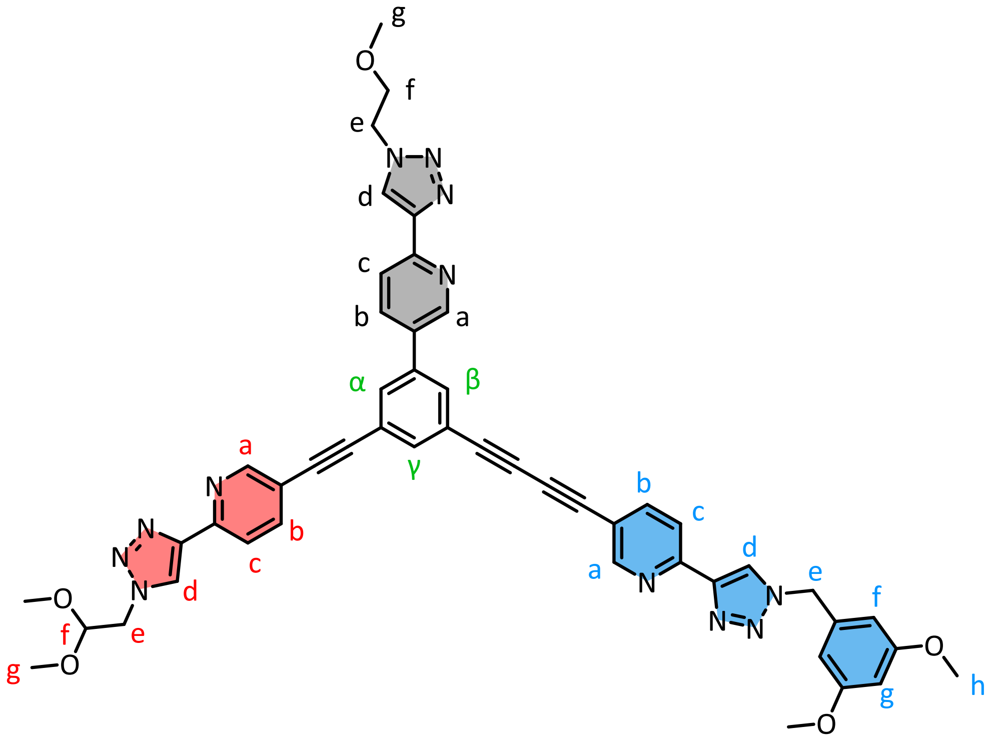

Compound **12** (160 mg, 0.199 mmol, 1.0 eq), crude **3** (132 mg, ~2 eq) and K_2CO_3 (138 mg, 0.997 mmol, 5.0 eq) were stirred in dioxane (10 mL). The resulting solution was degassed for 2 hrs under an N_2 atmosphere. [Pd_2(dibenzlyideneacetone)$_3$] (4.6 mg, 5.0 µmol, 0.0025 eq) and PPh_3 (5.2 mg, 0.020 mmol, 0.010 eq) were added and the reaction mixture was stirred overnight at 100 °C. DCM and water (both 30 mL) were added. The organic layer was extracted and then washed once with water (30 mL). This was purified by flash column chromatography on silica eluting with a gradient of DCM to DCM/Acetone 3:2 (v/v). Product was obtained as a yellow powder (81 mg, 46%).

^{1}H NMR (400 MHz, $CDCl_3$, 298 K) δ: 8.86 (d, J = 1.9 Hz, 1H, H_a), 8.78 (d, J = 1.9 Hz, 1H, H_a), 8.72 (d, J = 1.9 Hz, 1H, H_a), 8.34 (s, 1H, H_d), 8.32 (d, J = 8.2 Hz, 1H, H_c), 8.28 (s, 1H, H_d), 8.21 (m, 2H, $H_{c,c}$), 8.10 (s, 1H, H_d), 8.03 (dd, J = 8.2, 2.4 Hz, 1H, H_b), 7.92 (dd, J = 8.1, 2.2 Hz, 1H, H_b), 7.83 (dd, J = 8.1, 2.1, 1H, H_b), 7.83 (m, 1H, H_β), 7.79-7.77(m, 2H, $H_{\alpha,\gamma}$), 6.48 (d, J = 2.1 Hz, 2H, H_f), 6.46 (t, J = 2.2 Hz, 1H, H_g), 5.53 (s, 2H, H_e), 4.74 (t, J = 5.2 Hz, 1H, H_f), 4.65 (t, J = 5.0 Hz, 2H, H_e), 4.57 (d, J = 5.3 Hz, 2H, H_e), 3.85 (t, J = 4.9 Hz, 2H, H_f), 3.79 (s, 6H, H_h), 3.47 (s, 6H, H_g), 3.41 (s, 3H, H_g);
^{13}C NMR (200 MHz, $CDCl_3$, 298 K) δ: 161.5, 153.0, 152.2, 150.3, 150.0, 149.7, 148.2, 148.0, 148.0, 147.8, 140.4, 139.6, 138.8, 136.3, 135.4, 134.8, 133.7, 131.1, 131.0, 124.4, 123.9, 123.5, 123.1, 122.8, 120.4, 119.7, 119.6, 118.7, 117.8, 106.5, 102.6, 100.8, 91.4, 88.0, 81.5, 79.3, another peak lies here but is obscured by solvent peak, 75.0, 70.8, 59.2, 55.6, 55.2, 54.7, 52.2, 50.7;
HR ESI-MS ($CDCl_3$/MeOH) m/z = 879.3466 [MH]$^+$ (calc. for [$C_{49}H_{43}N_{12}O_5$]$^+$, 879.3474); m/z = 901.3285 [MNa]$^+$ (calc. for [$C_{49}H_{42}N_{12}O_5Na$]$^+$, 901.3293);
IR ν (cm^{-1}): 3138, 3067, 3006, 2934, 2837, 2230, 1595, 1461, 1360, 1235, 1206, 1158, 1123, 1068, 1043, 981, 848

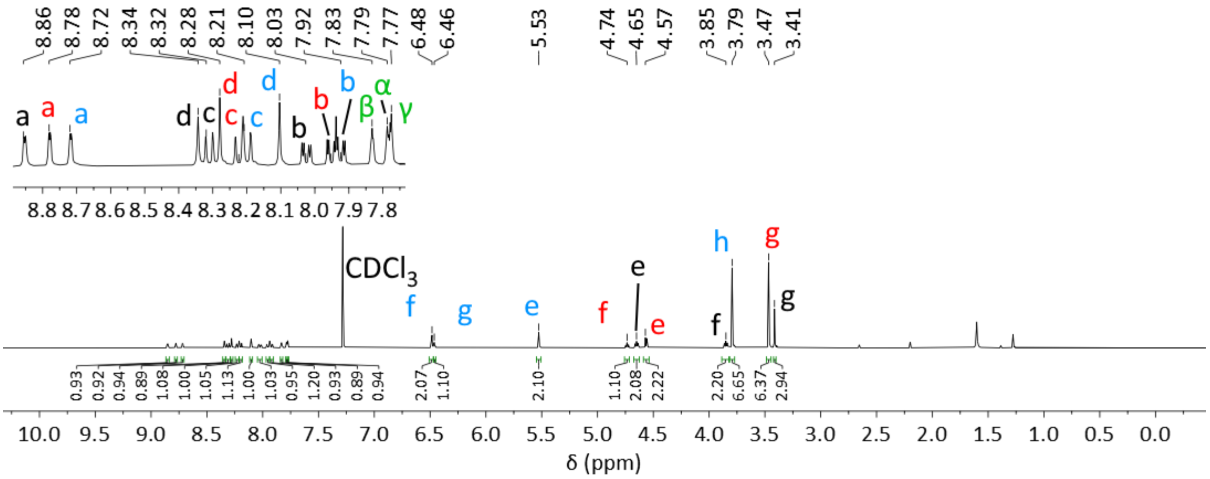

Figure S1.29: ^{1}H NMR spectrum of $\mathbf{L_{113}}$. (400 MHz, $CDCl_3$, 298 K).

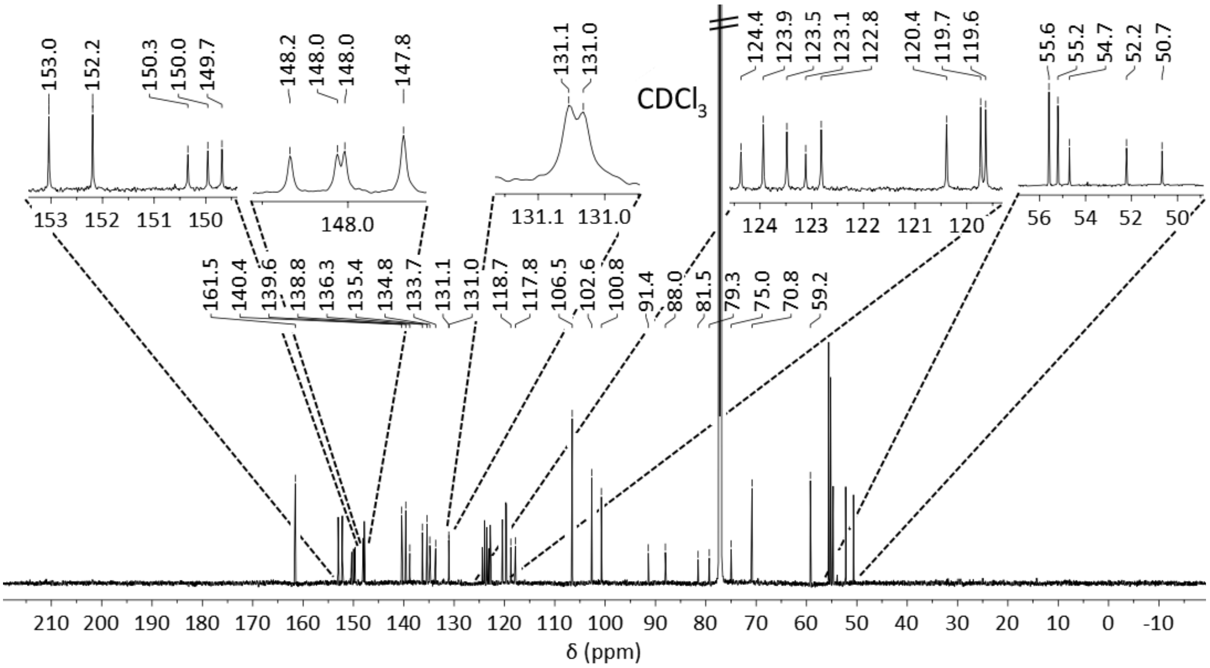

Figure S1.30: ^{13}C NMR spectrum of $\mathbf{L_{123}}$. (200 MHz, $CDCl_3$, 298 K).

1.3.6. 1,3,5-tris(6-(1-(3,5-dimethoxybenzyl)-1H-1,2,3-triazol-4-yl)pyridin-3-yl)benzene (L_{111}‘)

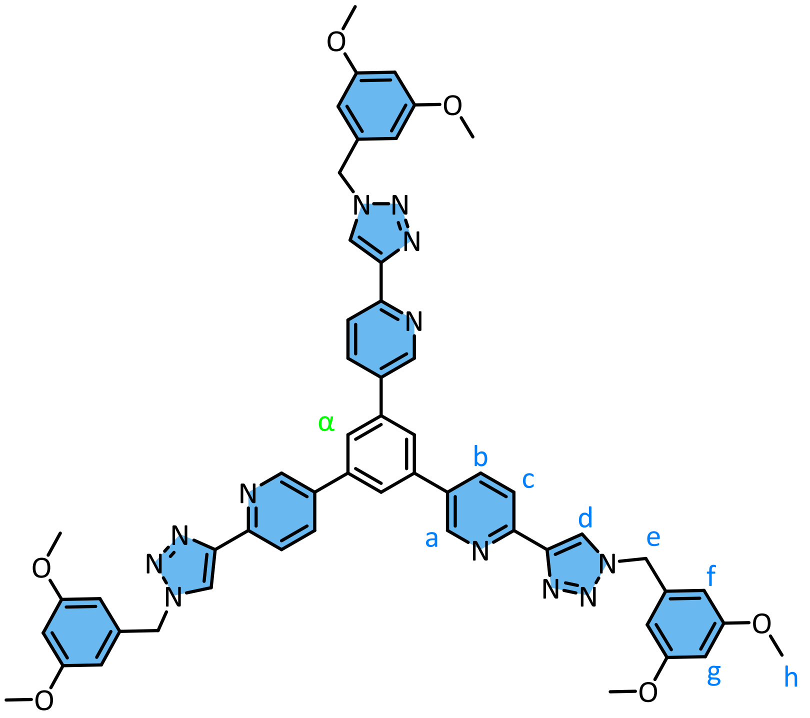

Bis(pinacolato)diboron (208 mg, 0.821 mmol, 6 eq), **6** (280 mg, 7.46 mmol, 5.5 eq) and KOAc (220 mg, 2.24 mmol, 16 eq) were stirred in DMF (10 mL). The resulting mixture was degassed with N_2 for 1 hr. 1,1'-Bis(diphenylphosphino)ferrocene (18 mg, 0.037 mmol, 0.3 eq) and [Pd($CH_3CN)_2Cl_2$] (9 mg, 0.04 mmol, 0.3 eq) were added under an N_2 atmosphere and the resulting solution was stirred at 85 °C for 48 hrs. The solvent was removed under vacuum, before adding DCM (50 mL) and water (50 mL) were added. The organic layer was washed with water (50 mL) and the solvent removed under vacuum. The residue was combined with 1,3,5-triiodobenzene (62 mg, 0.14 mmol, 1.0 eq), and K_2CO_3 (91 mg, 0.66 mmol, 5.0 eq), [Pd_2(dibenzlyideneacetone)$_3$] (4 mg, 30 µmol, 0.03 eq) and PPh_3 (5 mg, 20 µmol, 0.15 eq) in dioxane (10 mL) under nitrogen and the reaction mixture was stirred overnight at 90 °C. DCM (60 mL) was added and the organic layer was washed with water (80 mL). This was purified by chromatography on silica eluting with a gradient of DCM/Acetone 3:1 (v/v). The solid thus obtained was triturated with acetonitrile to give the product as a white powder (9 mg, 7%).

^{1}H NMR (800 MHz, 9:1 $CDCl_3/CD_3CN$, 298 K) δ: 8.85 (1H, s, H_a), 8.26 (1H, d, J = 8.2 Hz, H_d), 8.11 (1H, s, H_c), 8.06 (1H, d, J = 10.6, H_b), 7.81 (1H, s, H_α), 6.46 (2H, s, H_f), 6.41 (1H, s, H_g), 5.50 (2H, s, H_e), 3.75 (6H, s, H_h);
^{13}C NMR (200 MHz, 9:1 $CDCl_3/CD_3CN$, 298 K) δ: 161.4, 149.8, 148.3, 147.9, 139.6, 136.5, 135.5, 135.0, 125.4, 122.3, 120.3, 106.4, 100.6, 55.5, 54.5;
HR ESI-MS ($CDCl_3$/MeOH) m/z = 961.3875 $[MH]^+$ (calc. for $[C_{54}H_{49}N_{12}O_6]^+$, 961.3898);
IR v (cm^{-1}): 3145, 2957, 2840, 1601, 1431, 1208, 1160, 1066, 1042, 846, 757.

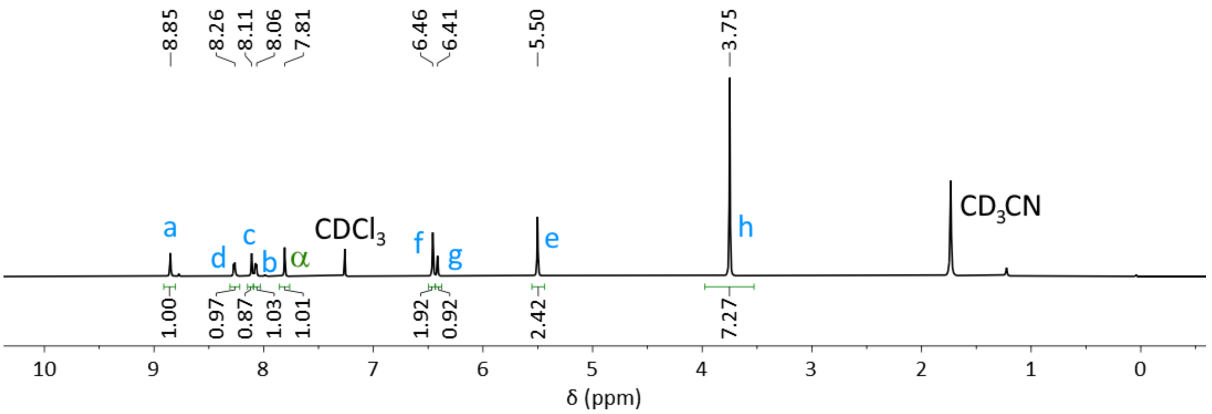

Figure S1.31 ^{1}H NMR spectrum of $\mathbf{L_{111}}$'. (800 MHz, 9:1 $CDCl_3/CD_3CN$, 298 K).

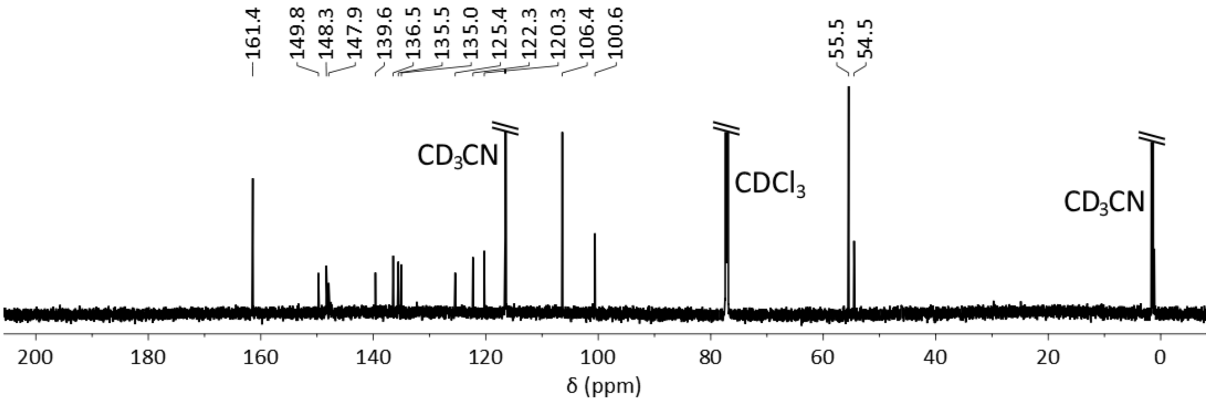

Figure 1.32 ^{13}C NMR spectrum of $\mathbf{L_{111}}$'. (200 MHz, 9:1 $CDCl_3/CD_3CN$, 298 K).

1.4. Complexes

1.4.1. Tet$_{111}$

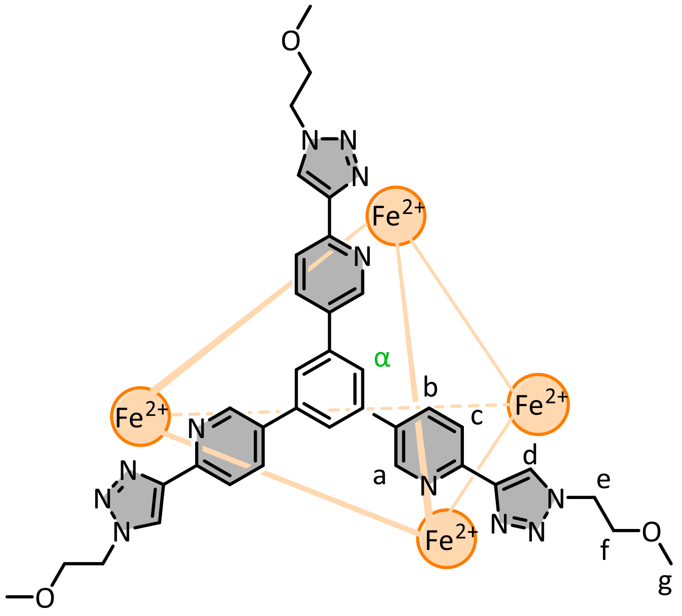

Ligand $\mathbf{L_{111}}$ (2.92 mg, 4.26 µmol, 1.0 eq) was dissolved in an NMR tube in CD_3CN (600 µL). 50 µL of a 85.2 mM stock solution of $[Fe(BF_4)_2]\cdot 6H_2O$ in CD_3CN (4.26 µmol, 1.0 eq) was added and the tube was well shaken to mix. The resulting solution immediately turned bright orange, indicating complexation.

After 15 minutes at room temperature, full equilibration to the $[Fe_4(\mathbf{L_{111}})_4](BF_4)_8$ species was observed by ^{1}H NMR spectroscopy.

^{1}H NMR (400 MHz, CD_3CN, 298 K) δ: 9.00 (br s, 1H, H_d), 8.47 (dd, J = 6.6 Hz, 1H, H_c), 8.31 (d, J = 5.6 Hz, 1H, H_b), 7.59 (br s, 1H, H_a), 7.20 (s, 1H, H_α), 4.65 (d, J = 13.5 Hz, 2H, H_e), 3.81 (br s, 2H, H_f), 3.30 (s, 3H, H_g);
^{1}H DOSY NMR (400 MHz, CD_3CN, 298 K) D ($\times 10^{-10}$ m^2s^{-1}): 3.8;
^{19}F NMR (376 MHz, CD_3CN, 298 K) δ: - 151.5;
HR ESI-MS (CH_3CN) m/z = 370.2455 $[Fe_4L1_4]^{8+}$ (calc. for $[Fe_4C_{144}H_{136}N_{48}O_{12}]^{8+}$, 370.2449); m/z = 435.5634 $[Fe_4L1_4^{8+} + BF_4^-]^{7+}$ (calc. for $[Fe_4C_{144}H_{137}N_{48}O_{12}]^{8+}[BF_4]^-$, 435.5667); m/z = 511.3260 $[Fe_4L1_4^{8+} + BF_4^-+F^-\ ]^{6+}$ (calc. for $[Fe_4C_{144}H_{138}N_{48}O_{12}]^{8+}[BF_4]^-\ [F]^-$, 511.3301); m/z = 522.6649 $[Fe_4L1_4^{8+} + 2BF_4^-]^{6+}$ (calc. for $[Fe_4C_{144}H_{138}N_{48}O_{12}]^{8+}[BF_4]^-_2$, 522.6642); m/z = 644.5986 $[Fe_4L1_4^{8+} + 3BF_4^-]^{5+}$ (calc. for $[Fe_4C_{144}H_{139}N_{48}\ O_{12}]^{8+}[BF_4]^-_3$, 644.5916); m/z = 827.2460 $[Fe_4L1_4^{8+} + 4BF_4^-]^{4+}$ (calc. for $[Fe_4C_{144}H_{140}N_{48}O_{12}]^{8+}[BF_4]^-_4$, 827.2448); m/z = 1131.9919 $[Fe_4L1_4^{8+} + 5BF_4^-]^{3+}$ (calc. for $[Fe_4C_{144}H_{141}N_{48}O_{12}]^{8+}[BF_4]^-_5$, 1131.9916)

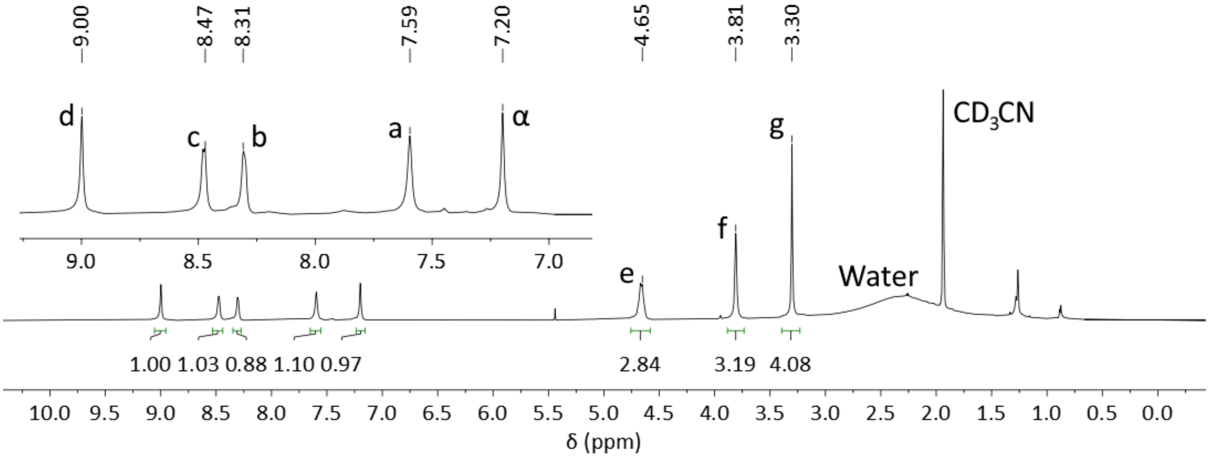

Figure 1.33: ^{1}H NMR spectrum of **Tet$_{111}$** (400 MHz, CD_3CN, 298 K).

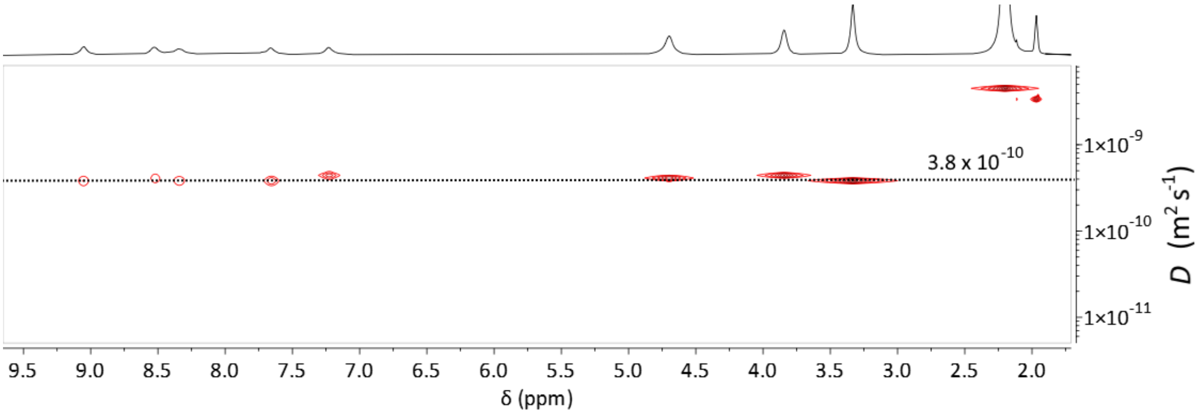

Figure S1.34: ^{1}H DOSY spectrum of **Tet$_{111}$** (400 MHz, CD_3CN, 298 K).

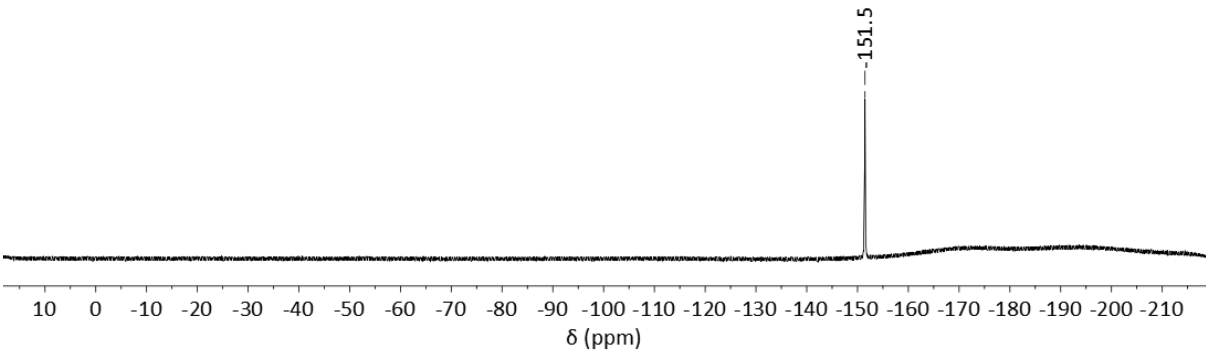

Figure S1.35: ^{19}F NMR spectrum of **Tet$_{111}$** (376 MHz, CD_3CN, 298 K).

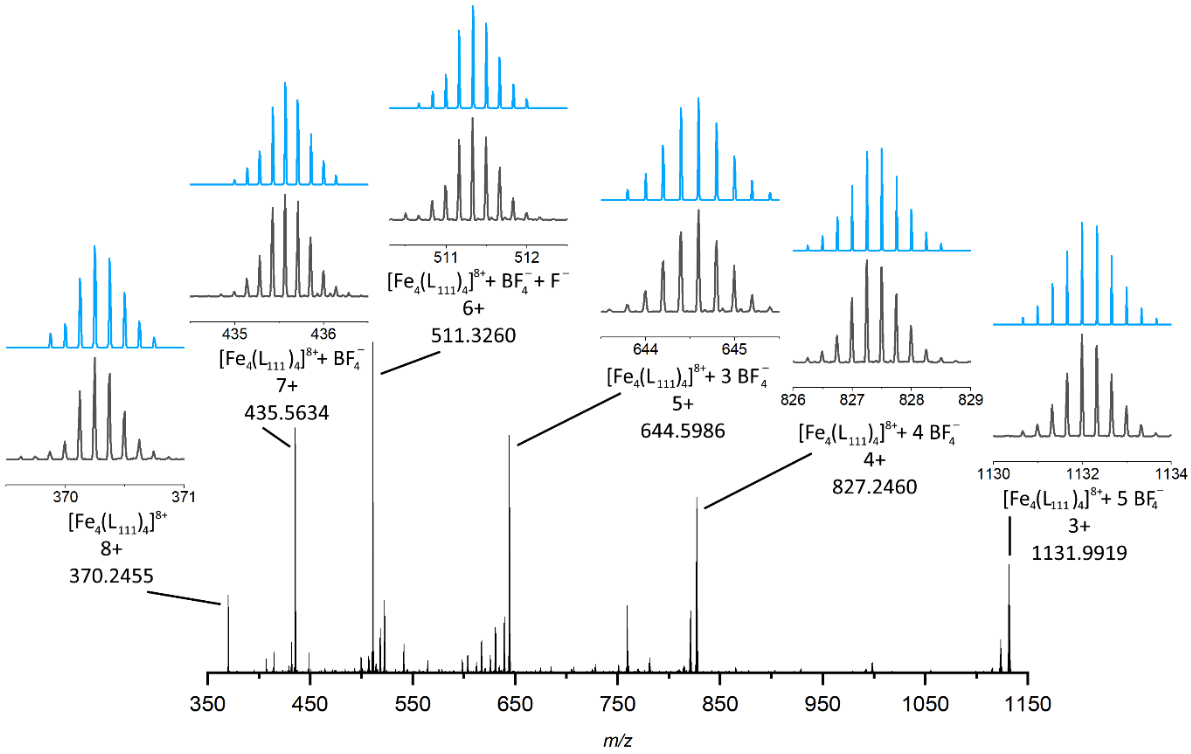

Figure S1.36: HR-ESI mass spectrum of **Tet$_{111}$** (CD_3CN/CH_3CN).

CCDC#: 2414211. Vapour diffusion of diethyl ether into a solution of **Tet$_{111}$** in acetonitrile gave red crystals of **Tet$_{111}$**· ([Fe_4(**L$_{111}$**)$_4$](BF_4)$_8$). X-ray data were collected at 100 K on the MX2 beamline[4] at the Australian Synchrotron, and data were treated using XDS[5] software. The structure was solved using SHELXT[6] within OLEX2 and weighted full-matrix refinement on F^2 was carried out using SHELXL-97 running within the OLEX2[7] package. Non-hydrogen atoms were refined anisotropically, except for those in solubilising groups that were too unstable, which were left isotropic. Hydrogen atoms attached to carbons were placed in calculated positions and refined using a riding model. The structure was solved in the monoclinic space group $P2_1/n$ and refined to an R_1 value of 12.47 %. The final refinement of the asymmetric unit contained two tetrahedra and ten tetrafluoroborate counterions. Six remaining tetrafluoroborate counterions as needed for charge balance, along with various diffuse solvent molecules, were squeezed out and thus not observed in the final refinement.

Many of the polyether chains were highly disordered and this disorder could not be refined to an appropriate model. This necessitated the truncation of the polyether chains in some cases. Anisotropic refinement models were initially developed, but these were partially taken back to mixed isotropic/anisotropic handling, based on the ADP behaviours guided by the poorly defined electron density maps. Disorder that could be appropriately modelled required the use of various geometry and ADP restraints. There was diffuse electron density within the lattice that could not be appropriately modelled (attributed to solvent, missing tetrafluoroborate anions and disorder in the polyether chains). This required the use of Solvent Mask, with a calculated void per asymmetric unit of 721 electrons and 3833 Å^3.

The crystals that were grown were of poor quality and prone to desolvation. The resulting data is thus quite poor, particularly with regards to the polyether chains. The final refinement is stable, however, and the connectivity of the main tetrahedral framework is still observed and consistent with other data. This was the best data crystal obtained after screening multiple from the same sample.

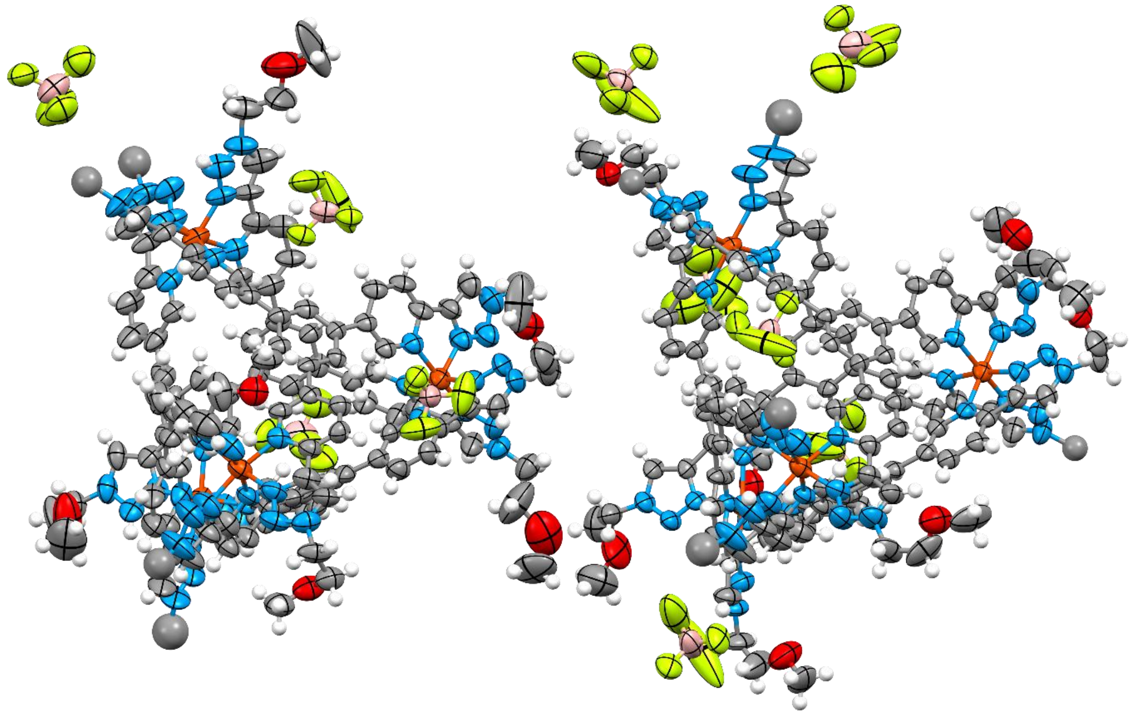

Figure 1.37 Mercury ellipsoid plot of the asymmetric unit of **Tet$_{111}$**. Ellipsoids shown at 50% probability level. Colour scheme: carbon grey, hydrogen white, boron salmon, fluorine lime, nitrogen blue, oxygen red, iron orange.

Empirical formula	$C_{264}H_{207}B_{10}F_{40}Fe_8N_{96}O_{12}$
Formula weight	6231.14
Temperature/K	100(2)
Crystal system	monoclinic
Space group	$P2_1/n$
a/Å	30.498(6)
b/Å	33.663(7)
c/Å	37.841(8)
α/°	90
β/°	112.62(3)
γ/°	90
Volume/Å^3	35862(14)
Z	4
ρ_{calc}g/cm^3	1.154
μ/mm^{-1}	0.396
F(000)	12708.0
Crystal size/mm^3	0.08 × 0.07 × 0.03
Radiation/Å	Synchrotron (λ = 0.71073)
2Θ range for data collection/°	1.468 to 43.948
Index ranges	-32 ≤ h ≤ 32, -35 ≤ k ≤ 35, -39 ≤ l ≤ 39
Reflections collected	291020
Independent reflections	41894 [R_{int} = 0.0578, R_{sigma} = 0.0303]
Data/restraints/parameters	41894/115/3814
Goodness-of-fit on F^2	1.560
Final R indexes [I>=2σ (I)]	R_1 = 0.1247, wR_2 = 0.3575
Final R indexes [all data]	R_1 = 0.1454, wR_2 = 0.3800
Largest diff. peak/hole / e Å^{-3}	1.89/-0.65

1.4.2. Tet$_{112}$

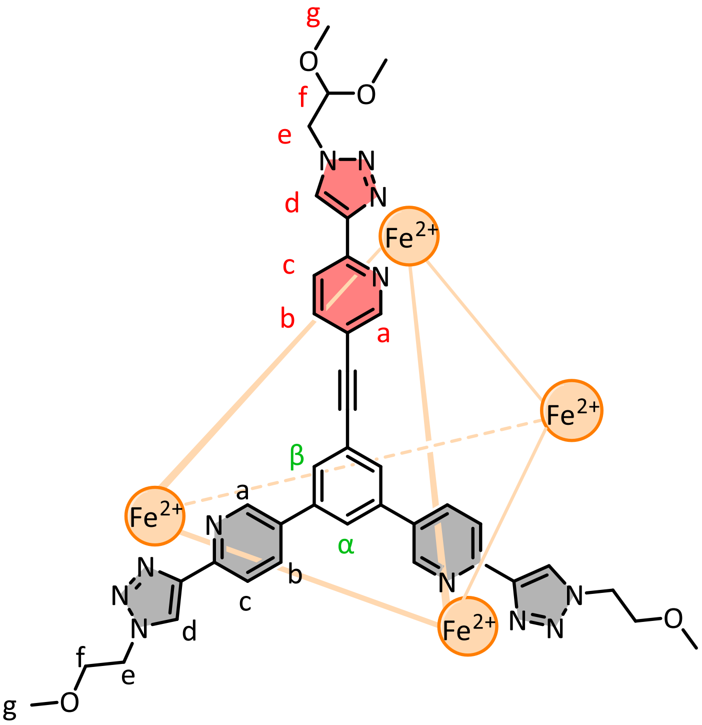

Ligand **L$_{112}$** (2.67 mg, 3.61 µmol, 1.0 eq) was dissolved in an NMR tube in CD_3CN (600 µL). 50 µL of a 72.3 mM stock solution of $[Fe(BF_4)_2]\cdot 6H_2O$ in CD_3CN (3.61 µmol, 1.0 eq) was added and the tube was

well shaken to mix. The resulting solution immediately turned bright orange, indicating complexation. After 15 minutes at room temperature, equilibration to the $[Fe_4(\mathbf{L_{112}})_4](BF_4)_8$ species was observed by ^{1}H NMR spectroscopy. The solution was heated overnight at 50 °C to ensure full equilibration.

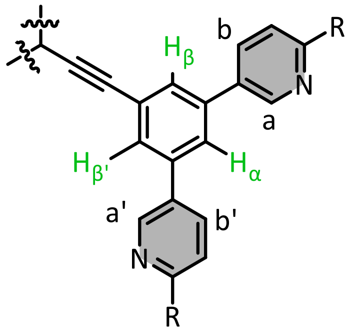

Figure 1.38: The proton environments around the central aromatic ring as assigned in the below spectra.

^{1}H NMR (800 MHz, CD_3CN, 298 K) δ: 9.04 (s, 1H, H_d), 9.01 (s, 1H, $H_{d'}$), 8.93 (s, 1H, H_d), 8.56 (br s, 1H, H_c), 8.48 (m, 2H, $H_{c,c'}$), 8.30-8.24 (m, 3H, $H_{b,b,b'}$), 8.08 (br s, 1H, $H_{a'}$), 7.94 (br s, 1H, H_a), 7.86 (br s, 1H, H_a), 7.67 (br s, 1H, H_β), 7.22 (br s, 1H, H_α), 7.12 (br s, 1H, $H_{\beta'}$), 4.67 (m, 2H, H_f), 4.64 (m, 4H, H_e), 4.57 (m, 2H, H_e), 3.81 (m, 4H, H_f), 3.32 (s, 6H, H_g), 3.27 (s, 3H, H_g);
^{1}H DOSY NMR (400 MHz, CD_3CN, 298 K) D ($\times 10^{-10}$ m^2s^{-1}): 5.1;
^{19}F NMR (376 MHz, CD_3CN, 298 K) δ: -151.3;
HR ESI-MS (CH_3CN) m/z = 687.8042 $[Fe_4(L_{112})_4{}^{8+} + 3\ BF_4{}^-]^{5+}$ (calc. for $[Fe_4C_{156}H_{152}N_{48}O_{16}B_3F_{12}]^{5+}$, 687.8016); m/z = 881.5064 $[Fe_4(L_{112})_4{}^{8+} + 4\ BF_4{}^-]^{4+}$ (calc. for $[Fe_4C_{156}H_{152}N_{48}O_{16}B_4F_{16}]^{4+}$, 881.5032); m/z = 1204.3438 $[Fe_4(L_{112})_4{}^{8+} + 5\ BF_4{}^-]^{3+}$ (calc. for $[Fe_4C_{156}H_{152}N_{48}O_{16}B_5F_{20}]^{3+}$, 1204.3390)

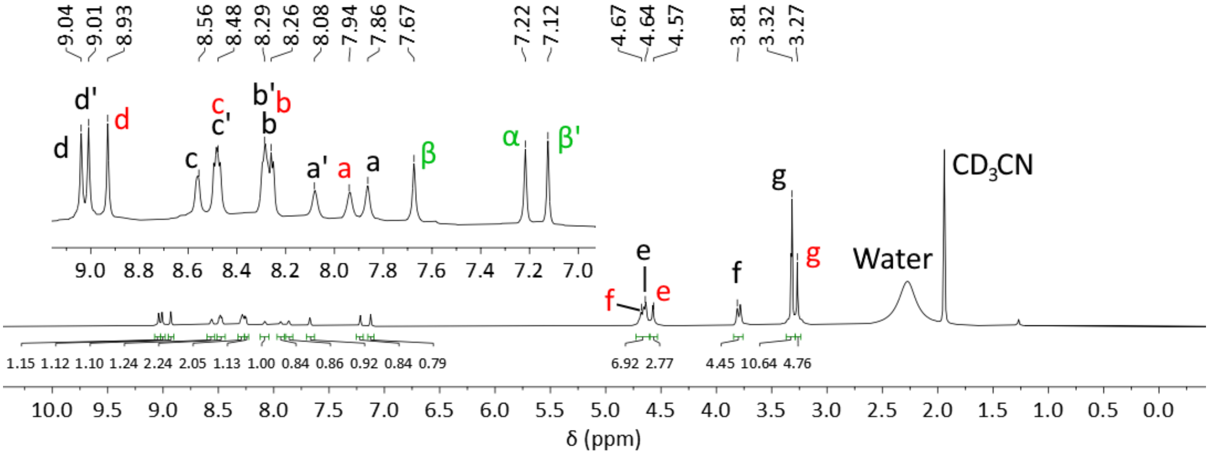

Figure S1.39: ^{1}H NMR spectrum of $\mathbf{Tet_{112}}$ (800 MHz, CD_3CN, 298 K).

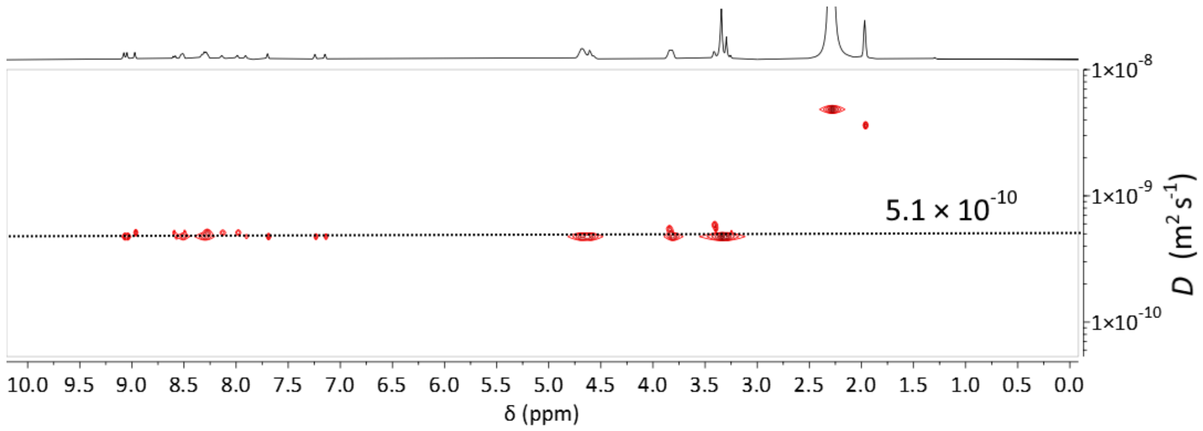

Figure S1.40: ^{1}H DOSY NMR spectrum of **Tet$_{112}$** (400 MHz, CD_3CN, 298 K).

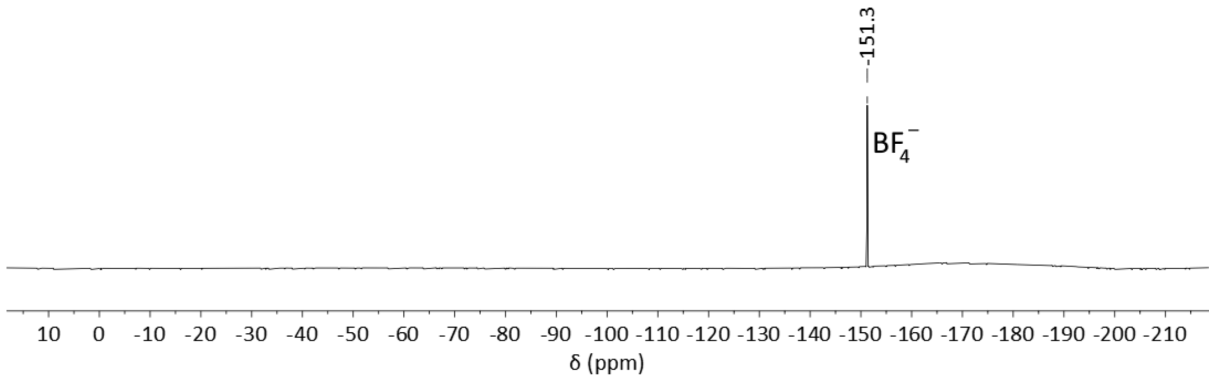

Figure S1.41: ^{19}F NMR spectrum of **Tet$_{112}$** (376 MHz, CD_3CN, 298 K).

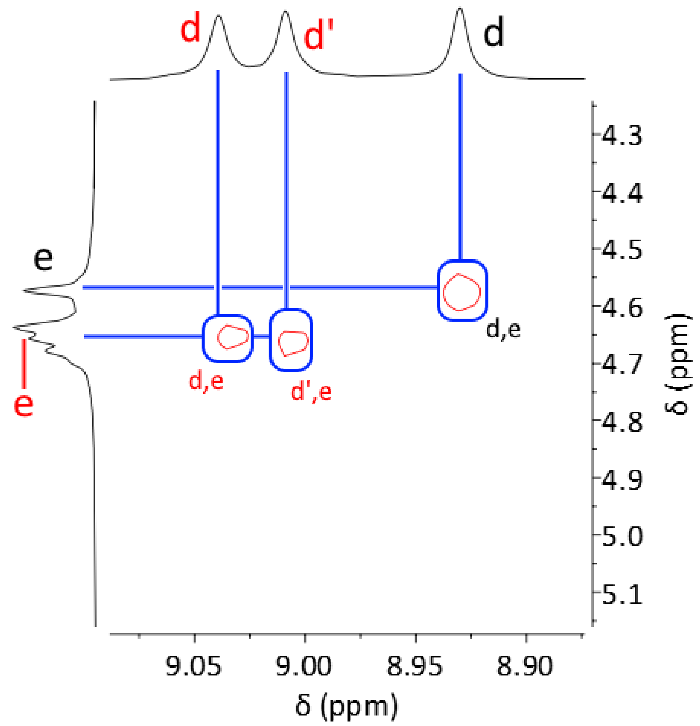

Figure S1.42: Partial ^{1}H NOESY 2D NMR spectrum of **Tet$_{112}$** (800 MHz, CD_3CN, 298 K, 200 ms).

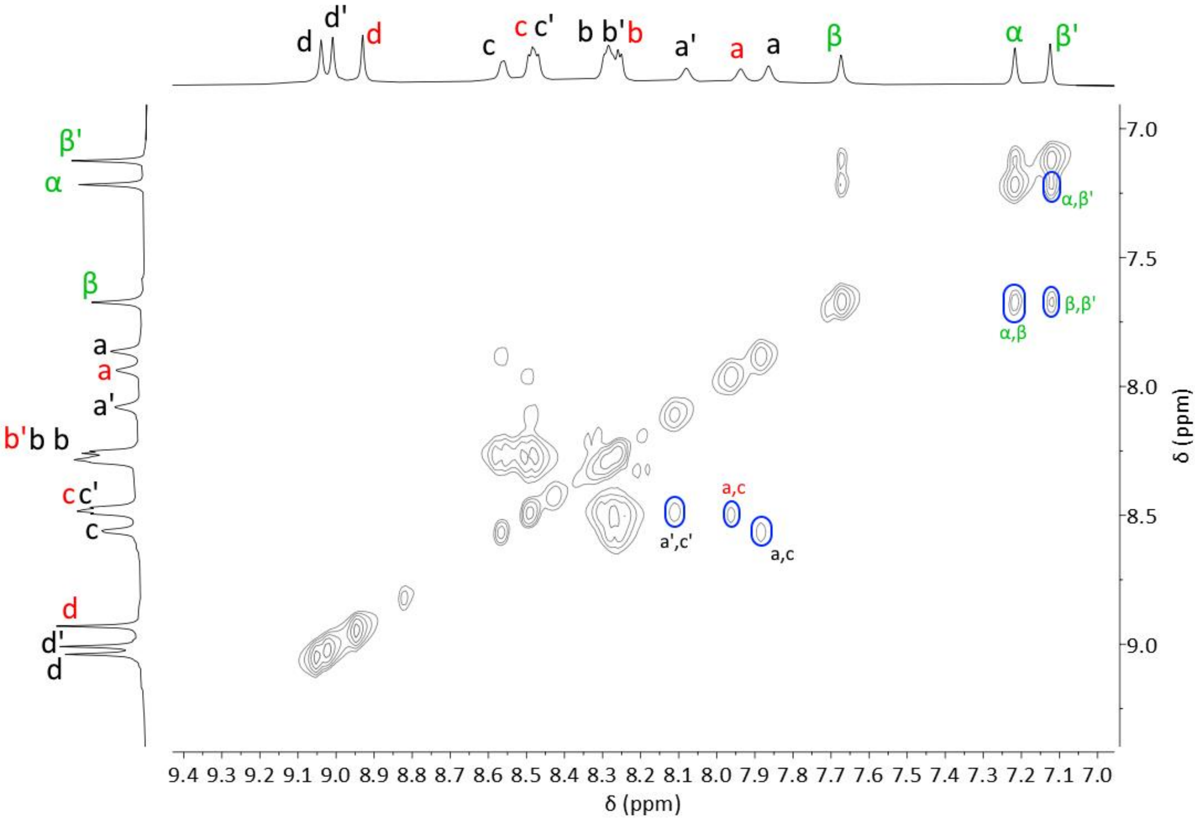

Figure S1.43: Partial ^{1}H NOESY 2D NMR spectrum of **Tet$_{112}$** (800 MHz, CD_3CN, 298 K, 200 ms).

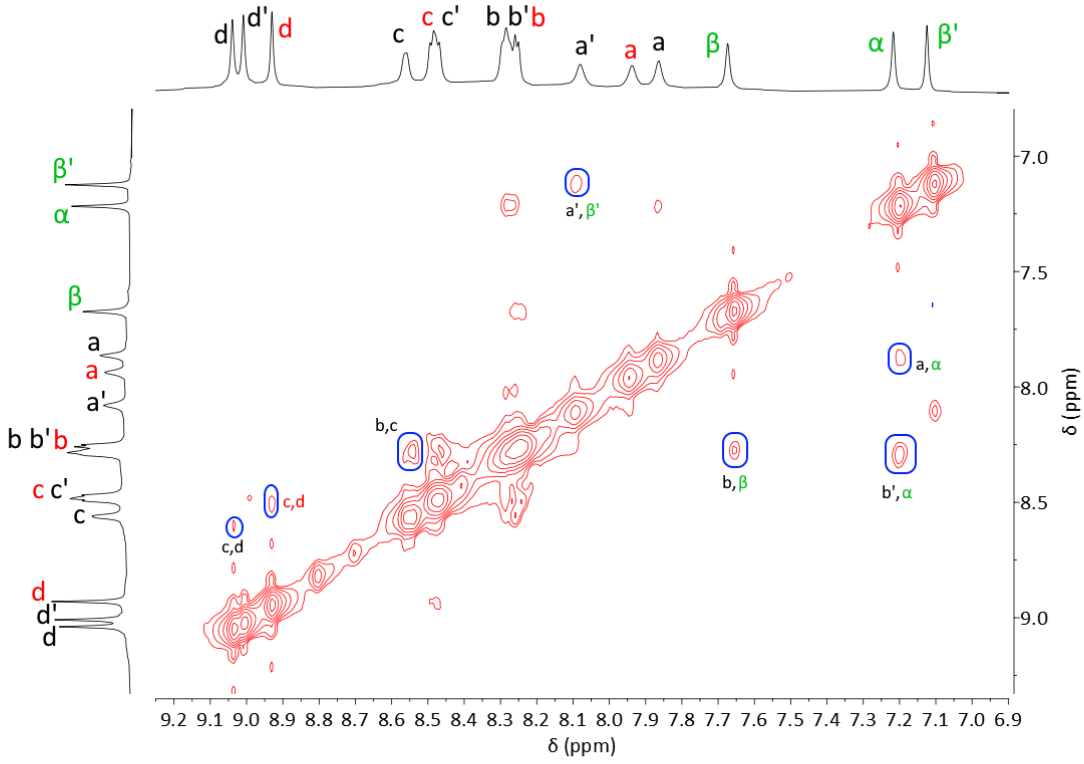

Figure S1.44: Partial ^{1}H NOESY 2D NMR spectrum of **Tet$_{112}$** (800 MHz, CD_3CN, 298 K, 200 ms).

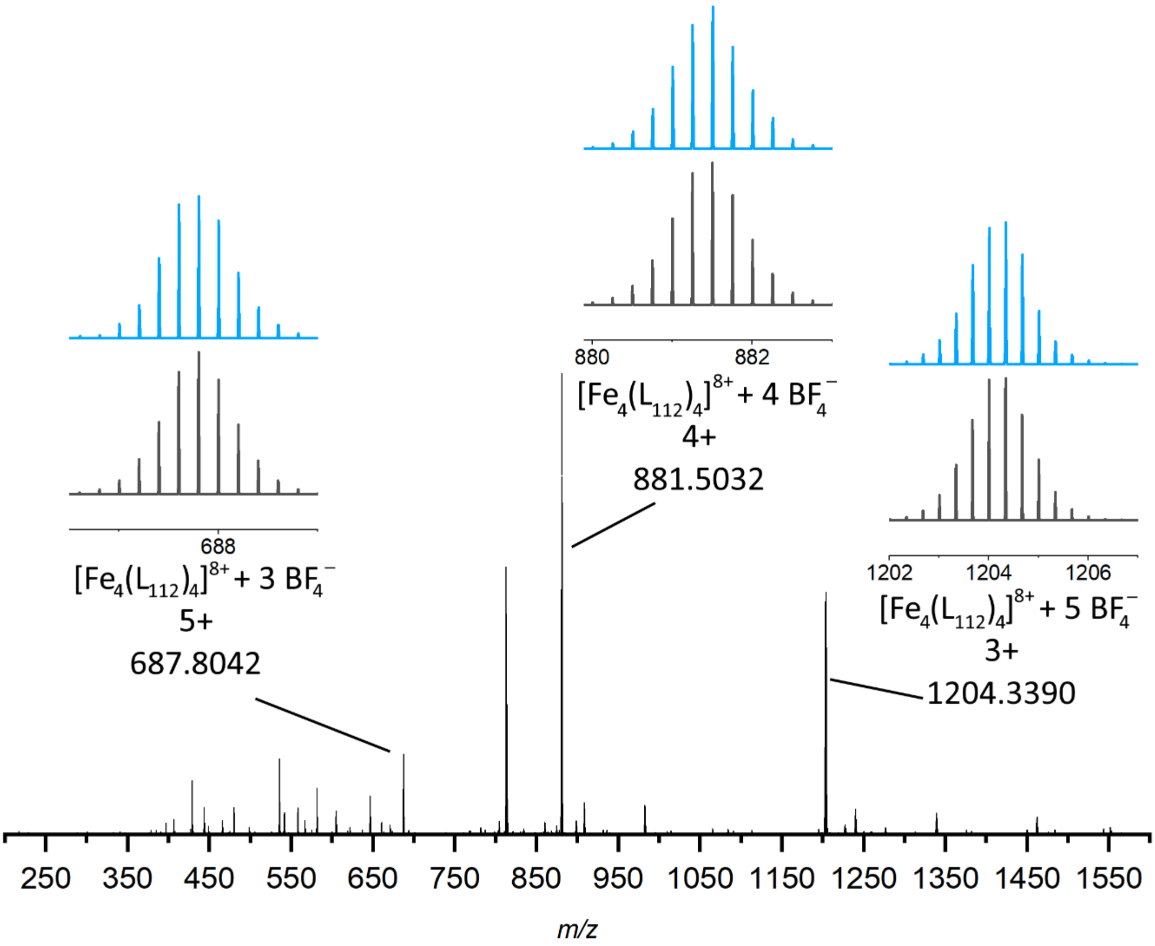

Figure S1.45: HR-ESI mass spectrum of **Tet_{112}** (CD_3CN/CH_3CN).

CCDC#: 2414212. Vapour diffusion of diethyl ether into a solution of **Tet_{112}** in acetonitrile gave red crystals of **Tet_{112}**· ([Fe_4(**L_{112}**)$_4$](BF_4)$_8$). X-ray data were collected at 100 K on the MX2 beamline[4] at the Australian Synchrotron, and data were treated using CX-ASAP[8] software. The structure was solved using SHELXT[6] within OLEX2 and weighted full-matrix refinement on F^2 was carried out using SHELXL-97 running within the OLEX2[7] package. Non-hydrogen atoms were refined anisotropically, except for those in solubilising groups that were too unstable, which were left isotropic. Hydrogen atoms attached to carbons were placed in calculated positions and refined using a riding model. The structure was solved in the triclinic space group *P*-1 and refined to an R_1 value of 20.56 %. The asymmetric unit contained two tetrahedra and four tetrafluoroborate counterions. Twelve remaining tetrafluoroborate counterions as needed for charge balance, along with various diffuse solvent molecules, were squeezed out and thus not observed in the final refinement.

Many of the polyether chains were highly disordered and this disorder could not be refined to an appropriate model. This necessitated the truncation of the polyether chains in some cases. Anisotropic refinement models were initially developed, but these were partially taken back to mixed isotropic/anisotropic handling, based on the ADP behaviours guided by the poorly defined electron density maps. Disorder that could be appropriately modelled required the use of various geometry and ADP restraints. There was diffuse electron density within the lattice that could not be appropriately modelled (attributed to solvent, missing tetrafluoroborate anions and disorder in the polyether chains). This required the use of Solvent Mask, with a calculated void per asymmetric unit of 1582 electrons and 6032 Å^3.

The crystals that were grown were of poor quality and prone to desolvation. The resulting data is thus quite poor, particularly with regards to the polyether chains. The final refinement is stable, however, and the connectivity of the main tetrahedral framework is still observed and consistent with other data. This was the best data crystal obtained after screening multiple from the same sample.

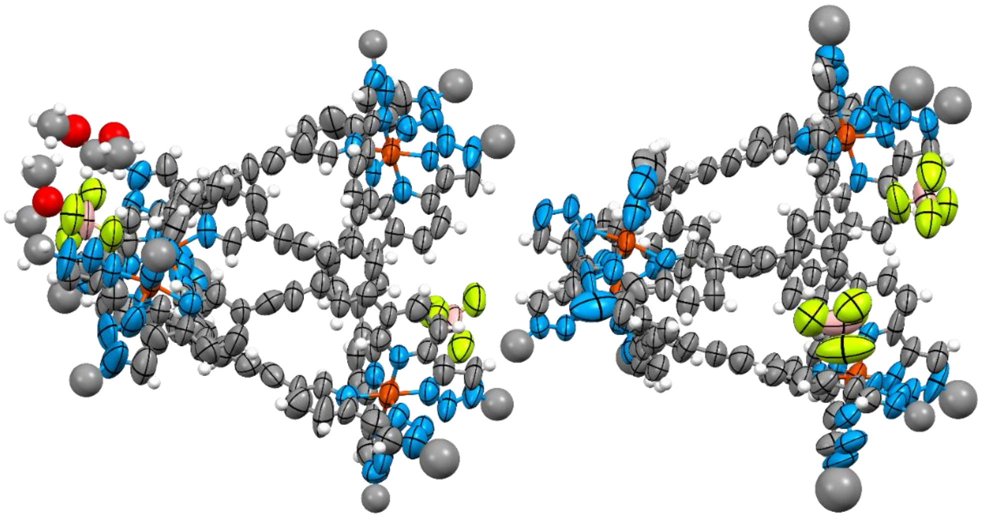

Figure S1.46 Mercury ellipsoid plot of the asymmetric unit of **Tet$_{112}$**. Ellipsoids shown at 50% probability level. Colour scheme: carbon grey, hydrogen white, boron salmon, fluorine lime, nitrogen blue, oxygen red, iron orange.

Empirical formula	$C_{257}H_{137}B_4F_{16}Fe_8N_{96}O_3$
Formula weight	5411.66
Temperature/K	293(2)
Crystal system	triclinic
Space group	*P*-1
a/Å	27.206(5)
b/Å	30.058(6)
c/Å	32.002(6)
α/°	87.26(3)
β/°	70.33(3)
γ/°	63.41(3)
Volume/Å^3	21870(10)
Z	2
ρ_{calc}g/cm^3	0.822
μ/mm^{-1}	0.309
F(000)	5494.0
Crystal size/mm^3	0.15 × 0.08 × 0.05
Radiation/Å	Synchrotron (λ = 0.71073)
2Θ range for data collection/°	1.362 to 41.63
Index ranges	-27 ≤ h ≤ 27, -30 ≤ k ≤ 30, -31 ≤ l ≤ 31
Reflections collected	159937
Independent reflections	43821 [R_{int} = 0.0318, R_{sigma} = 0.0372]
Data/restraints/parameters	43821/3027/3236
Goodness-of-fit on F^2	2.105
Final R indexes [I>=2σ (I)]	R_1 = 0.2056, wR_2 = 0.5074
Final R indexes [all data]	R_1 = 0.2368, wR_2 = 0.5418
Largest diff. peak/hole / e Å^{-3}	1.81/-0.78

1.4.3. Tet_{122}

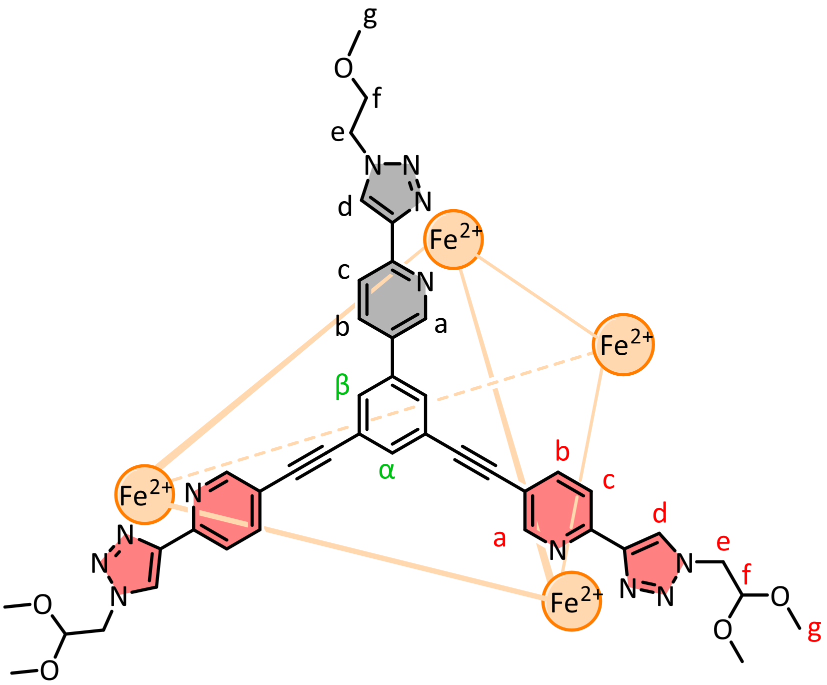

Ligand $\mathbf{L_{122}}$ (3.15 mg, 3.97 µmol, 1.0 eq) was dissolved in an NMR tube in CD_3CN (600 µL). 50 µL of a 79.5 mM stock solution of $[Fe(BF_4)_2]\cdot 6H_2O$ in CD_3CN (3.97 µmol, 1.0 eq) was added and the tube was well shaken to mix. The resulting solution immediately turned bright orange, indicating complexation. After 15 minutes at room temperature, equilibration to the $[Fe_4(\mathbf{L_{122}})_4](BF_4)_8$ species was observed by ^1H NMR spectroscopy. The solution was heated overnight at 50 °C to ensure full equilibration.

^1H NMR (800 MHz, CD_3CN, 298 K) δ: 9.01 (s, 1H, H_d), 8.97 (s, 1H, H_{d*}), 8.91 (s, 1H, H_{d*}), 8.52 (m, 2H, $H_{c,c*}$), 8.45 (d, J = 8.0 Hz, 1H, H_{c*}), 8.32 (m, 2H, $H_{a,b}$), 8.26 (m, 3H, $H_{a,a',b*}$), 8.17 (d, J = 8.1 Hz, 1H, H_{b*}), 7.72 (s, 1H, $H_{\beta*}$), 7.50 (s, 1H, H_α), 7.14 (s, 1H, $H_{\beta*}$), 4.64 (m, 2H, H_f), 4.61 (m, 2H, H_e), 4.56 (m, 4H, H_e), 3.76 (t, J = 4.9 Hz, 2H, H_f), 3.31 (s, 12H, H_g), 3.26 (s, 3H, H_g);

^1H DOSY NMR (400 MHz, CD_3CN, 298 K) D ($\times 10^{-10}$ m^2s^{-1}): 6.2;

^{19}F NMR (376 MHz, CD_3CN, 298 K) δ: -151.4;

HR ESI-MS (CH_3CN) m/z = 594.6776 $[Fe_4(L_{122})_4^{8+} + 2\ BF_4^-]^{6+}$ (calc. for $[Fe_4C_{168}H_{160}N_{48}O_{20}B_2F_8]^{6+}$, 594.6744); m/z = 731.0140 $[Fe_4(L_{122})_4^{8+} + 3\ BF_4^-]^{5+}$ (calc. for $[Fe_4C_{168}H_{160}N_{48}O_{20}B_3F_{12}]^{5+}$, 731.0101); m/z = 935.5184 $[Fe_4(L_{122})_4^{8+} + 4\ BF_4^-]^{4+}$ (calc. for $[Fe_4C_{168}H_{160}N_{48}O_{20}B_4F_{16}]^{4+}$, 935.5138); m/z = 1276.3606 $[Fe_4(L_{122})_4^{8+} + 5\ BF_4^-]^{3+}$ (calc. for $[Fe_4C_{168}H_{160}N_{48}O_{20}B_5F_{20}]^{3+}$, 1276.3530)

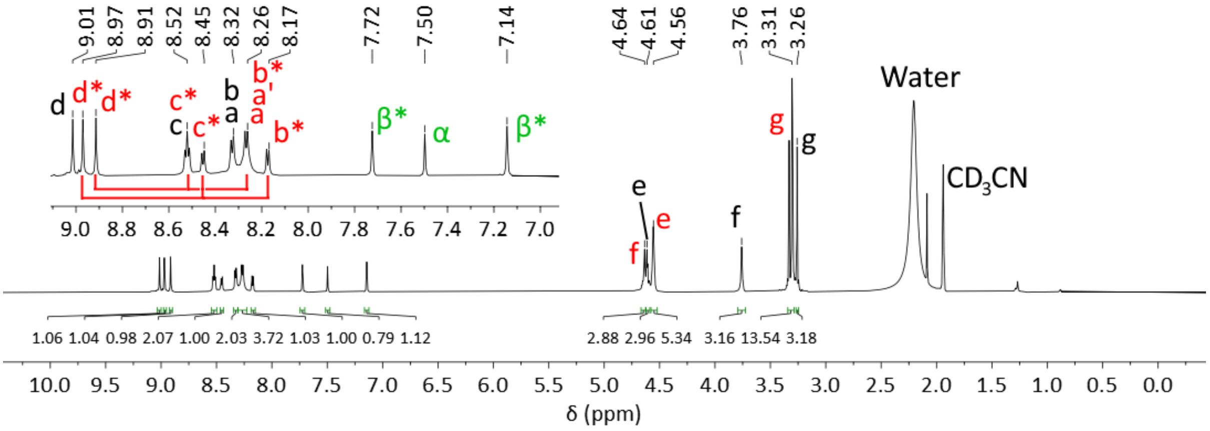

Figure S1.47: ^1H NMR spectrum of $\mathbf{Tet_{122}}$ (800 MHz, CD_3CN, 298 K).

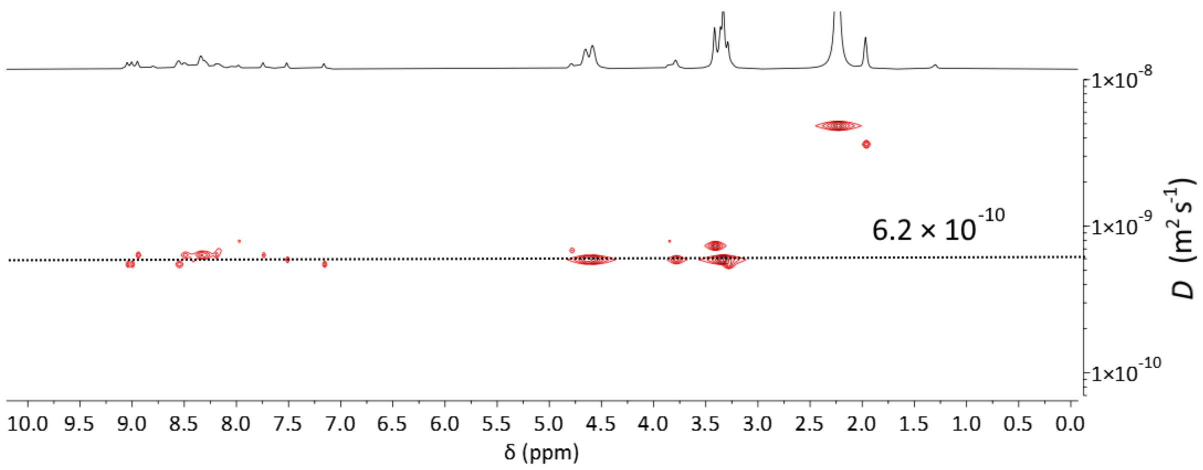

Figure S1.48: ^{1}H DOSY NMR spectrum of **Tet$_{122}$** (400 MHz, CD_3CN, 298 K).

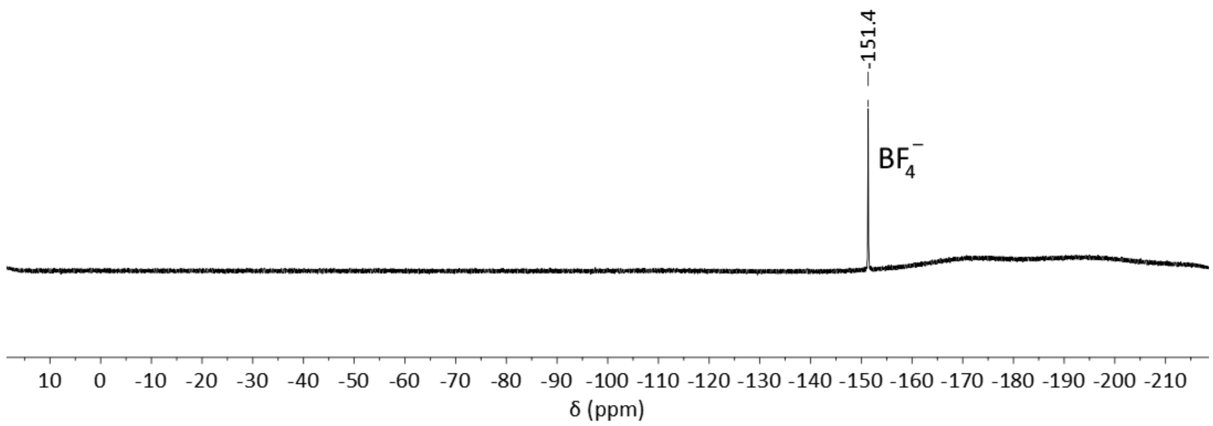

Figure 1.49: ^{19}F NMR spectrum of **Tet$_{122}$** (376 MHz, CD_3CN, 298 K).

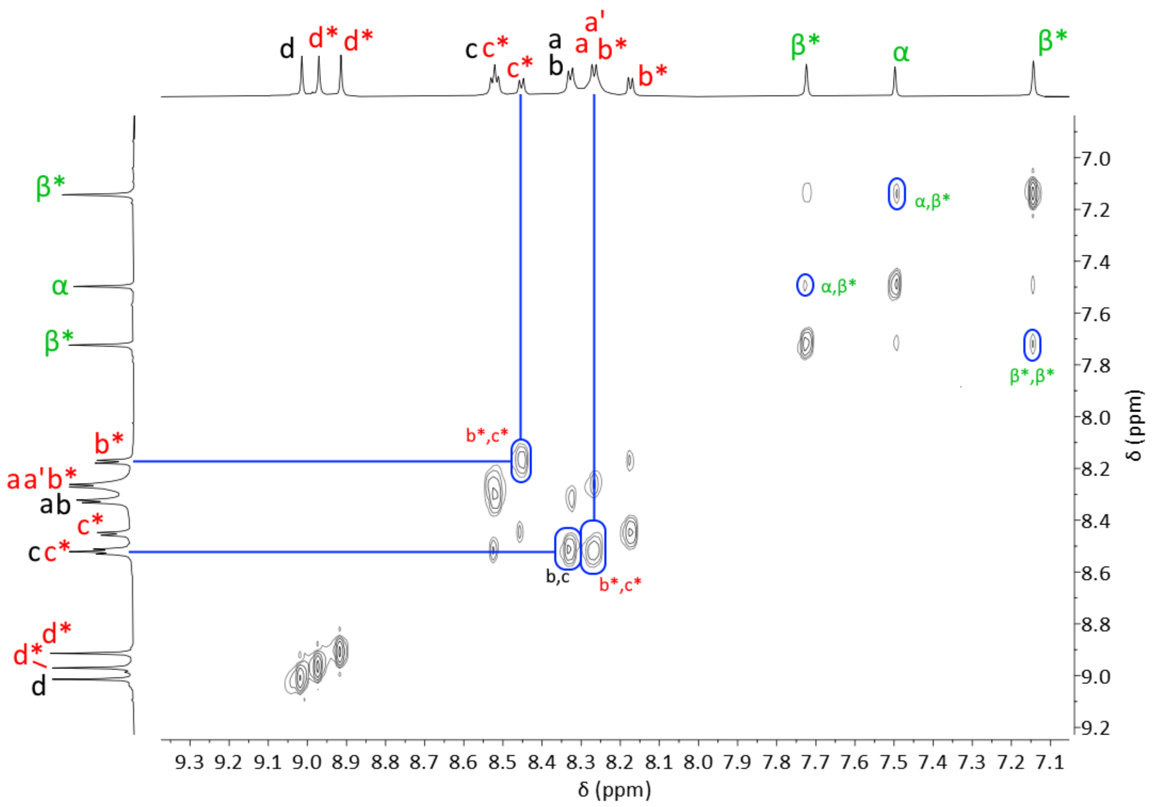

Figure S1.50: Partial ^{1}H TOCSY 2D NMR spectrum of **Tet$_{122}$** (800 MHz, CD_3CN, 298 K, 200 ms).

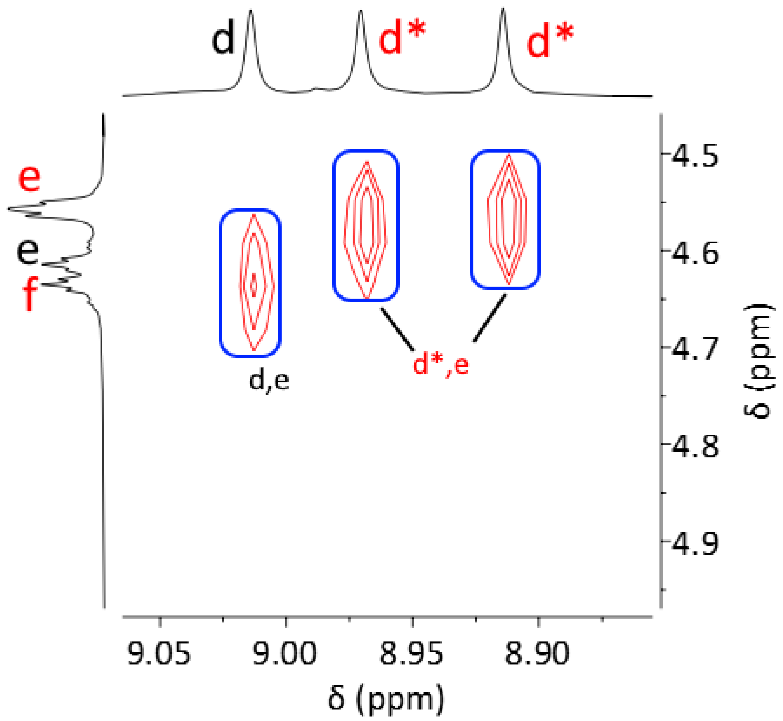

Figure S1.51: Partial ^{1}H NOESY 2D NMR spectrum of **Tet$_{122}$** (800 MHz, CD_3CN, 298 K, 200 ms).

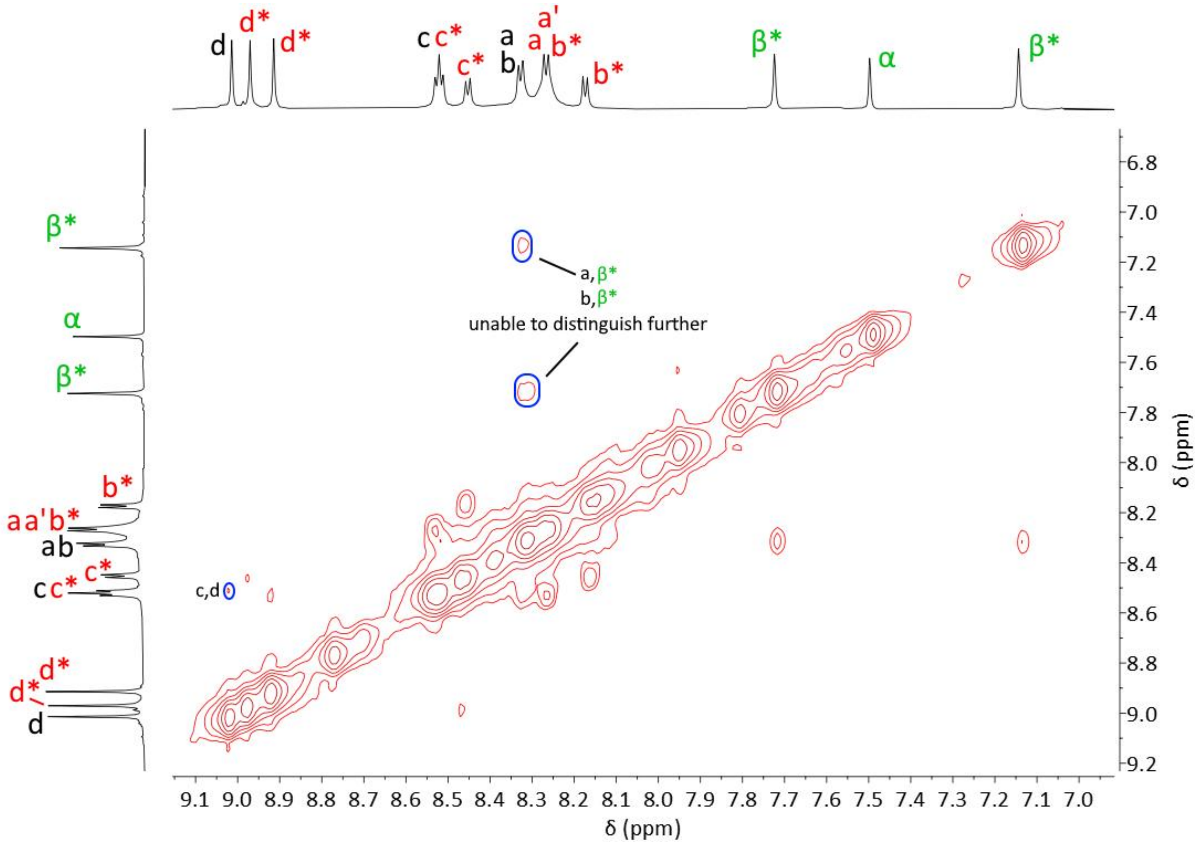

Figure S1.52: Partial ^{1}H NOESY 2D NMR spectrum of **Tet$_{122}$** (800 MHz, CD_3CN, 298 K, 200 ms).

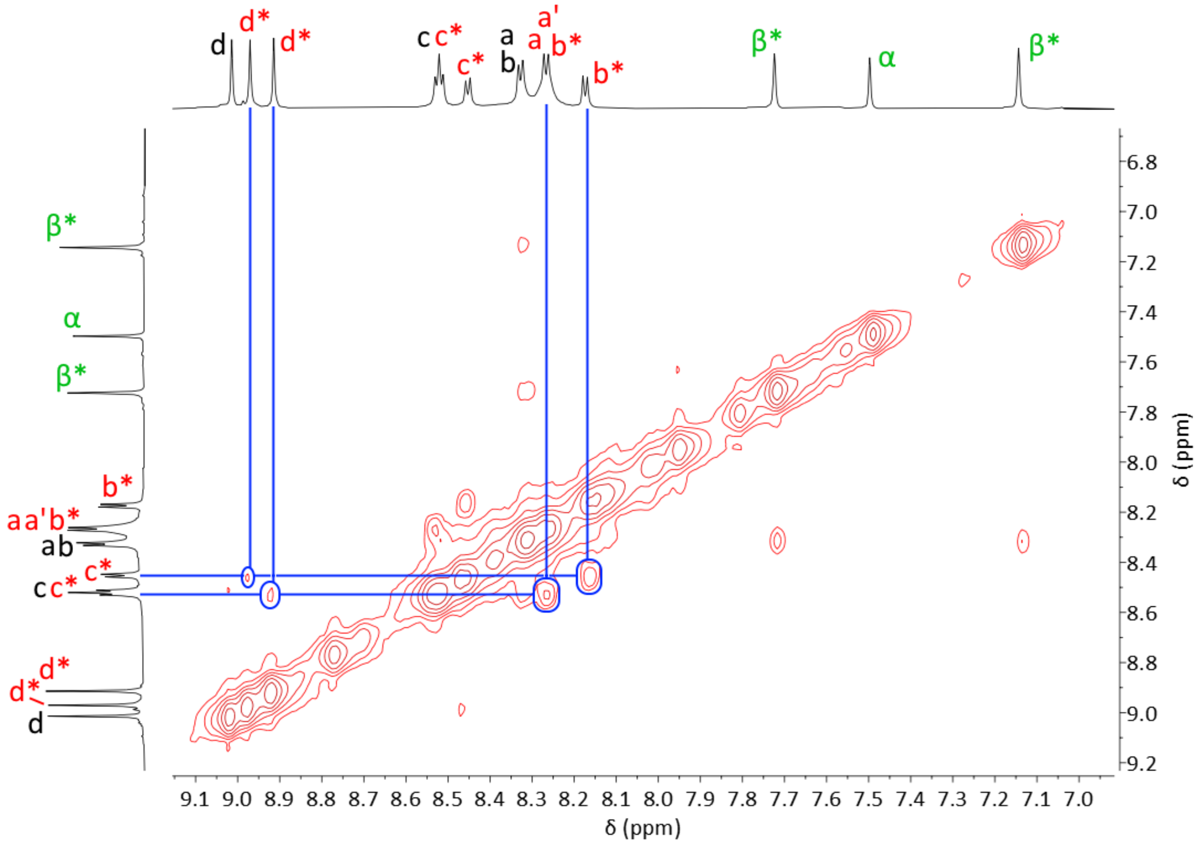

Figure S1.53: Partial ^{1}H NOESY 2D NMR spectrum of $\textbf{Tet}_{122}$ (800 MHz, CD_3CN, 298 K, 200 ms)

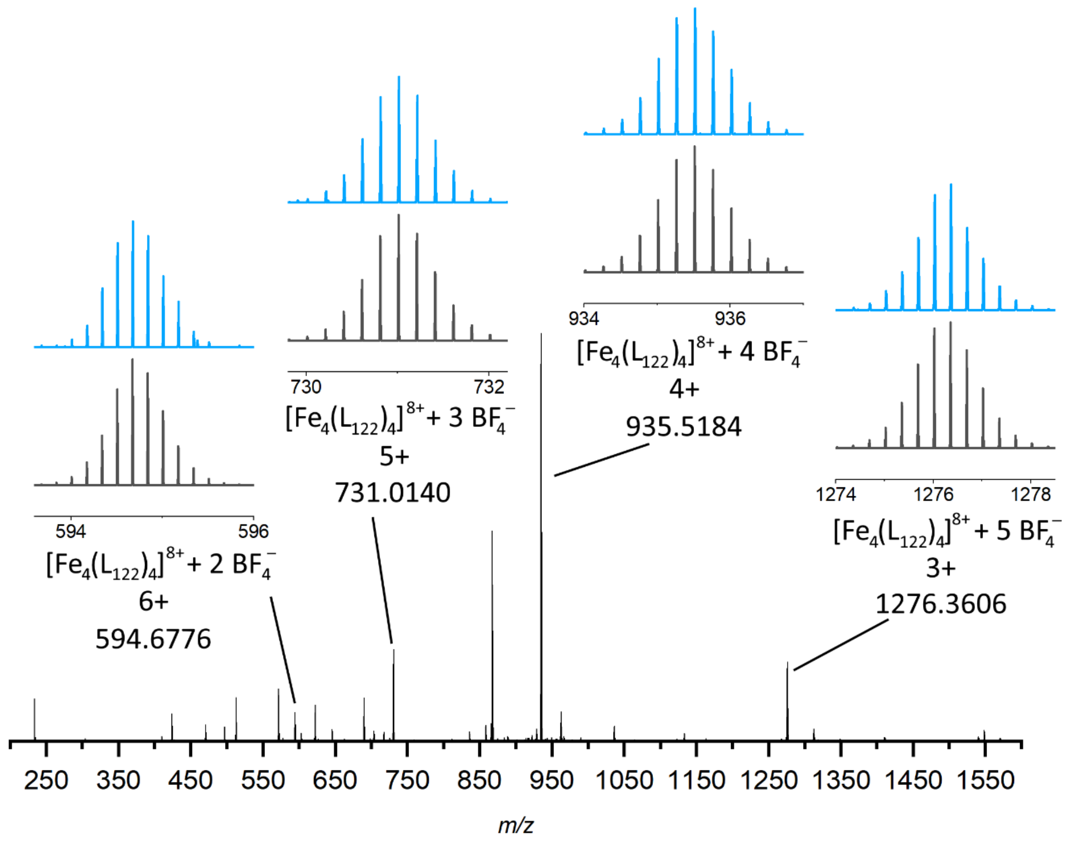

Figure S1.54: HR-ESI mass spectrum of $\textbf{Tet}_{112}$ (CD_3CN/CH_3CN).

CCDC#: 2414213. Vapour diffusion of diethyl ether into a solution of $\mathbf{Tet_{122}}$ in acetonitrile gave red crystals of $\mathbf{Tet_{122}}$· ([Fe_4($\mathbf{L_{122}}$)$_4$](BF_4)$_8$). X-ray data were collected at 100 K on the MX2 beamline[4] at the Australian Synchrotron, and data were treated using CX-ASAP[8] software. The structure was solved using SHELXT[6] within OLEX2 and weighted full-matrix refinement on F^2 was carried out using SHELXL-97 running within the OLEX2[7] package. Non-hydrogen atoms were refined anisotropically, except for those in solubilising groups that were too unstable, which were left isotropic. Hydrogen atoms attached to carbons were placed in calculated positions and refined using a riding model. The structure was solved in the triclinic space group *P-1* and refined to an R_1 value of 15.72 %. The asymmetric unit contained one tetrahedron. Eighty tetrafluoroborate counterions as needed for charge balance, along with various diffuse solvent molecules, were squeezed out and thus not observed in the final refinement.

Many of the polyether chains were highly disordered and this disorder could not be refined to an appropriate model. This necessitated the truncation of the polyether chains in some cases. Anisotropic refinement models were initially developed, but these were partially taken back to mixed isotropic/anisotropic handling, based on the ADP behaviours guided by the poorly defined electron density maps. Disorder that could be appropriately modelled required the use of various geometry and ADP restraints. There was diffuse electron density within the lattice that could not be appropriately modelled (attributed to solvent, missing tetrafluoroborate anions and disorder in the polyether chains). This required the use of Solvent Mask, with a calculated void per asymmetric unit of 770 electrons and 2788 Å^3.

The crystals that were grown were of poor quality and prone to desolvation. The resulting data is thus quite poor, particularly with regards to the polyether chains. The final refinement is stable, however, and the connectivity of the main tetrahedral framework is still observed and consistent with other data. This was the best data crystal obtained after screening multiple from the same sample.

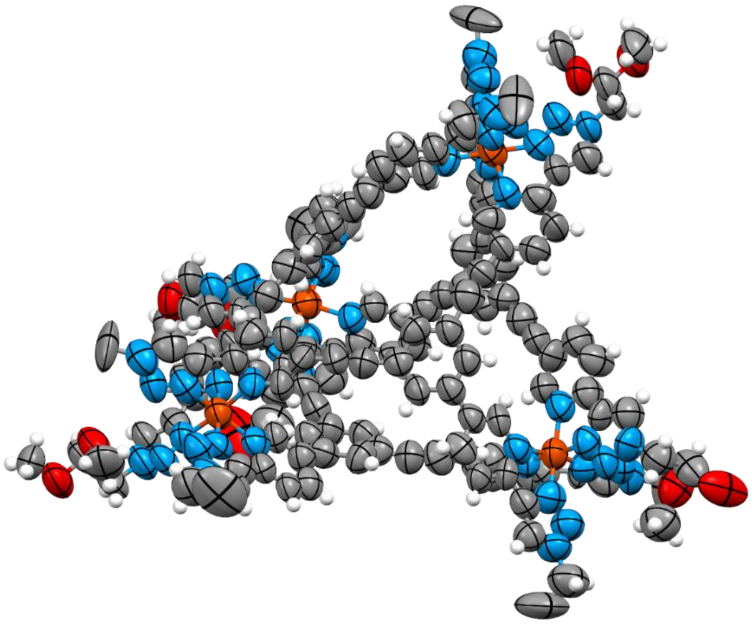

Figure S1.55 Mercury ellipsoid plot of the asymmetric unit of $\mathbf{Tet_{122}}$. Ellipsoids shown at 50% probability level. Colour scheme: carbon grey, hydrogen white, boron salmon, fluorine lime, nitrogen blue, oxygen red, iron orange.

Empirical formula	$C_{151}H_{102}Fe_4N_{48}O_9$
Formula weight	2956.20
Temperature/K	293(2)
Crystal system	triclinic
Space group	*P*-1
a/Å	21.034(4)
b/Å	23.639(5)
c/Å	23.656(5)
α/°	76.54(3)
β/°	80.31(3)
γ/°	80.47(3)
Volume/Å^3	11177(4)
Z	2
ρ_{calc}g/cm^3	0.878
μ/mm^{-1}	0.304
F(000)	3040.0
Crystal size/mm^3	0.15 × 0.05 × 0.05
Radiation/Å	Synchrotron (λ = 0.71073)
2Θ range for data collection/°	1.786 to 41.63
Index ranges	-21 ≤ h ≤ 21, -23 ≤ k ≤ 23, -23 ≤ l ≤ 23
Reflections collected	80262
Independent reflections	22356 [R_{int} = 0.1023, R_{sigma} = 0.1684]
Data/restraints/parameters	22356/4685/1909
Goodness-of-fit on F^2	1.083
Final R indexes [I>=2σ (I)]	R_1 = 0.1572, wR_2 = 0.3879
Final R indexes [all data]	R_1 = 0.2410, wR_2 = 0.4602
Largest diff. peak/hole / e Å^{-3}	0.62/-0.46

1.4.4. Tet_{113}

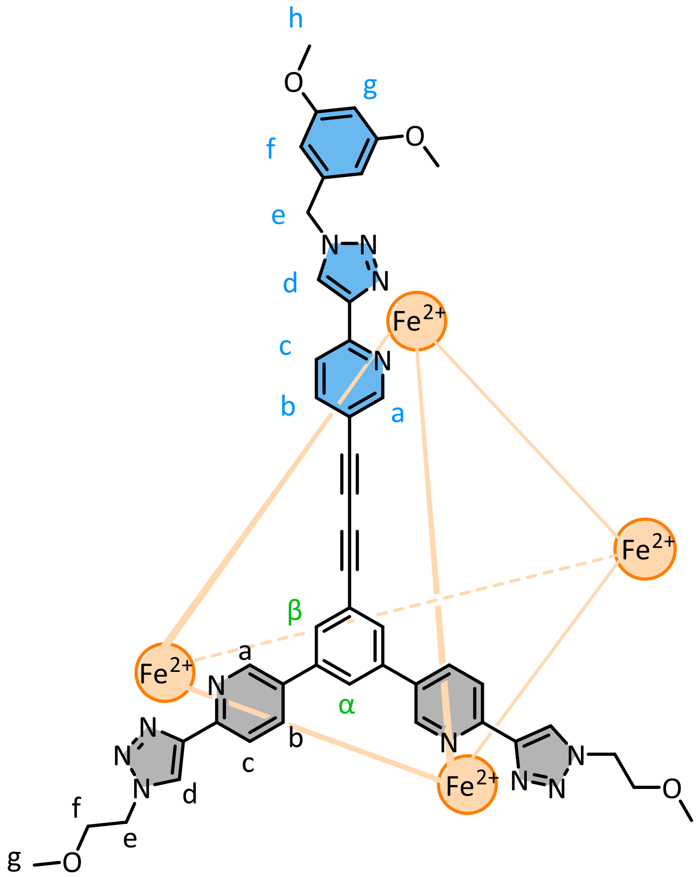

Ligand **L_{113}** (2.95 mg, 3.58 µmol, 1.0 eq) was dissolved in an NMR tube in CD_3CN (600 µL). 50 µL of a 71.5 mM stock solution of $[Fe(BF_4)_2]\cdot 6H_2O$ in CD_3CN (3.58 µmol, 1.0 eq) was added and the tube was well shaken to mix. The resulting solution immediately turned bright orange, indicating complexation. After 15 minutes at room temperature, equilibration to the $[Fe_4(\mathbf{L_{113}})_4](BF_4)_8$ species was observed by ^1H NMR spectroscopy. The solution was heated overnight at 50 °C to ensure full equilibration.

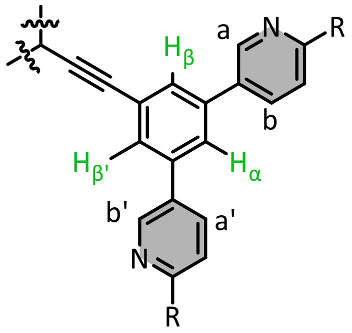

Figure 1.56: The proton environments around the central aromatic ring as assigned in the below spectra.

^1H NMR (800 MHz, CD_3CN, 298 K) δ: 9.31 (br s, 1H, H_a), 9.22 (s, 1H, H_d), 9.13 (s, 1H, $H_{d'}$), 9.04 (s, 1H, H_d), 8.81 (m, 1H, H_c), 8.77 (m, 2H, $H_{a,c'}$), 8.52 (m, 2H, $H_{a',c}$), 8.33 (d, J = 8.0 Hz, 1H, H_b), 8.28 (m, 2H, $H_{b',b}$), 7.77 (br s, 1H, $H_{\beta'}$), 7.42 (br s, 1H, H_β), 7.28 (br s, 1H, H_α), 6.48 (br s, 1H, H_g), 6.34 (br s, 2H, H_f), 5.65 (s, 2H, H_e), 4.71 (m, 4H, H_e), 3.79 (m, 4H, H_f), 3.74 (s, 6H, H_h), 3.31 (s, 3H, H_g), 3.28 (s, 3H, $H_{g'}$);

^1H DOSY NMR (400 MHz, CD_3CN, 298 K) D ($\times 10^{-10}$ m^2s^{-1}): 4.2;

^{19}F NMR (376 MHz, CD_3CN, 298 K) δ: - 151.2;

HR ESI-MS (CH_3CN) m/z = 616.1826 $[Fe_4(L_{113})_4^{8+} + 2\ BF_4^-]^{6+}$ (calc. for $[Fe_4C_{184}H_{160}N_{48}O_{16}B_2F_8]^{6+}$, 616.1781); m/z = 756.6170 $[Fe_4(L_{113})_4^{8+} + 3\ BF_4^-]^{5+}$ (calc. for $[Fe_4C_{184}H_{160}N_{48}O_{16}B_3F_{12}]^{5+}$, 756.6143); m/z = 967.5221 $[Fe_4(L_{113})_4^{8+} + 4\ BF_4^-]^{4+}$ (calc. for $[Fe_4C_{184}H_{160}N_{48}O_{16}B_4F_{16}]^{4+}$, 967.5190); m/z = 1319.0324 $[Fe_4(L_{113})_4^{8+} + 5\ BF_4^-]^{3+}$ (calc. for $[Fe_4C_{184}H_{160}N_{48}O_{16}B_5F_{20}]^{3+}$, 1319.0270)

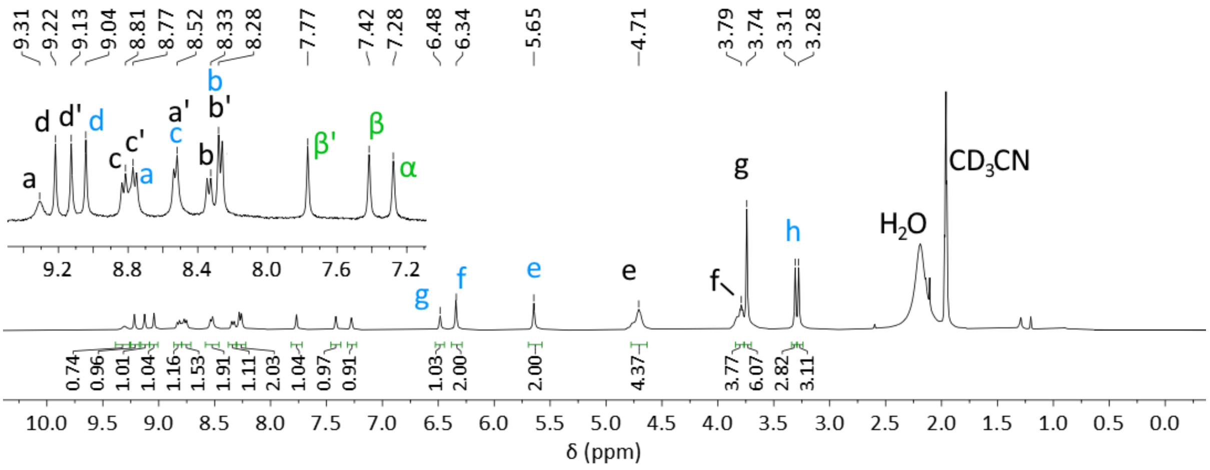

Figure S1.57: ^1H NMR spectrum of **Tet$_{113}$** (800 MHz, CD_3CN, 298 K).

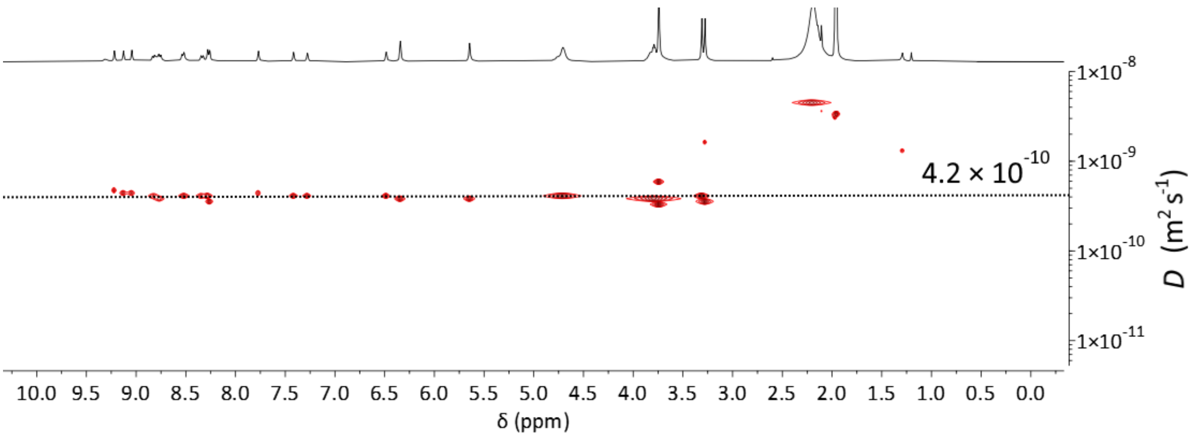

Figure S1.58: ^1H DOSY NMR spectrum of **Tet$_{113}$** (400 MHz, CD_3CN, 298 K).

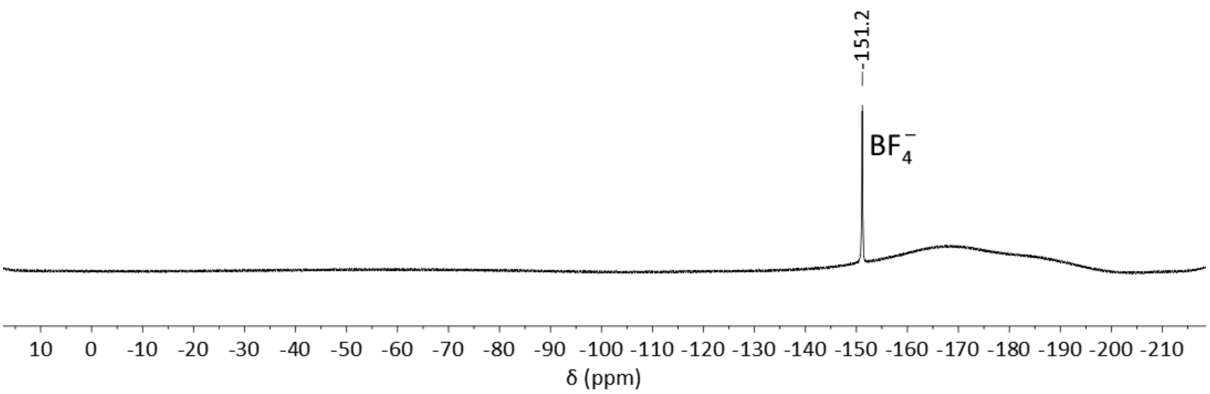

Figure S1.59: ^{19}F NMR spectrum of **Tet$_{113}$** (376 MHz, CD_3CN, 298 K).

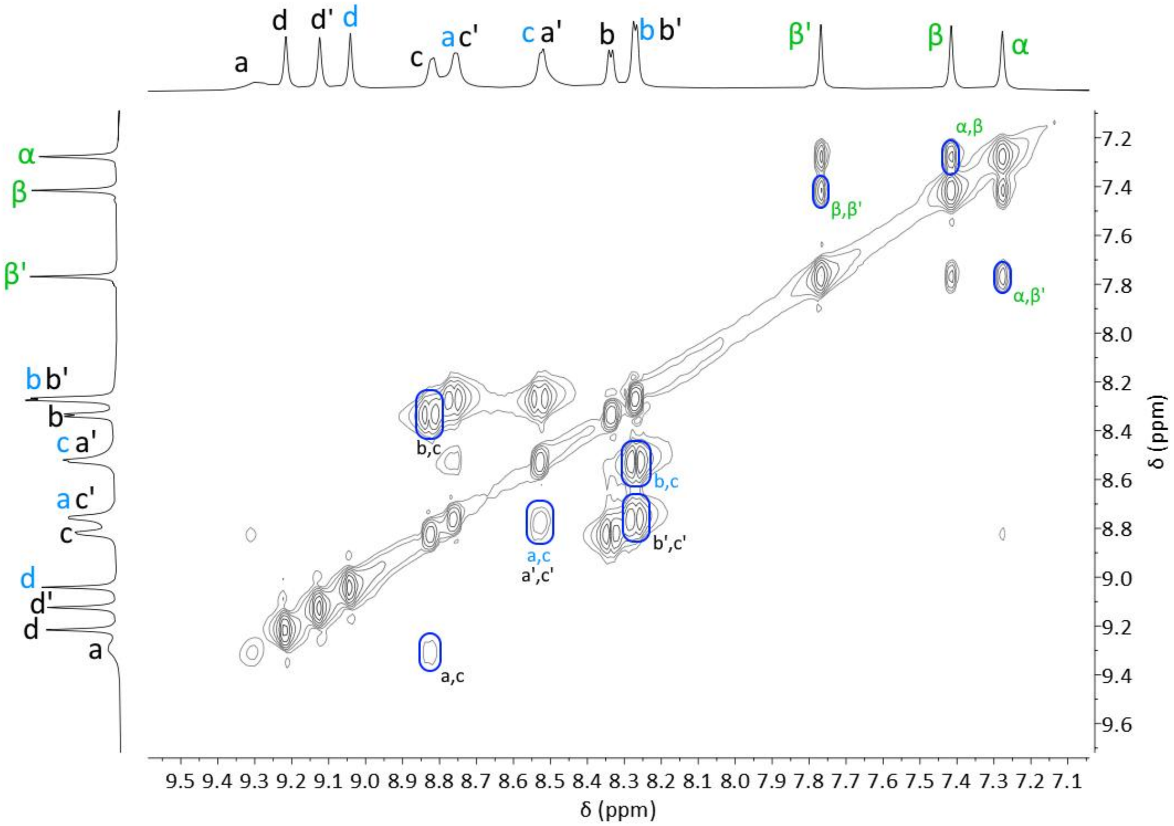

Figure S1.60: Partial ^{1}H TOCSY 2D NMR spectrum of **Tet_{113}** (800 MHz, CD_3CN, 298 K, 200 ms).

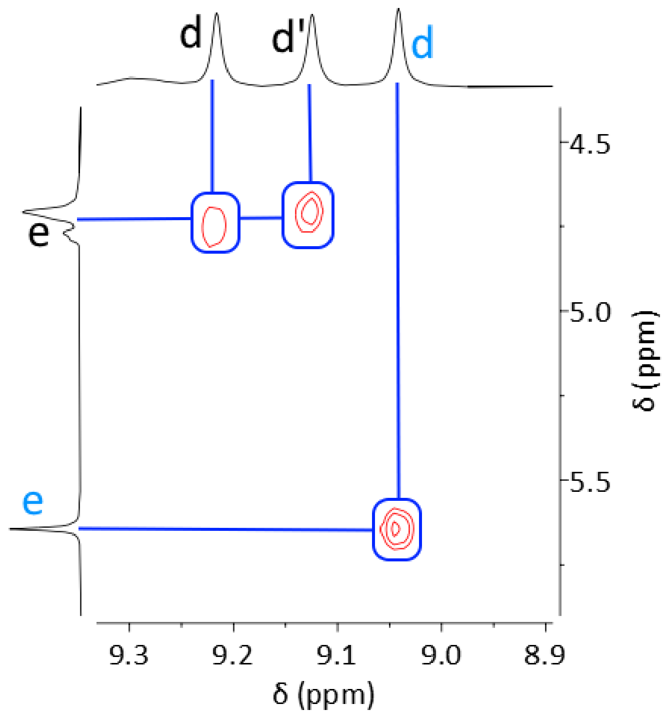

Figure S1.61: Partial ^{1}H NOESY 2D NMR spectrum of **Tet_{113}** (800 MHz, CD_3CN, 298K, 200 ms).

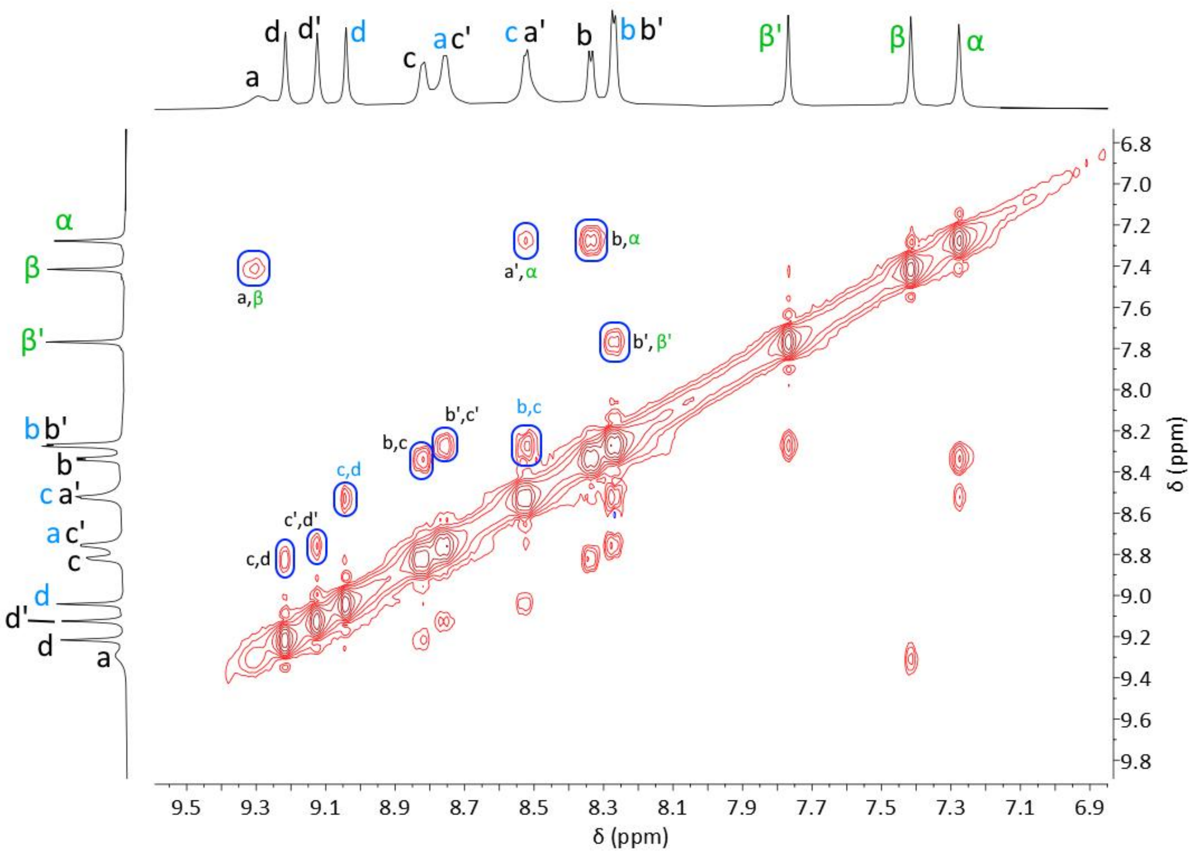

Figure S1.62: Partial ^{1}H NOESY 2D NMR spectrum of **Tet$_{113}$** (800 MHz, CD_3CN, 298K, 200 ms).

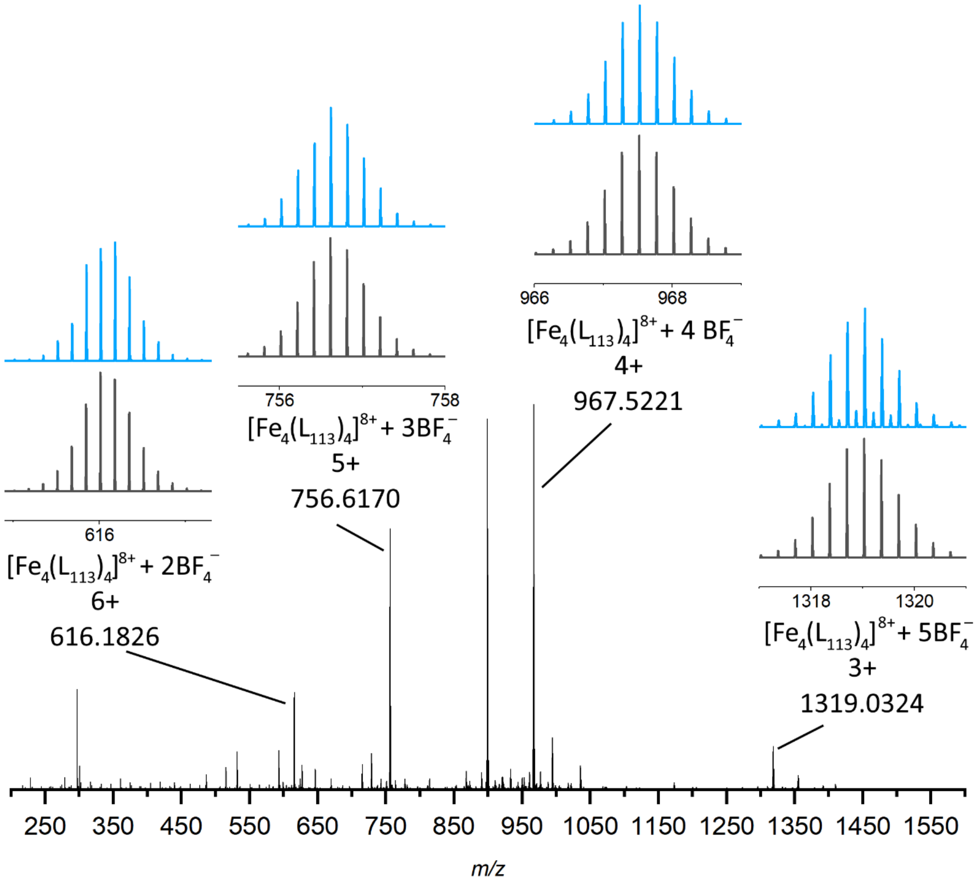

Figure S1.63: HR-ESI mass spectrum of **Tet$_{113}$** (CD_3CN/CH_3CN).

1.4.5. Tet$_{123}$

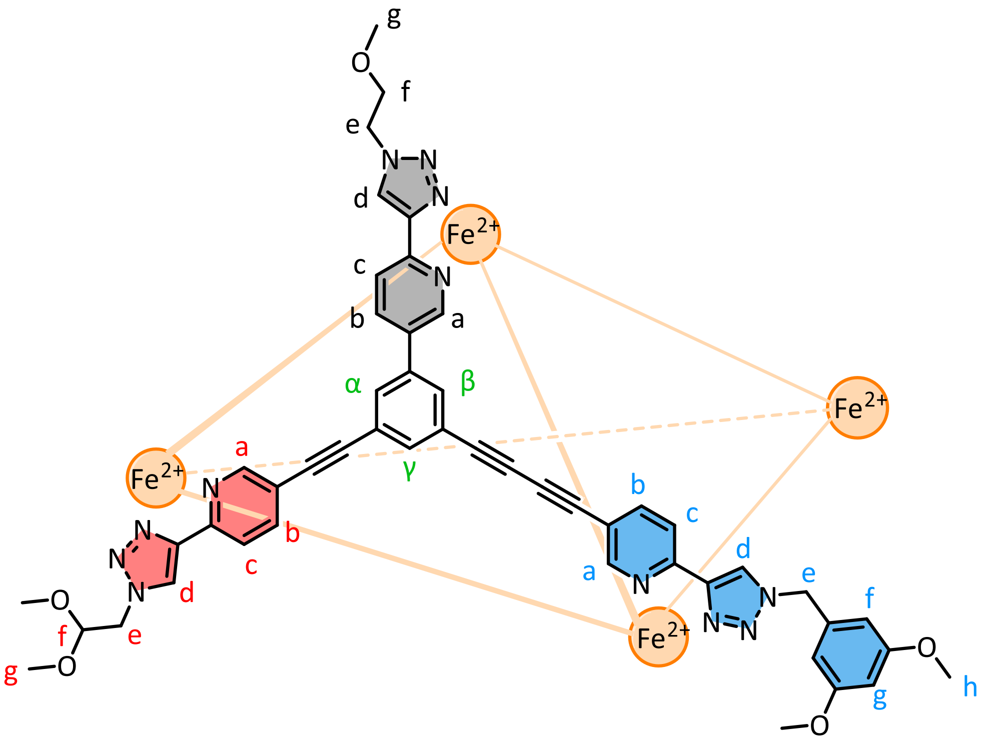

Ligand **L$_{123}$** (3.32 mg, 3.78 µmol, 1.0 eq) was dissolved in an NMR tube in CD_3CN (600 µL). 50 µL of a 75.5 mM stock solution of $[Fe(BF_4)_2]\cdot 6H_2O$ in CD_3CN (3.78 µmol, 1.0 eq) was added and the tube was well shaken to mix. The resulting solution immediately turned bright orange, indicating complexation. After 15 minutes at room temperature, no equilibration to the [Fe$_4$(**L$_{113}$**)$_4$](BF$_4$)$_8$ species was observable by ^{1}H NMR spectroscopy. The solution was heated overnight at 50 °C after which equilibration was observed. Heating was continued for a further 24 hours to ensure full equilibration.

^{1}H NMR (800 MHz, CD_3CN, 298 K) δ: 8.92 (s, 1H, H$_d$), 8.91 (s, 1H, H$_d$), 8.87 (s, 1H, H$_d$), 8.56 (m, 1H, H$_c$), 8.44 (br s, 1H, H$_c$), 8.40 (br s, 1H, H$_b$), 8.32-8.27 (m, 3H, H$_{b,c,a/a}$), 8.20-8.16 (m, 2H, H$_{b,a/a}$), 8.13 (br s, 1H, H$_a$), 7.88 (br s, 1H, H$_{\alpha/\beta}$), 7.54 (s, 1H, H$_\gamma$), 7.16 (br s, 1H, H$_{\alpha/\beta}$), 6.49 (br s, 1H, H$_g$), 6.32 (d, J = 1.5 Hz, 2H, H$_f$), 5.56 (s, 2H, H$_e$), 4.59 (m, 3H, H$_{e,f}$), 4.53 (m, 2H, H$_e$), 3.75 (s, 6H, H$_h$), 3.74 (m, 2H, H$_f$), 3.29 (s, 3H, H$_{g/g/g'}$), 3.27 (s, 3H, H$_{g/g/g'}$), 3.25 (s, 3H, H$_{g/g/g'}$);
^{1}H DOSY NMR (400 MHz, CD_3CN, 298 K) D ($\times 10^{-10}$ m^{2}s^{-1}): 3.5;
^{19}F NMR (376 MHz, CD_3CN, 298 K) δ: -146.7;
HR ESI-MS (CH_3CN) m/z = 652.1853 $[Fe_4(L_{123})_4{}^{8+} + 2\ BF_4{}^{-}]^{6+}$ (calc. for $[Fe_4C_{196}H_{168}N_{48}O_{20}B_2F_8]^{6+}$, 652.1856); m/z = 799.8231 $[Fe_4(L_{123})_4{}^{8+} + 3\ BF_4{}^{-}]^{5+}$ (calc. for $[Fe_4C_{196}H_{168}N_{48}O_{20}B_3F_{12}]^{5+}$, 799.8236); m/z = 1021.5302 $[Fe_4(L_{123})_4{}^{8+} + 4\ BF_4{}^{-}]^{4+}$ (calc. for $[Fe_4C_{196}H_{168}N_{48}O_{20}B_4F_{16}]^{4+}$, 1021.5310)

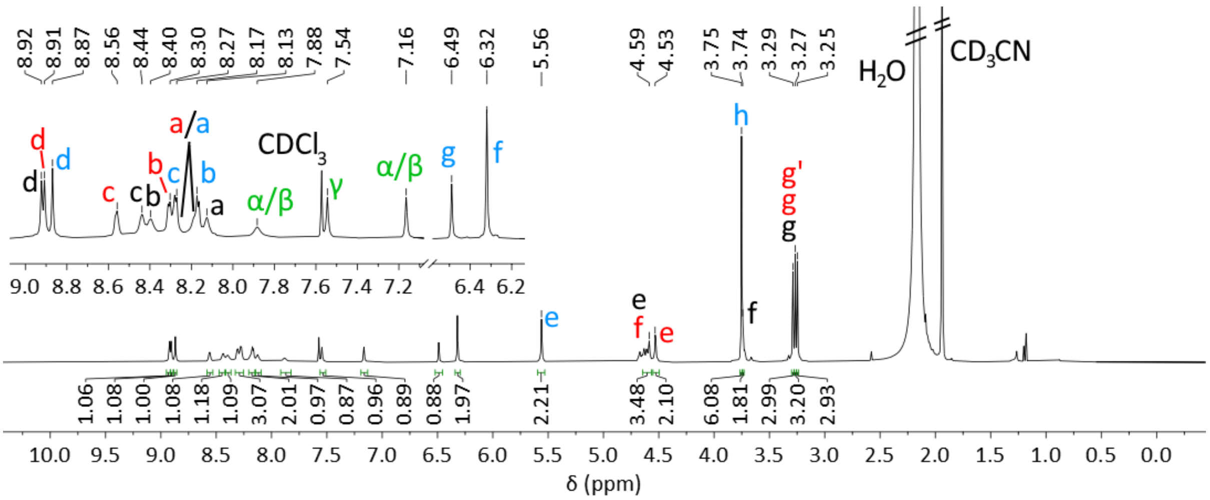

Figure S1.64: ^{1}H NMR spectrum of **Tet$_{123}$** (800 MHz, CD_3CN, 298 K).

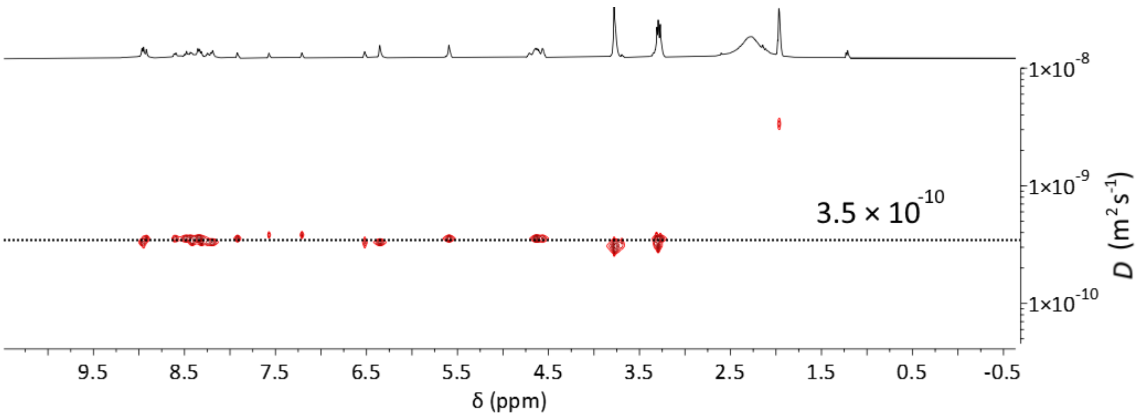

Figure S1.65: ^{1}H DOSY NMR spectrum of **Tet$_{123}$** (400 MHz, CD_3CN, 298 K).

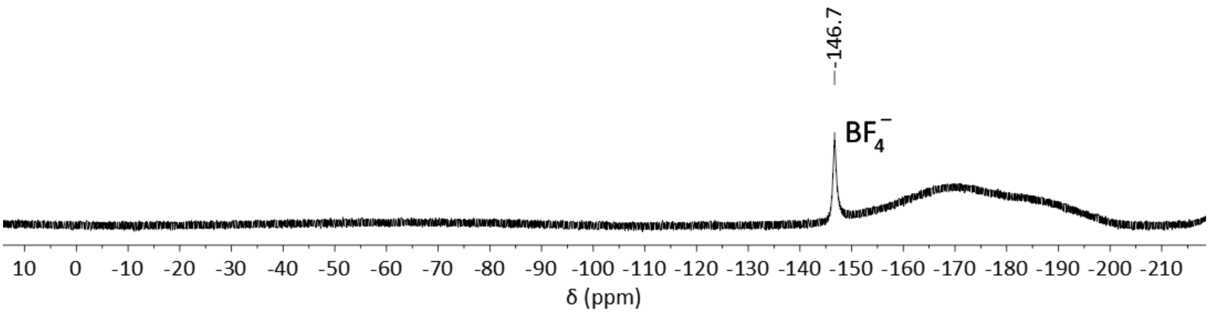

Figure S1.66: ^{19}F NMR spectrum of **Tet$_{123}$** (376 MHz, CD_3CN, 298 K).

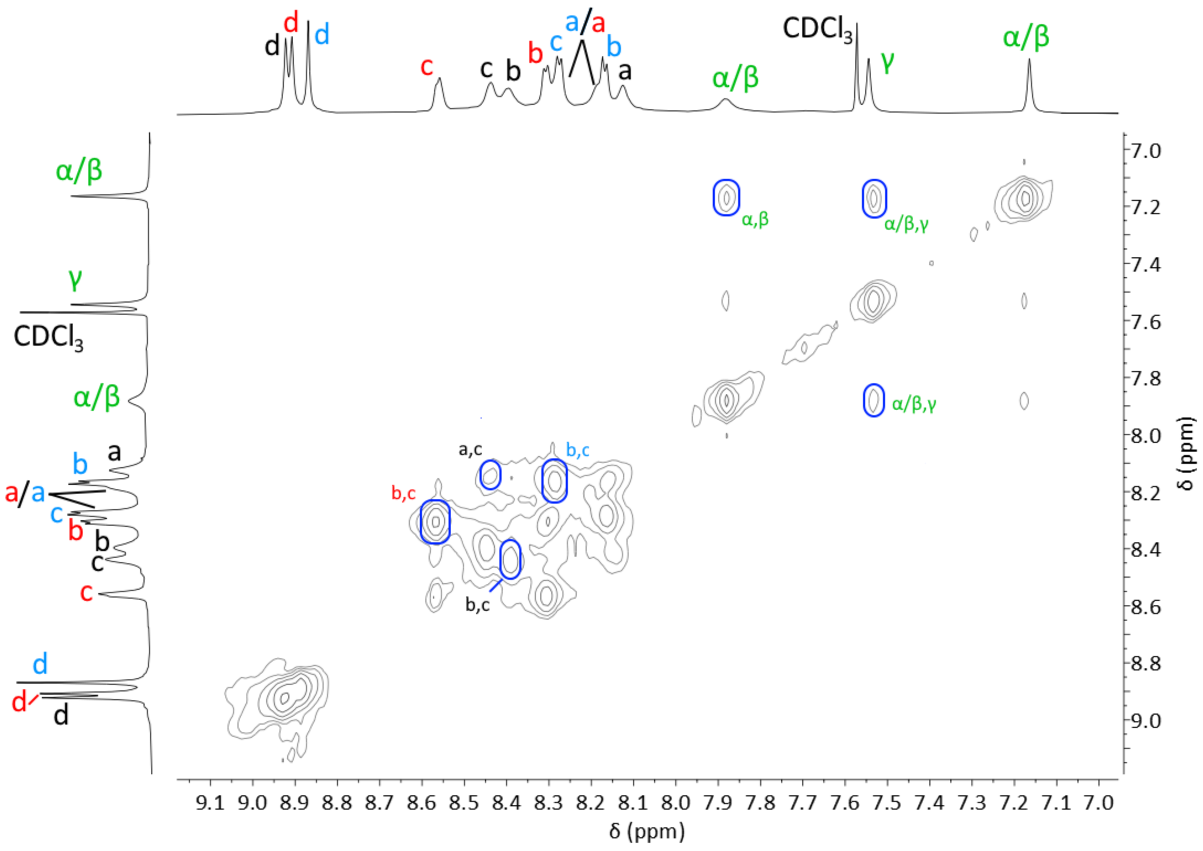

Figure S1.67: Partial ^{1}H TOCSY 2D NMR spectrum of **Tet$_{123}$** (800 MHz, CD_3CN, 298 K, 200 ms).

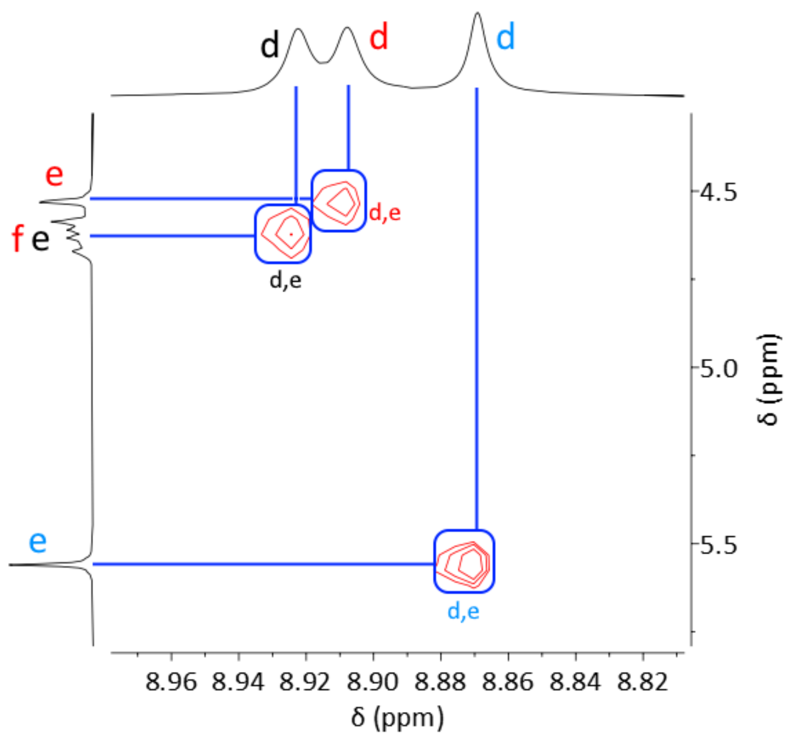

Figure S1.68: Partial ^{1}H NOESY 2D NMR spectrum of **Tet$_{123}$** (800 MHz, CD_3CN, 298 K, 200 ms).

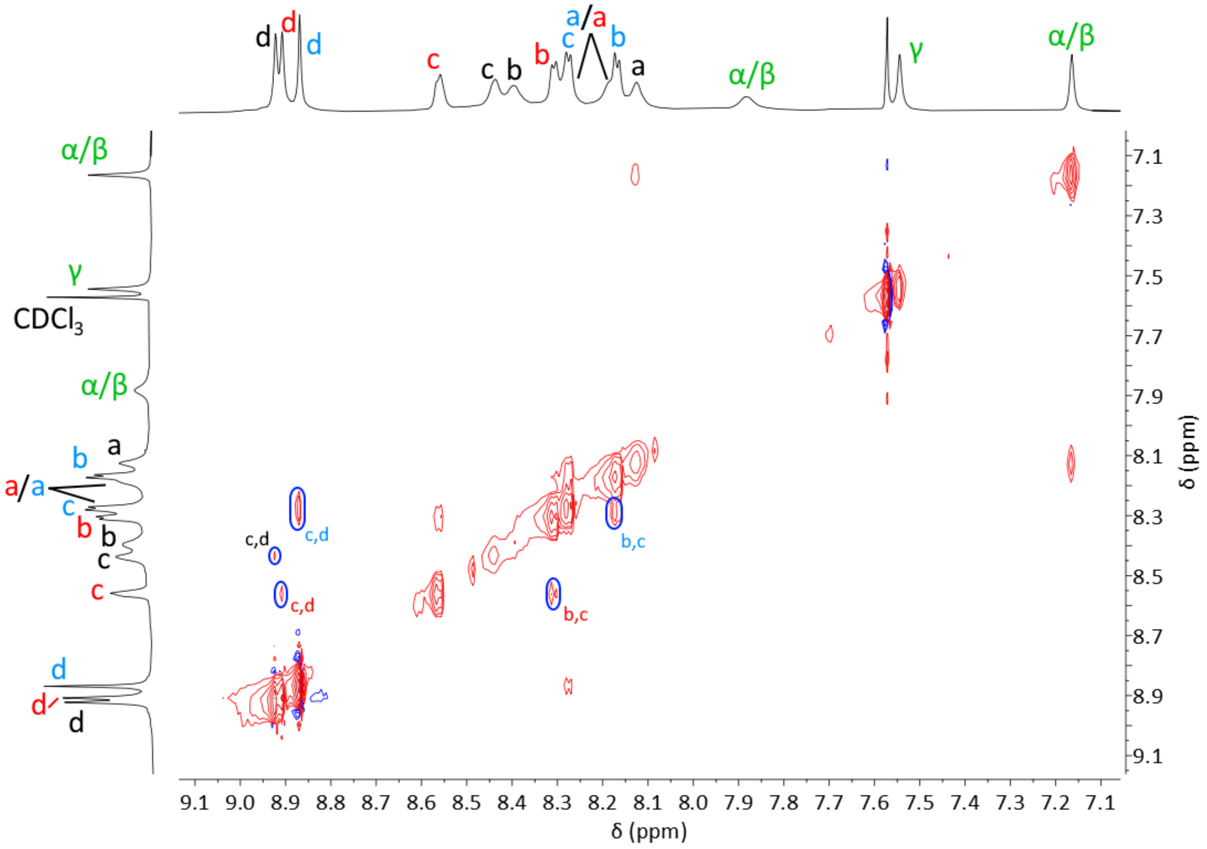

Figure S1.69: Partial ^{1}H NOESY 2D NMR spectrum of **Tet$_{123}$** (400 MHz, CD_3CN, 298 K, 200 ms).

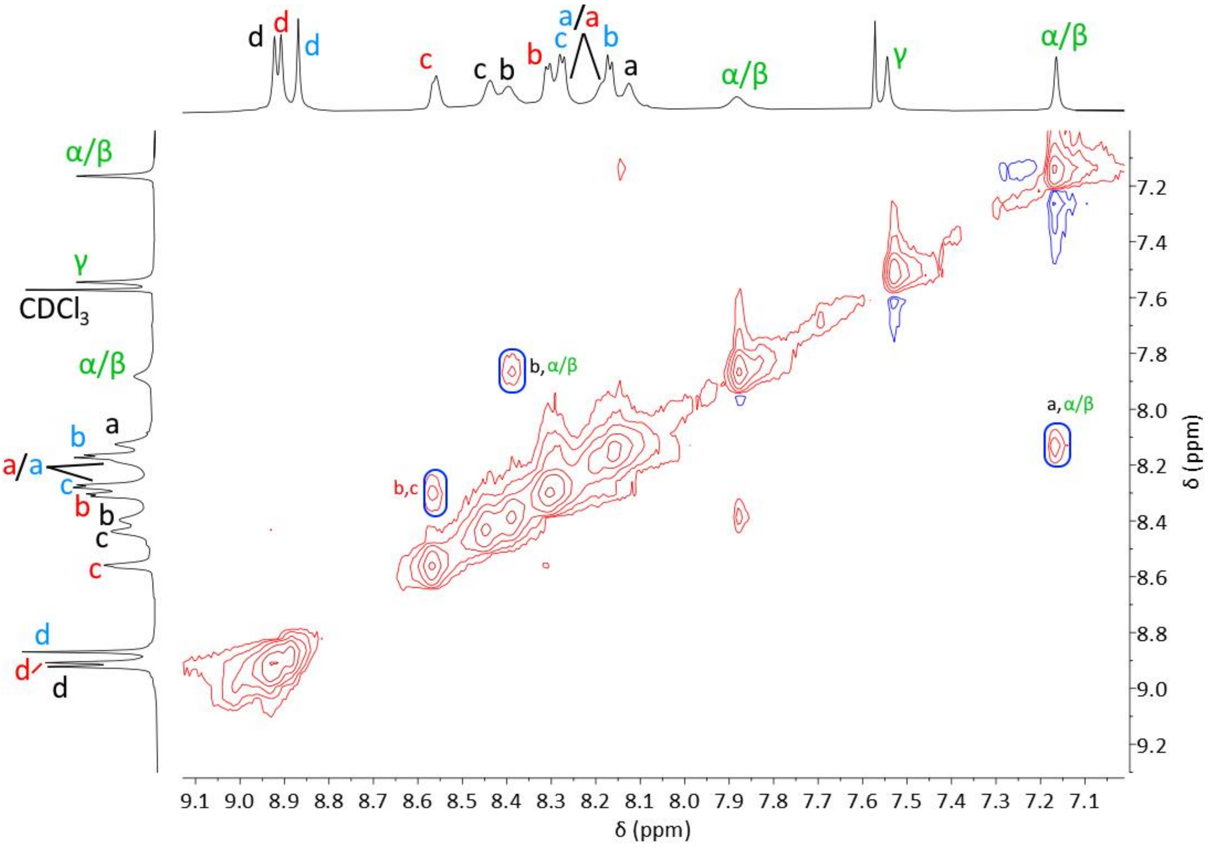

Figure S1.70: Partial ^{1}H NOESY 2D NMR spectrum of **Tet$_{123}$** (800 MHz, CD_3CN, 298 K, 200 ms).

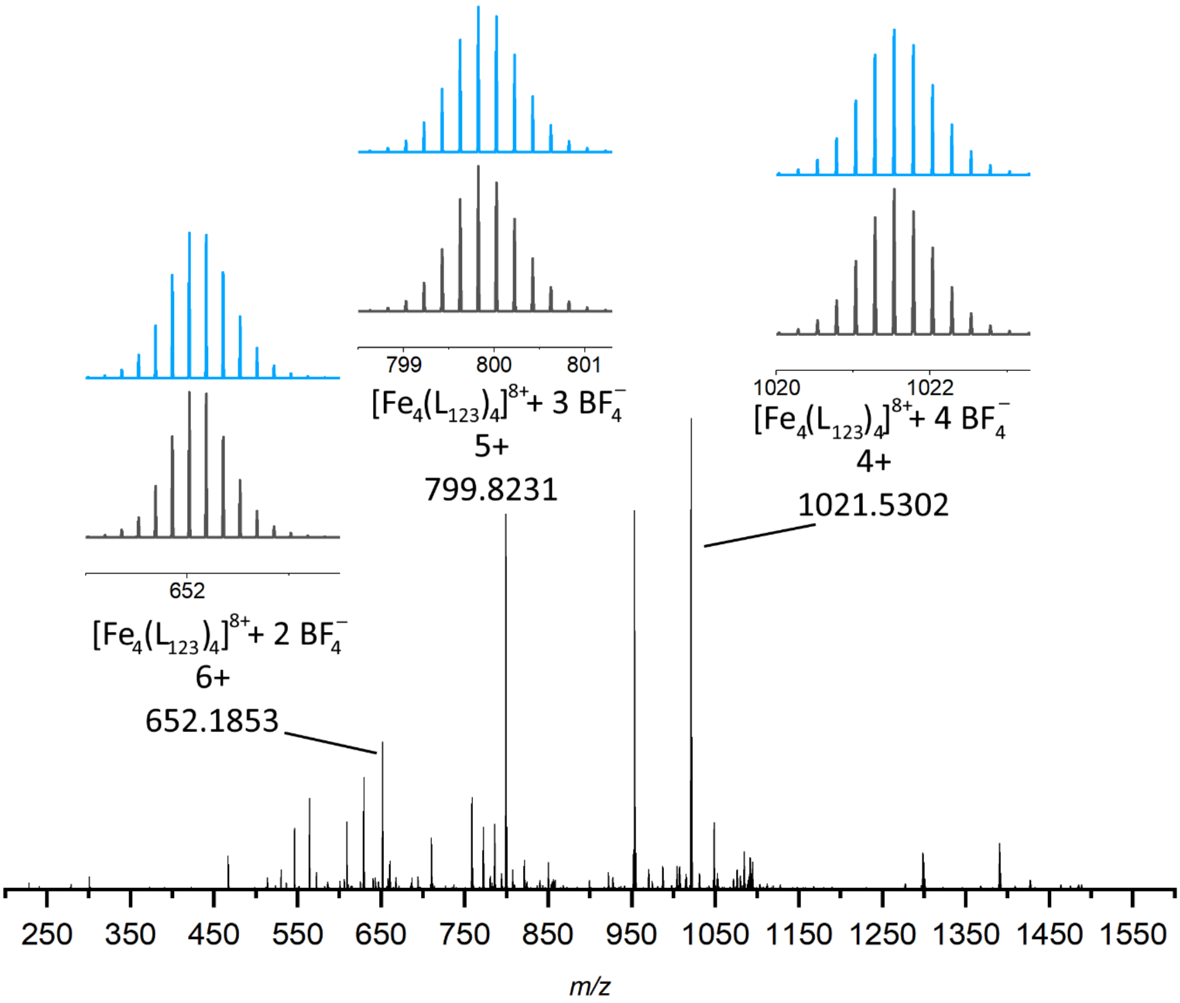

Figure S1.71: HR-ESI mass spectrum of **Tet_{113}** (CD_3CN/CH_3CN).

1.4.6. Tet_{111}'

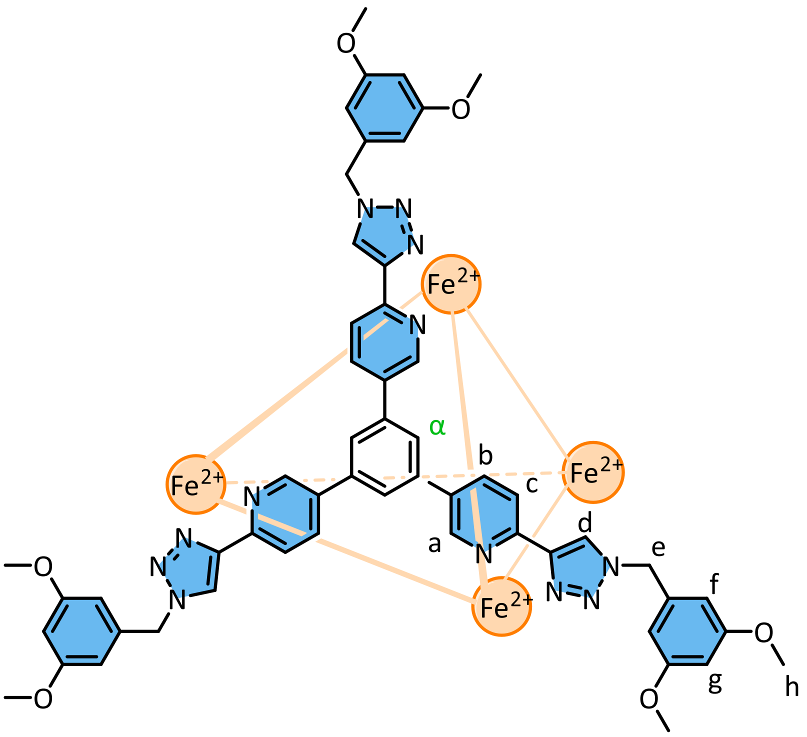

Ligand **L_{111}'** (2.00 mg, 2.08 µmol, 1.0 eq) was dissolved in an NMR tube in CD_3CN (600 µL). 24 µL of a 85.2 mM stock solution of $[Fe(BF_4)_2]\cdot 6H_2O$ in CD_3CN (2.08 µmol, 1.0 eq) was added and the tube was well shaken to mix. The resulting solution immediately turned bright orange, indicating complexation. After 15 minutes at room temperature, full equilibration to the $[Fe_4(\mathbf{L_{111}})_4](BF_4)_8$ species was observed by ^1H NMR spectroscopy.

^1H NMR (400 MHz, CD_3CN, 298 K) δ: 8.94 (1H, br, H_d), 8.37 (1H, br, H_c), 8.25 (1H, br, H_b), 7.53 (1H, s, H_a), 7.18 (1H, s, H_α), 6.44 (1H, s, H_g), 6.40 (2H, s, H_f), 5.65 (2H, AB quartet, J = 61.2 Hz, 15.3 Hz, H_e), 3.64 (6H, s, H_h);
^1H DOSY NMR (400 MHz, CD_3CN, 298 K) D ($\times 10^{-10}$ m^2s^{-1}): 3.6;
^{19}F NMR (376 MHz, CD_3CN, 298 K) δ: -151.3;
HR ESI-MS (CH_3CN) m/z = 706.8785 $[Fe_4(L_{111}')_4^{8+} + 2\ BF_4^-]^{6+}$ (calc. for $[Fe_4C_{216}H_{192}N_{48}O_{24}B_2F_8]^{6+}$, 706.8832), m/z = 865.4479 $[Fe_4(L_{111}')_4^{8+} + 3\ BF_4^-]^{5+}$ (calc. for $[Fe_4C_{216}H_{192}N_{48}O_{24}B_3F_{12}]^{5+}$, 865.4547), m/z = 1103.5726 $[Fe_4(L_{111}')_4^{8+} + 4\ BF_4^-]^{4+}$ (calc. for $[Fe_4C_{216}H_{192}N_{48}O_{24}B_4F_{16}]^{4+}$, 1103.5732)

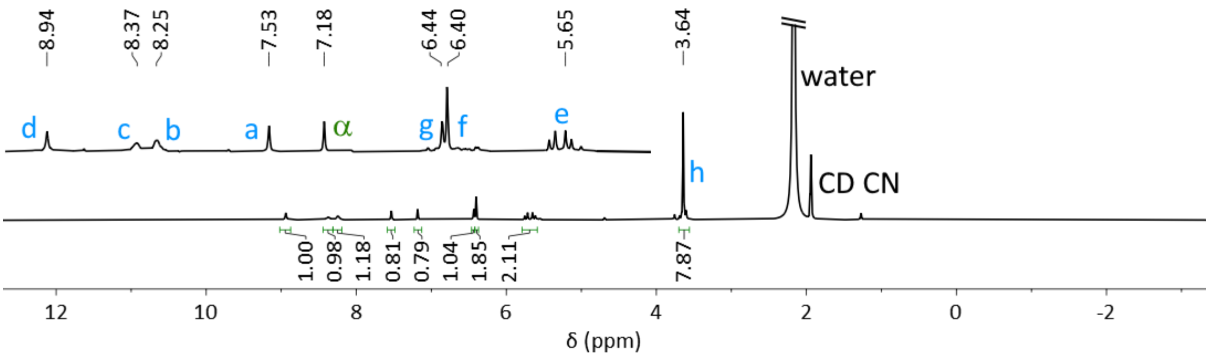

Figure S1.72 ^1H NMR spectrum of **Tet_{111}'** (800 MHz, CD_3CN, 298 K).

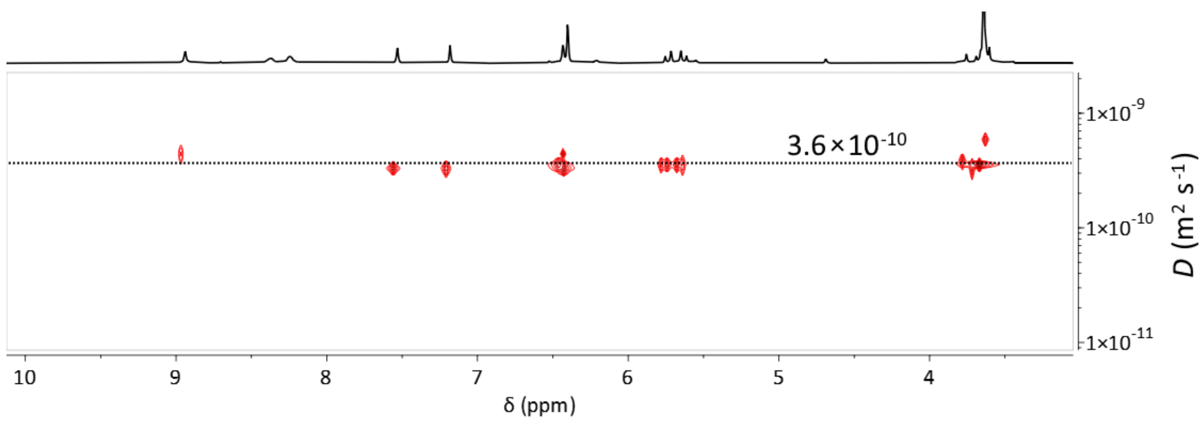

Figure S1.73 ^{1}H DOSY NMR spectrum of **Tet₁₁₁'** (400 MHz, CD_3CN, 298 K).

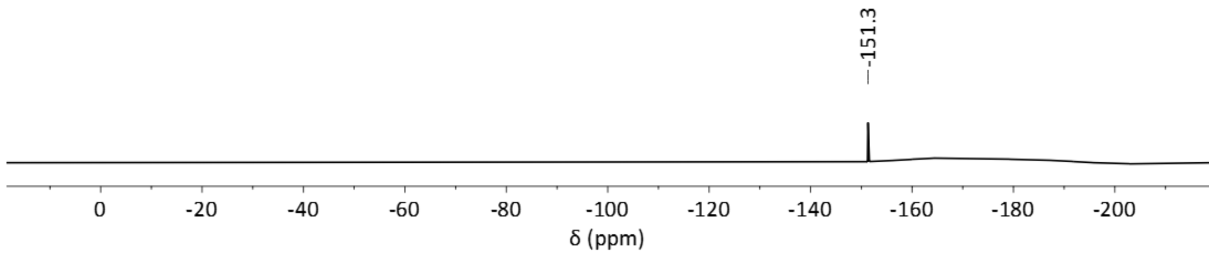

Figure 1.74 ^{19}F NMR spectrum of **Tet₁₁₁'** (376 MHz, CD_3CN, 298 K).

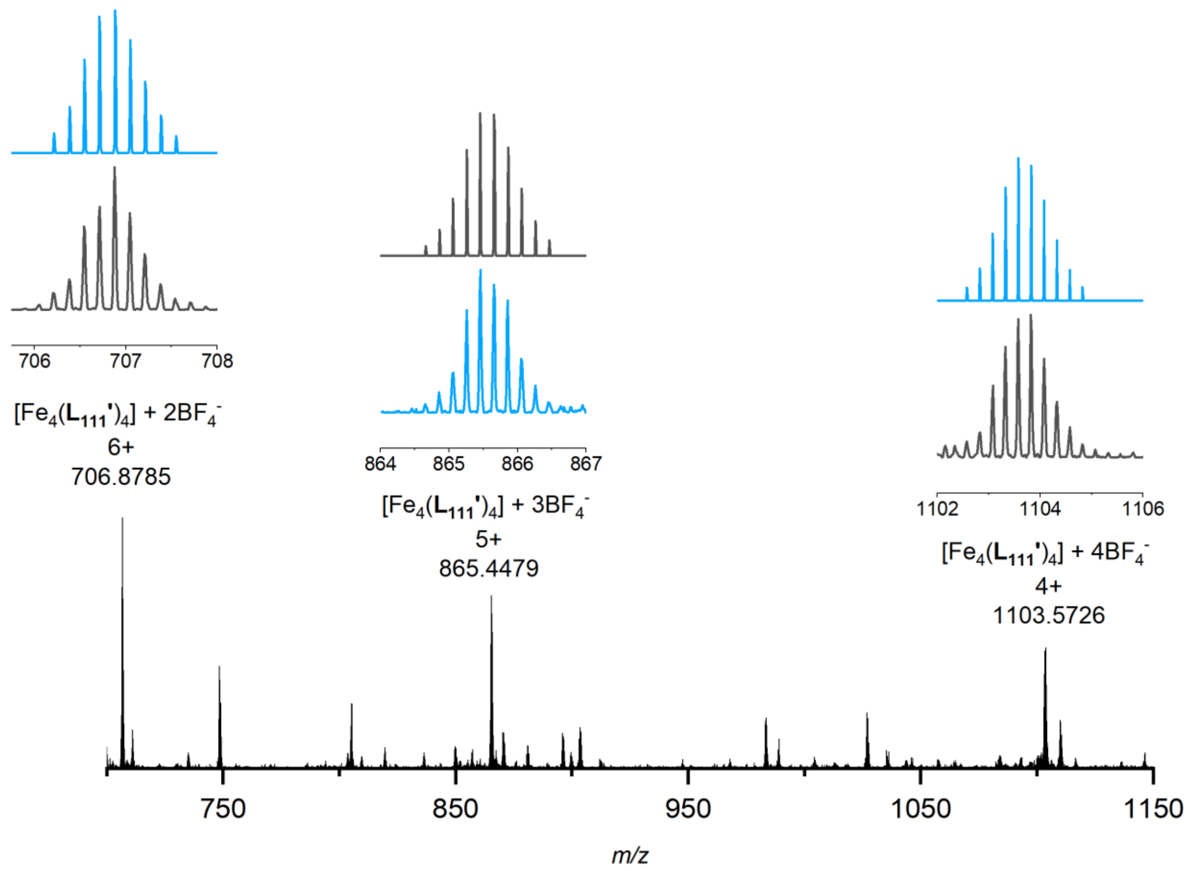

Figure S1.75 HR-ESI mass spectrum of **Tet₁₁₁'** (CD_3CN/CH_3CN).

2. Computations

2.1. GFN-xtb

Representative molecular models of the molecules were obtained by initial modelling using Spartan,[9] and optimising using GFN-xtb.[10] Standard settings were used, with an acetonitrile solvent field, at 298 K.

Molecular models available in xyz format on request.

For comparison, GFN-xtb structures were overlaid with X-ray or DFT structures in Mercury aligning the four Fe(II) metal ions.

2.1.1. Comparison between GFN-xtb model and X-ray structure of T_{111}

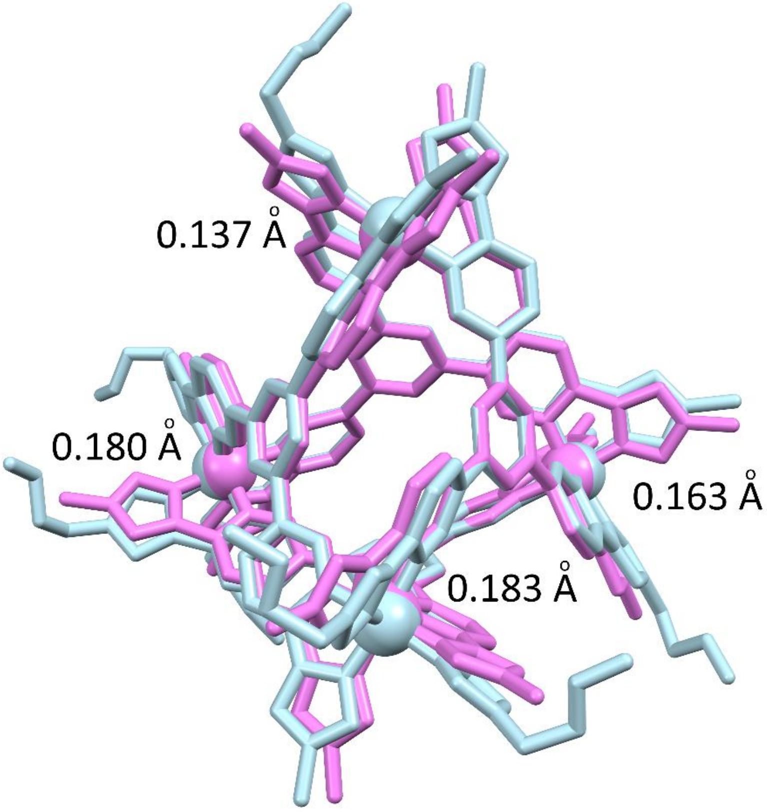

Figure S2.1 Overlaid structures of $\mathbf{T_{111}}$ from GFN-xtb (violet) and X-ray crystallography (light blue) with distances between Fe(II) irons from the two different structures shown.

2.1.2. Comparison between GFN-xtb model and X-ray structure of T_{112}

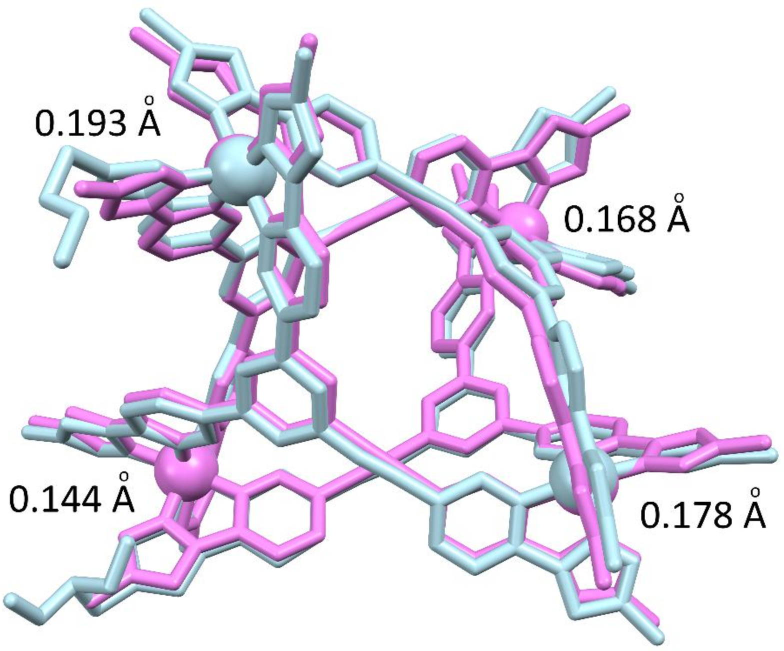

Figure S2.2 Overlaid structures of $\mathbf{T_{112}}$ from GFN-xtb (violet) and X-ray crystallography (light blue) with distances between Fe(II) irons from the two different structures shown.

2.1.3. Comparison between GFN-xtb model and X-ray structure of T_{122}

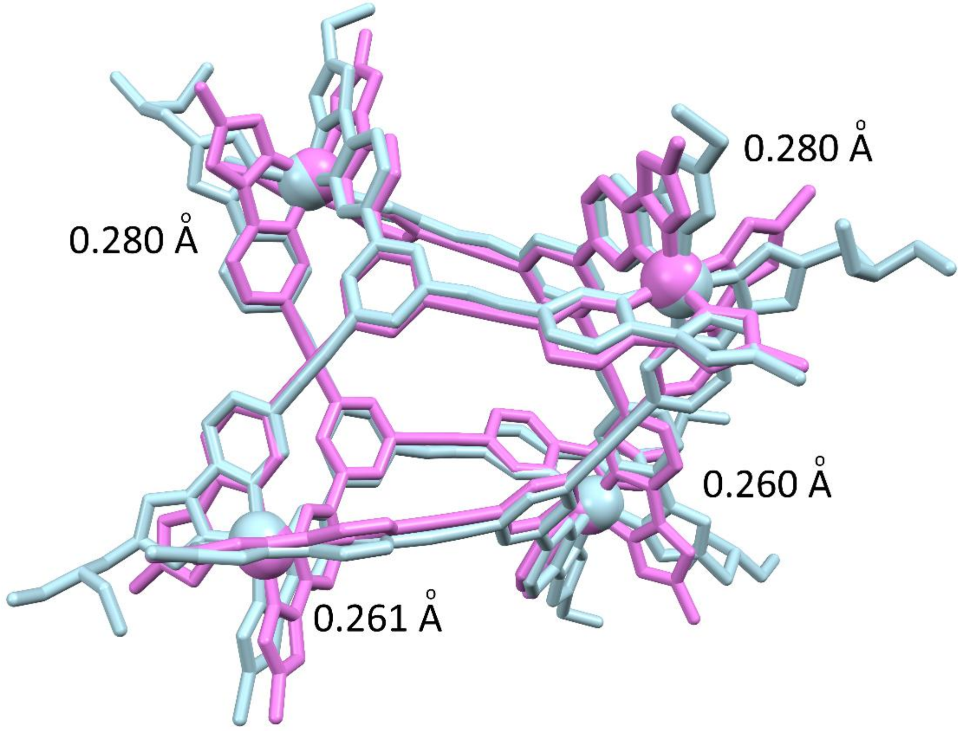

Figure S2.3 Overlaid structures of $\mathbf{T_{122}}$ from GFN-xtb (violet) and X-ray crystallography (light blue) with distances between Fe(II) irons from the two different structures shown.

2.1.4. Comparison between GFN-xtb model and DFT structure of T_{123} (MapBΛ_4)

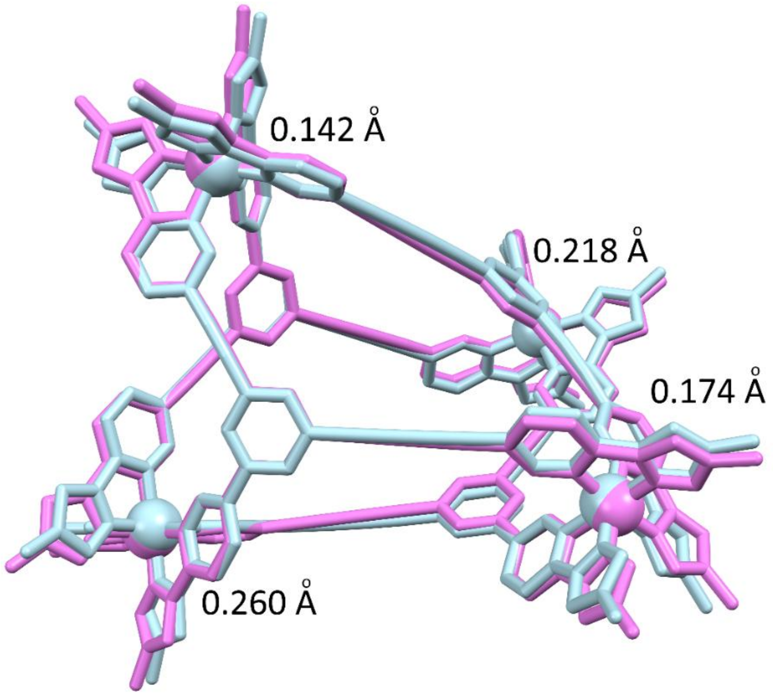

Figure S2.4 Overlaid structures of $\mathbf{T_{123}}$ from GFN-xtb (violet) and DFT (light blue) with distances between Fe(II) irons from the two different structures shown.

2.2. DFT calculations

Density functional theory (DFT) calculations were performed using ORCA version 6.0.1.[11] All structures were fully optimised using the r2SCAN-3c functional.[12] C and H atoms were treated with the def2-SVP basis set, while N and Fe atoms used the def2-TZVP basis set.[13] The resolution of identity approximation was employed with the def2/J auxiliary basis set.[14] All optimisations used tight convergence criteria for self-consistent field (SCF) cycles.

For the $\mathbf{T_{123}}$ diastereomers, the **MapB**Λ_4 structure was 7 kJ mol^{-1} lower in energy than the **MapB**Δ_4 structure.

Molecular models and raw simulation data have been deposited at Zenodo (DOI code: 10.5281/zenodo.14847430).

3. Structure relationship studies

3.1. Calculating structural distortion

A terahedron/disphenoid has 12 unique Fe-Fe-Fe angles (3 on each of the 4 four faces). These should average to 60°, and the standard deviation of these 12 angles gives a measure of the degree of distortion away from a 'perfect' tetrahedron. These 12 unique angles were obtained from the GFN-xtb-calculated structures for the 5 cages. In order to cross-check the values, they were compared to the same values from all distinct cages in any X-ray crystal structures, and to the DFT-obtained structure of $\mathbf{T_{123}}$. These values are given in the table below. Note that for tetragonal and rhombic disphenoids, the angles sort themselves into 3 sets of 4.

Table S3.1 Individual angles for each cage investigated in this study, derived from either GFN-xtb, X-ray crystallography, or DFT computations. The average angle and the standard deviation are also given. All values are degrees (°). The data from the X-ray structures of $\mathbf{T_{111}}$ and $\mathbf{T_{113}}$ are duplicated as there were two distinct cages within the unit cell.

	T111	T112	T113	T122	T123
GFN-xtb	59.81	49.92	41.11	52.00	44.69
	59.87	49.07	41.08	51.81	44.72
	59.87	50.45	41.42	52.90	44.73
	59.91	50.46	41.67	55.73	44.76
	59.91	61.01	64.43	56.17	62.05
	59.98	62.10	64.91	56.94	62.11
	60.04	62.74	65.48	56.94	62.24
	60.04	62.99	66.07	57.34	62.30
	60.08	66.56	72.51	69.77	72.99
	60.09	67.98	72.85	70.10	73.07
	60.18	68.19	73.98	72.02	73.13
	60.21	68.53	74.49	72.27	73.21
Average	60.00	60.00	60.00	60.33	60.00
Std Dev	0.12	7.48	13.64	7.81	11.69
X-ray structure	59.25, 59.55	49.66, 50.03		50.38	
	59.28, 59.60	49.69, 50.24		50.44	
	59.66, 59.82	49.78, 50.71		50.44	
	59.83, 59.82	50.23, 50.72		50.54	
	59.93, 59.95	62.32, 64.30		57.57	
	60.06, 59.97	62.76, 64.51		57.66	
	60.08, 60.06	62.94, 64.78		57.86	
	60.15, 60.12	66.65, 64.78		57.97	
	60.17, 60.15	66.65, 64.87		71.59	
	60.19, 60.21	67.01, 64.98		71.77	
	60.58, 60.24	67.39, 64.99		71.79	
	60.65, 60.49	67.99, 65.10		71.99	
Average	59.99, 60.00	60.26, 60.00		60.00	
Std Dev	0.42, 0.26	7.58, 6.78		8.85	
DFT					45.10
					45.08
					45.09
					45.12
					62.07
					62.07
					62.09
					62.10
					72.80
					72.80
					72.83
					72.85
Average					60.00
Std Dev					11.41

3.2. DMSO stability studies

A solution containing equimolar amounts of the 5 cages in [D_3]acetonitrile was treated with aliquots of [D_6]DMSO and cage stability was monitored by ^{1}H NMR spectroscopy. Broadening followed by disappearance of cage peaks was diagnostic of coordinative fluxionality, i.e. the breakdown of cage coordinative integrity.

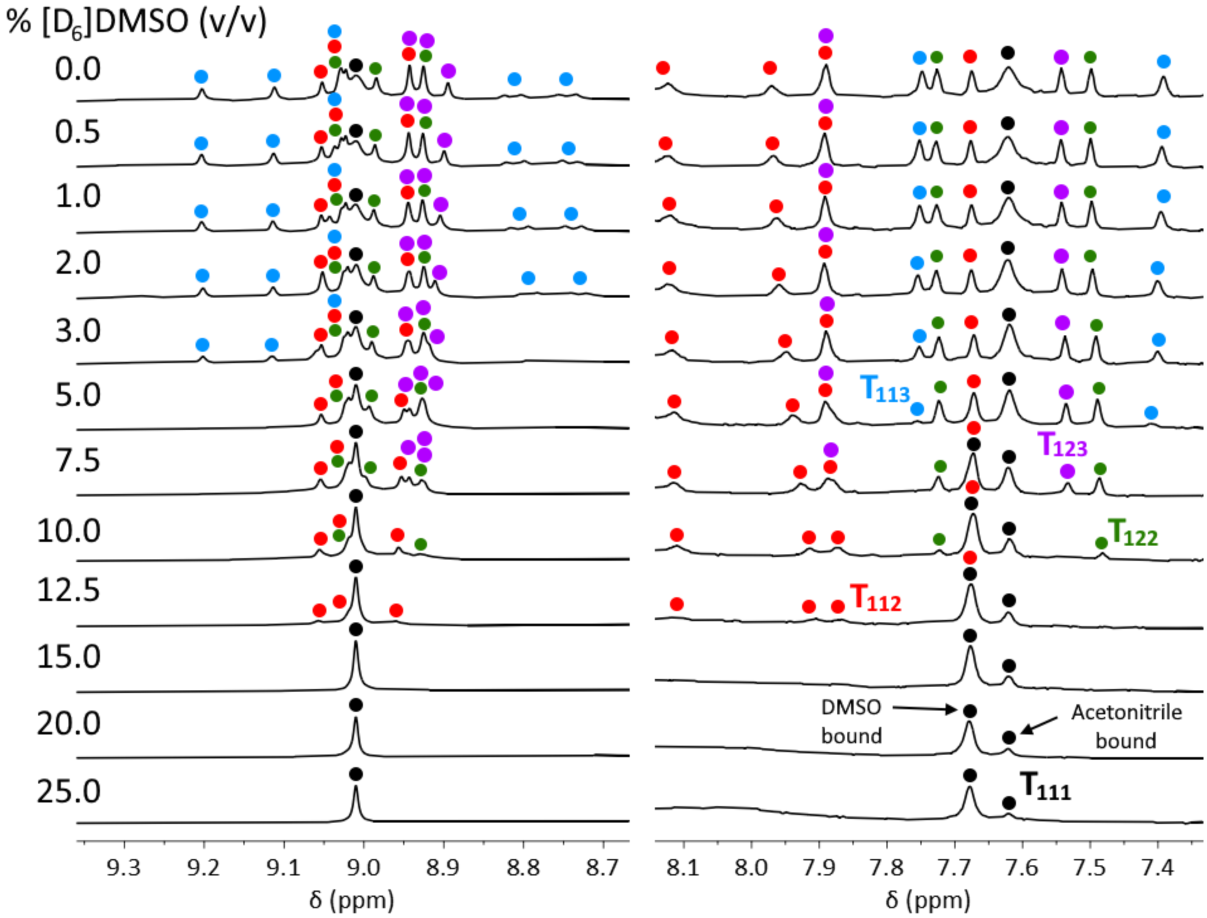

Figure S3.1 Stability studies carried on via ^{1}H NMR spectroscopy (400 MHz, CD_3CN/[D_6]DMSO, 298 K) of $\mathbf{T_{111}}$, $\mathbf{T_{112}}$, $\mathbf{T_{113}}$, $\mathbf{T_{122}}$ and $\mathbf{T_{123}}$. Cage peak identity indicated with coloured dots: $\mathbf{T_{111}}$ (●), $\mathbf{T_{112}}$ (●), $\mathbf{T_{113}}$ (●), $\mathbf{T_{122}}$ (●) and $\mathbf{T_{123}}$ (●). The addition of [D_6]DMSO resulted in changes to chemical shifts of the cage species. For reasons of convenience, the spectra were arbitrarily referenced to the H(d) peak of $\mathbf{T_{111}}$ (left spectra) and the H(a) peak of $\mathbf{T_{111}}$ (right spectra). Note the splitting of the H(a) peak into two environments at higher DMSO concentrations. The other resonances, further from the cavity, did not split. Given that ^{19}F NMR speectra shows that the anion is not encapsulated, solvent must therefore be bound in the cavity. We attribute the splitting of the H(a) and H(α) resonances into two to encapsulation of either acetonitrile or DMSO, and the changing of the proportionate sizes of the peaks to the increasing percentage of DMSO altering equilibrium position of binding of the two different solvents.

To check that the differences in stability were not due to the different strengths of the donor sites due to different substitutions of the ligand arms, the stability of $\mathbf{T_{111}'}$ towards DMSO was also assessed. Despite the 1-(3,5-dimethoxybenzyl) arm being present on the least stable cages in the main study, $\mathbf{T_{111}'}$ showed stability towards [D_6]DMSO up to the same 25% volume as for $\mathbf{T_{111}}$, indicating that the primary driver for the stability of the complexes was their global distortion away from a regular tetrahedron.

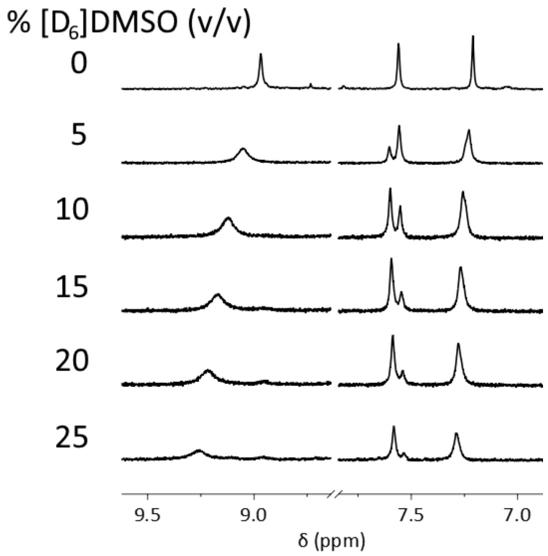

Figure S3.2 Stability studies carried on via ^{1}H NMR spectroscopy (400 MHz, CD_3CN/[D_6]DMSO, 298 K) of **T_{111}'**. The same splitting of the H_a proton which we attributed to solvent encapsulation in **T_{111}** was evident here.

We also note that the ^{1}H NMR spectrum of ligand **T_{123}** gives information about the relative electronic character of the three different arms. In CD_3CN, the triazole peaks of all three arms are very close in chemical shift, with those of **Arm1** and **Arm3** being coincident, and that of **Arm2** being slightly upfield and hence more electron rich. For the H_a proton at the other end of the bidentate site, the order from downfield to upfield is **Arm1**, **Arm2** and then **Arm3**. Similar trends showing that **Arm1** is more electron deficient are also evident for protons H_b and H_c.

3.3. Electrochemistry

Cyclic voltammetry and square wave voltammetry were performed using an Autolab PFSTAT204 potentiostat in a standard 3-electrode setup comprising a glassy carbon working electrode (CH Instruments), platinum wire counter electrode and "leakless" Ag/AgCl reference electrode (eDAQ), housed in a Faraday cage. Potentials were referenced in situ to the ferrocenium/ferrocene ($Fc^{+}/0$) couple and converted to SCE using $Fc^{+}/0$ = +0.4 V.[15] Electrodes were cleaned prior to each experiment; the working electrode was polished using 0.05 µm alumina powder on a felt pad with deionized water. Complex solutions were prepared in freshly distilled acetonitrile (0.2 mM) with $[TBA]PF_6$ as the supporting electrolyte (100 mM) and degassed with Ar for 8 min prior to analysis.

Table S3.2 Summary of electrochemical data.

Compound	$E°'_{ox}$ (ΔE_p)/ V vs SCE[a]
**T_{111}**	1.30 (0.191)
**T_{112}**	1.28 (0.121)
**T_{122}**	1.27 (0.121)
**T_{123}**	1.28 (0.141)
**T_{113}**	1.21 (0.131)<br>1.65 (-)[b]

[a]Measured at 0.2 mM analyte in acetonitrile, 100 mM $TBAPF_6$ supporting electrolyte. [b]Cathodic peak potential (E_{pa}) reported for irreversible second oxidative process, confirmed using square wave voltammetry.

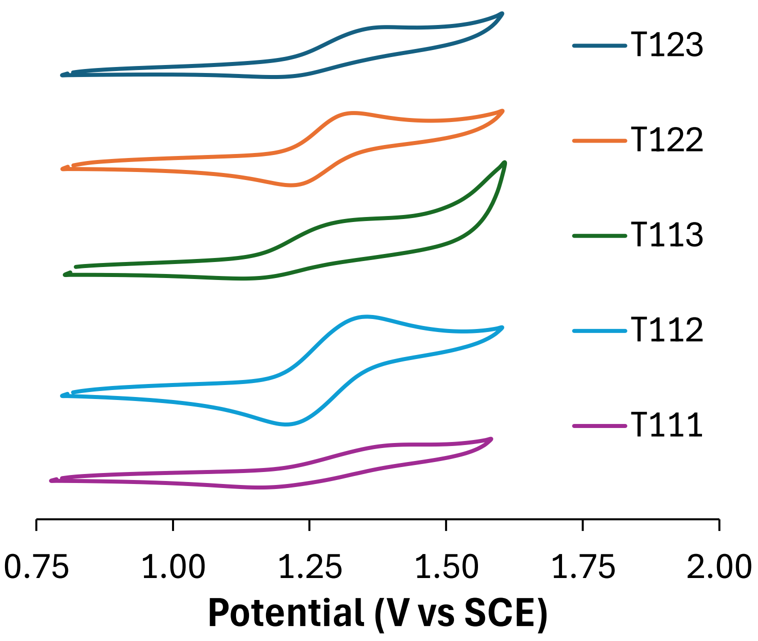

Figure S3.3 Cyclic voltammograms of prepared iron(II) supramolecular complexes (0.2 mM in acetonitrile) measured at 100 mV s_{-1}, 100 mM [TBA]PF_6 supporting electrolyte.

4. References

[1] J. J. Stone, R. A. Stockland, N. P. Rath, *Inorganica Chim. Acta* **2003**, *342*, 236-240.

[2] M. Juríček, M. Felici, P. Contreras-Carballada, J. Lauko, S. R. Bou, P. H. J. Kouwer, A. M. Brouwer, A. E. Rowan, *J. Mater. Chem.* **2011**, *21*, 2104-2111.

[3] D. Preston, A. Fox-Charles, W. K. C. Lo, J. D. Crowley, *Chem. Commun.* **2015**, *51*, 9042-9045.

[4] D. Aragao, J. Aishima, H. Cherukuvada, R. Clarken, M. Clift, N. P. Cowieson, D. J. Ericsson, C. L. Gee, S. Macedo, N. Mudie, S. Panjikar, J. R. Price, A. Riboldi-Tunnicliffe, R. Rostan, R. Williamson, T. T. Caradoc-Davies, *J. Synchrotron Radiat.* **2018**, *25*, 885-891.

[5] W. Kabsch, *J. Appl. Cryst.* **1993**, *26*, 795-800.

[6] G. M. Sheldrick, *Acta Crystallogr., Sect. A: Found. Crystallogr.* **2008**, *64*, 112-122.

[7] O. V. Dolomanov, L. J. Bourhis, R. J. Gildea, J. A. K. Howard, H. Puschmann, *J. Appl. Crystallogr.* **2009**, *42*, 339-341.

[8] A. J. Thompson, K. M. L. Smith, J. K. Clegg, J. R. Price, *J. Appl. Crystallogr.* **2023**, *56*, 558-564.

[9] in *Spartan '16*, Wavefunction, Inc., Irvine, CA, **2016**.

[10] S. Grimme, C. Bannwarth, P. Shushkov, *J. Chem. Theory Comput.* **2017**, *13*, 1989-2009.

[11] F. Neese, *WIREs Comput. Mol. Sci.* **2022**, *12*, e1606.

[12] S. Grimme, A. Hansen, S. Ehlert, J.-M. Mewes, *J. Chem. Phys.* **2021**, *154*.

[13] F. Weigend, R. Ahlrichs, *PCCP* **2005**, *7*, 3297-3305.

[14] P. Pollak, F. Weigend, *J. Chem. Theory Comput.* **2017**, *13*, 3696-3705.

[15] N. G. Connelly, W. E. Geiger, *Chem. Rev.* **1996**, *96*, 877-910.
